# *Armeniacae semen amarum*: a review on its botany, phytochemistry, pharmacology, clinical application, toxicology and pharmacokinetics

**DOI:** 10.3389/fphar.2024.1290888

**Published:** 2024-01-23

**Authors:** Shun Tang, Minmin Wang, Yuhui Peng, Yuanjing Liang, Jiarong Lei, Qiu Tao, Tianqi Ming, Yanqiao Shen, Chuantao Zhang, Jinlin Guo, Haibo Xu

**Affiliations:** ^1^ State Key Laboratory of Southwestern Chinese Medicine Resources, Department of Pharmacology, School of Pharmaceutical Sciences, Chengdu University of Traditional Chinese Medicine, Chengdu, China; ^2^ Department of Respiratory Medicine, Hospital of Chengdu University of Traditional Chinese Medicine, Chengdu, China; ^3^ State Key Laboratory of Southwestern Chinese Medicine Resources, School of Medical Technology, Chengdu University of Traditional Chinese Medicine, Chengdu, China

**Keywords:** *Armeniacae semen amarum*, *Prunus armeniaca* L., traditional Chinese medicine, ethnopharmacology, phytochemistry, pharmacology, clinical application, toxicology

## Abstract

*Armeniacae semen amarum*—seeds of *Prunus armeniaca* L. (Rosaceae) (ASA), also known as Kuxingren in Chinese, is a traditional Chinese herbal drug commonly used for lung disease and intestinal disorders. It has long been used to treat coughs and asthma, as well as to lubricate the colon and reduce constipation. ASA refers to the dried ripe seed of diverse species of Rosaceae and contains a variety of phytochemical components, including glycosides, organic acids, amino acids, flavonoids, terpenes, phytosterols, phenylpropanoids, and other components. Extensive data shows that ASA exhibits various pharmacological activities, such as anticancer activity, anti-oxidation, antimicrobial activity, anti-inflammation, protection of cardiovascular, neural, respiratory and digestive systems, antidiabetic effects, and protection of the liver and kidney, and other activities. In clinical practice, ASA can be used as a single drug or in combination with other traditional Chinese medicines, forming ASA-containing formulas, to treat various afflictions. However, it is important to consider the potential adverse reactions and pharmacokinetic properties of ASA during its clinical use. Overall, with various bioactive components, diversified pharmacological actions and potent efficacies, ASA is a promising drug that merits in-depth study on its functional mechanisms to facilitate its clinical application.

## 1 Introduction

*Armeniacae semen amarum*—seeds of *Prunus armeniaca* L. (Rosaceae) (ASA), also known as bitter almond or apricot kernel and Kuxingren in Chinese, is a widely used traditional Chinese herbal drug. It is renowned for its effectiveness in treating lung and intestinal diseases (Wei et al., 2023). In traditional Chinese medicine, it is commonly prescribed for relieving cough and asthma, as well as moisturizing the intestine to alleviate constipation (Gao et al., 2014). Modern studies have shown that ASA has a diverse range of pharmacological effects, including alleviating cough and resolving phlegm, as well as immunomodulation and anti-inflammatory properties (Ma et al., 2021; Zhao Y. et al., 2022). Meanwhile, both clinical and animal experiments have demonstrated that the effective components and prescriptions of ASA have significant therapeutic effects on respiratory diseases (Si and Zhang, 2021; Wang et al., 2023).

ASA is composed of various chemical components including glycosides, organic acids, amino acids, flavonoids, terpenes, phytosterols, phenylpropanoids, and other substances. The abundance of these active components makes ASA a valuable subject for research and application. Amygdalin, as the main active ingredient in ASA, has been found to have beneficial effects in relieving cough and asthma, as well as exhibiting anti-inflammatory and anti-fibrotic properties, which makes it a promising candidate for the treatment of respiratory diseases, with significant potential for disease management (Wang et al., 2021). Numerous studies have demonstrated the positive effects of ASA and its active ingredients on various respiratory conditions, including cough, asthma, chronic obstructive pulmonary disease (COPD), pulmonary heart disease, and lung function injury. Moreover, recent research has also suggested its potential role in treating COVID-19 (Luo et al., 2020; Zhou et al., 2020). Furthermore, ASA can be combined with other treatments to enhance its efficacy (Li et al., 2021; Noureen et al., 2022).

Although considerable studies have been performed on the ASA (Wei et al., 2023), there is still a lack of comprehensive and in-depth review of ASA. Herein, we conducted a comprehensive literature search using online databases such as PubMed, Web of Science, China National Knowledge Infrastructure (CNKI), and Google Scholar, with the keywords including ASA, its bioactive components, or ASA-containing formulas, up to December 2023. Then, we systematically summarize and highlight the botanical features and traditional uses, phytochemical components, pharmacological activities, clinical applications, toxicological effects including adverse reactions and detoxification methods, and pharmacokinetic characteristics of ASA, attempting to lay a foundation for the in-depth basic research on ASA and expanding its application in the clinical settings.

## 2 Botanical features and traditional uses

ASA, as defined in the 2020 edition of Chinese Pharmacopoeia, refers to the dried ripe seeds of various species of Rosaceae, namely, *P. armeniaca* L.var.*ansu* Maxim., *Prunus sibirica* L., *Prunus mandshurica* (Maxim.) Koehne, or *P. armeniaca* L.

**FIGURE 1:**
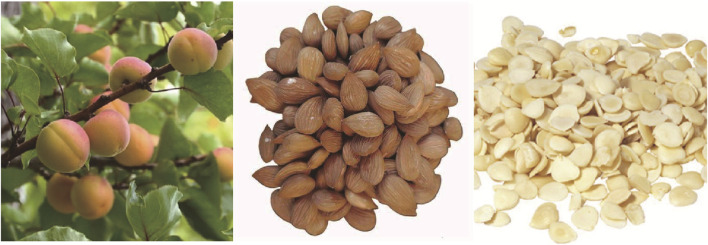
*P. armeniaca* and processed products of *Armeniacae semen amarum*.

It is recommended to harvest fully ripe fruits in the summer and extract their seeds by removing the pulp and core shell. The seeds should then be dried under the Sun. ASA, which contains cyanogenic components (Kovacikova et al., 2019), is known to have beneficial properties and minor toxicity. In traditional Chinese medicine, it is believed that ASA affects the lung and large intestine meridian. The Chinese Pharmacopoeia 2020 states that ASA has therapeutic effects such as lowering Qi, relieving cough and asthma, moisturizing the intestine, and relaxing the bowels (Wei et al., 2023) (Figure 1).

ASA was first documented in *Shennong*’*s Herbal* (Shen Nong Ben Cao Jing). It has a sweet taste and warm nature, primarily used for alleviating coughs caused by Qi. However, according to *Miscellaneous Records of Famous Physicians* (Ming Yi Bie Lu), ASA is described as having a bitter and toxic taste, commonly used to treat distress below the heart, abdominal fullness and distention, and occasionally headaches (Xue et al., 2022). The *essentials of Materia Medica* (Ben Cao Bei Yao) states that ASA is bitter in taste and warm in nature, with the ability to dissipate cold and alleviate irritable heat and shortness of breath. The *Compendium of Materia Medica* (Ben Cao Gang Mu) further indicates that ASA has various effects such as dispersing and reducing energy, relieving muscle and dispelling wind, reducing the Qi and moistening dryness, eliminating food stagnation, and treating injuries. Additionally, ASA has been found to have the potential of treating sores and repelling insects due to its toxicity. The book *Materia Medica Companion* (Ben Cao Meng Quan) describes its properties in further detail. However, it is important to note that ASA should not be used in conjunction with *Astragali radix*—roots of *Astragalus mongholicus* Bunge (Fabaceae), *Scutellariae radix*—roots of *Scutellaria baicalensis* Georgi (Lamiaceae), and *Puerariae lobatae radix*—roots of *Pueraria lobata* Ohwi (Fabaceae). ASA is commonly used for coughs with phlegm, constipation, and insect bites. It is worth mentioning that the treatment for constipation varies depending on whether it is related to Qi or blood deficiency. ASA is used for addressing Qi deficiency, while *Persicae semen*—seeds of *Prunus persica* (L.) Batsch (Rosaceae) is employed to promote blood circulation. In cases of Qi deficiency and a floating pulse, a combination of ASA and *Citri reticulatae pericarpium*—epicarps of *Citrus reticulata* Blanco (Rutaceae) is recommended. On the other hand, combining *P. semen* with *C. reticulatae pericarpium* is advised for addressing blood deficiency and a sinking pulse (Du and Yu, 2023).

## 3 Phytochemical components

**FIGURE 2:**
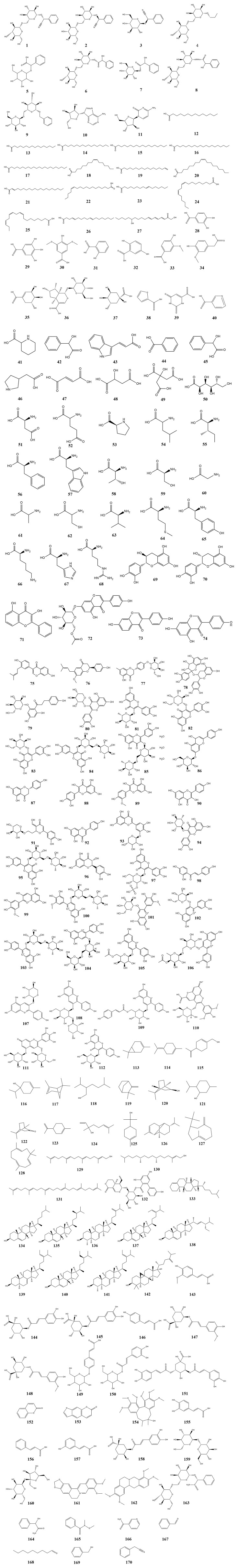
Chemical structures of compounds isolated from *Armeniacae semen amarum*.

Numerous studies have shown that ASA contains a variety of bioactive components and nutrients including glycosides, organic acids, amino acids, flavonoids, terpenes, phytosterols, phenylpropanoids, and other compounds. This section presents a compilation of literature on the chemical composition of ASA, providing detailed information on 170 major chemical components that have been isolated from it (Table 1). Furthermore, we have depicted the chemical structures of the main active components found in ASA (Figure 2).

**TABLE 1:** Chemical components isolated and structurally identified from ASA.

No.	Chemical constituent	Molecular formula	Extract	Method	Reference
**Glycosides**					
1	Amygdalin	C_20_H_27_NO_11_	Ethylacetate; ethanol	HPLC-PDA-ESI/MS	Hrichi et al. (2020)
			Methanol	HPLC-ESI-MS/MS	Xu et al. (2017)
			Methanol/water (7:3)	HPLC-ESI-MS	Senica et al. (2017)
2	Neoamygdalin	C_20_H_27_NO_11_	Methanol	HPLC-ESI-MS/MS	Xu et al. (2017)
			Methanol/water (7:3)	HPLC-ESI-MS	Senica et al. (2017)
3	Prunasin	C_14_H_17_NO_6_	Methanol	UPLC-MS/MS	Chen et al. (2022b)
			Methanol	HPLC-Q-TOF MS	Zhou et al. (2021)
			Methanol/water (7:3)	HPLC-ESI-MS	Senica et al. (2017)
4	Propyl-β-gentiobioside	C_15_H_28_O_11_	Methanol	HPLC-Q-TOF MS	Zhou et al. (2021)
5	Mandelic acid-β-glucopyranoside	C_14_H_18_O_8_	Methanol	HPLC-Q-TOF MS	Zhou et al. (2021)
6	Mandelic acid-β-gentiobioside	C_20_H_28_O_13_	Methanol	UPLC-MS/MS	Chen et al. (2022b)
				HPLC-Q-TOF MS	Zhou et al. (2021)
7	Mandelic acid amide-β-glucopyranoside	C_14_H_19_NO_7_	Methanol	UPLC-MS/MS	Chen et al. (2022b)
				HPLC-Q-TOF MS	Zhou et al. (2021)
8	Mandelic acid amide-β-gentiobioside	C_20_H_29_NO_12_	Methanol	UPLC-MS/MS	Chen et al. (2022b)
				HPLC-Q-TOF MS	Zhou et al. (2021)
9	Benzyl-β-gentiobioside	C_19_H_28_O_11_	Methanol	UPLC-MS/MS	Chen et al. (2022b)
				HPLC-Q-TOF MS	Zhou et al. (2021)
10	Adenosine	C_10_H_13_N_5_O_4_	Methanol	UPLC-MS/MS	Chen et al. (2022b)
				HPLC-Q-TOF MS	Zhou et al. (2021)
11	Cytarabine	C_9_H_13_N_3_O_5_	Methanol	UPLC-MS/MS	Chen et al. (2022b)
**Organic acids**					
*Fatty acids*					
12	Myristic acid	C_14_H_28_O_2_	Ethylacetate; ethanol; Dichloromethane; chloroform	GC-FID/MS	Hrichi et al. (2020)
13	Palmitic acid	C_16_H_32_O_2_	Ethylacetate; ethanol; Dichloromethane; chloroform	GC-FID/MS	Hrichi et al. (2020)
14	Heptadecanic acid	C_17_H_34_O_2_	Ethylacetate; ethanol; Dichloromethane; chloroform	GC-FID/MS	Hrichi et al. (2020)
15	Stearic acid	C_18_H_36_O_2_	Ethylacetate; ethanol; Dichloromethane; chloroform	GC-FID/MS	Hrichi et al. (2020)
16	Arachidic acid	C_20_H_40_O_2_	Ethylacetate; ethanol; Dichloromethane; chloroform	GC-FID/MS	Hrichi et al. (2020)
17	Behenic acid	C_22_H_44_O_2_	Ethylacetate; ethanol; Dichloromethane; chloroform	GC-FID/MS	Hrichi et al. (2020)
18	Palmitoleic acid	C_16_H_30_O_2_	Ethylacetate; ethanol; Dichloromethane; chloroform	GC-FID/MS	Hrichi et al. (2020)
19	Heptadecenoic acid	C_17_H_32_O_2_	Ethylacetate; ethanol	GC-FID/MS	Hrichi et al. (2020)
			Dichloromethane; chloroform		
20	Oleic acid	C_18_H_34_O_2_	Ethylacetate; ethanol; Dichloromethane; chloroform	GC-FID/MS	Hrichi et al. (2020)
21	Eicosenoic acid	C_20_H_38_O_2_	Ethylacetate; ethanol; Dichloromethane; chloroform	GC-FID/MS	Hrichi et al. (2020)
22	cis-13-Octadecenoic acid	C_18_H_34_O_2_	H_2_O	GC-MS	Shao et al. (2022)
23	9-Hexadecenoic acid	C_16_H_30_O	Aether	GC-MS	Zhang et al. (2007)
24	Linoleic acid	C_18_H_32_O_2_	Ethylacetate; ethanol; Dichloromethane; chloroform	GC-FID/MS	Hrichi et al. (2020)
25	Linolenic acid	C_18_H_30_O_2_	Ethylacetate; ethanol; Dichloromethane; chloroform	GC-FID/MS	Hrichi et al. (2020)
26	10-Octadecadienoic acid	C_18_H_32_O_2_	Aether	GC-MS	Zhang et al. (2007)
27	Hydroxy-octadecatrienoic acid	C_18_H_32_O_3_	Methanol	HPLC-Q-TOF MS	Zhou et al. (2021)
*phenolic acids*					
28	Protocatechuic acid	C_7_H_6_O_4_	Ethylacetate; ethanol	HPLC–PDA–ESI/MS	Hrichi et al. (2020)
29	Gallic acid	C_7_H_6_O_5_	Ethylacetate; ethanol	HPLC–PDA–ESI/MS	Hrichi et al. (2020)
30	Syringic acid	C_9_H_10_O_5_	Methanol and then n-hexane	HPLC	Qin et al. (2019)
31	Salicylic acid	C_7_H_6_O_3_	Ethanol	LC-ESI/MS	Cecarini et al. (2022)
32	Gentisic acid	C_7_H_6_O_4_	Ethanol	LC-ESI/MS	Cecarini et al. (2022)
33	Vanillic acid	C_8_H_8_O_4_	Ethanol	LC-ESI/MS	Cecarini et al. (2022)
34	Homovanillic acid	C_9_H_10_O_4_	Ethanol	LC-ESI/MS	Cecarini et al. (2022)
35	Shikimic acid	C_7_H_10_O_5_	H_2_O	LC-ESI/MS	Al-Juhaimi et al. (2021)
36	Loganic acid	C_16_H_24_O_10_	H_2_O	LC-ESI/MS	Al-Juhaimi et al. (2021)
*Other acids*					
37	Quinic acid	C_7_H_12_O_6_	Methanol	UPLC-MS/MS	Chen et al. (2022b)
38	2-Furoic acid	C_5_H_4_O_3_	Methanol	UPLC-MS/MS	Chen et al. (2022b)
39	Orotic acid	C_5_H_4_N_2_O_4_	Methanol	UPLC-MS/MS	Chen et al. (2022b)
40	Nicotinic acid	C_6_H_5_NO_2_	Methanol	UPLC-MS/MS	Chen et al. (2022b)
41	Pipecolic acid	C_6_H_11_NO_2_	Methanol	UPLC-MS/MS	Chen et al. (2022b)
42	Mandelic acid	C_8_H_8_O_3_	Methanol	UPLC-MS/MS	Chen et al. (2022b)
43	Indoleacrylic acid	C_11_H_9_NO_2_	Methanol	UPLC-MS/MS	Chen et al. (2022b)
44	Benzoic acid	C_7_H_6_O_2_	H_2_O	GC-MS	(Geng et al., 2016; Li et al., 2016)
45	Benzeneacetic acid, alpha-hydroxy-, (S)	C_8_H_8_O_3_	H_2_O	GC-MS	Shao et al. (2022)
46	3-Pyrrolidineacetic acid or isomer	C_6_H_11_NO_2_	Methanol	HPLC-Q-TOF MS	Zhou et al. (2021)
47	Fumaric acid	C_4_H_4_O_4_	Methanol	UPLC-MS/MS	Chen et al. (2022b)
48	Malic acid	C_4_H_6_O_5_	Methanol	UPLC-MS/MS	Chen et al. (2022b)
49	Citric acid	C_6_H_8_O_7_	Methanol	UPLC-MS/MS	Chen et al. (2022b)
50	Gluconic acid	C_6_H_12_O_7_	Methanol	HPLC-Q-TOF MS	Zhou et al. (2021)
**Amino acids**					
51	Aspartic acid	C_4_H_7_NO_4_	Methanol	UPLC-MS/MS	Chen et al. (2022b)
52	Glutamic acid	C_5_H_9_NO_4_	Methanol	UPLC-MS/MS	Chen et al. (2022b)
53	Proline	C_5_H_9_NO_2_	Methanol	UPLC-MS/MS	Chen et al. (2022b)
				HPLC-Q-TOF MS	Zhou et al. (2021)
54	Leucine	C_6_H_13_NO_2_	Methanol	HPLC-Q-TOF MS	Zhou et al. (2021)
55	Isoleucine	C_6_H_13_NO_2_	Methanol	UPLC-MS/MS	Chen et al. (2022b)
				HPLC-Q-TOF MS	Zhou et al. (2021)
56	Phenylalanine	C_9_H_11_NO_2_	Methanol	UPLC-MS/MS	Chen et al. (2022b)
				HPLC-Q-TOF MS	Zhou et al. (2021)
57	Tryptophan	C_11_H_12_N_2_O_2_	Methanol	UPLC-MS/MS	Chen et al. (2022b)
				HPLC-Q-TOF MS	Zhou et al. (2021)
58	Threonine	C_4_H_9_NO_3_	HCL	Automatic amino acid analyzer	Li et al. (2004)
59	Serine	C_3_H_7_NO_3_	HCL	Automatic amino acid analyzer	Li et al. (2004)
60	Glycine	C_2_H_5_NO_2_	HCL	Automatic amino acid analyzer	Li et al. (2004)
61	Alanine	C_3_H_7_NO_2_	HCL	Automatic amino acid analyzer	Li et al. (2004)
62	Cysteine	C_3_H_7_NO_2_S	HCL	Automatic amino acid analyzer	Li et al. (2004)
63	Valine	C_5_H_11_NO_2_	HCL	Automatic amino acid analyzer	Li et al. (2004)
64	Methionine	C_5_H_11_O_2_NS	HCL	Automatic amino acid analyzer	Li et al. (2004)
65	Tyrosine	C_9_H_11_NO_3_	HCL	Automatic amino acid analyzer	Li et al. (2004)
66	Lysine	C_6_H_14_N_2_O_2_	HCL	Automatic amino acid analyzer	Li et al. (2004)
67	Histidine	C_6_H_9_N_3_O_2_	HCL	Automatic amino acid analyzer	Li et al. (2004)
68	Arginine	C_6_H_14_N_4_O_2_	HCL	Automatic amino acid analyzer	Li et al. (2004)
**Flavonoids**					
69	Catechin	C_15_H_14_O_6_	Ethylacetate; ethanol	HPLC–PDA–ESI/MS	Hrichi et al. (2020)
			Methanol/water (7:3)	HPLC-ESI-MS	Senica et al. (2017)
70	Epicatechin	C_15_H_14_O_6_	Ethylacetate; ethanol	HPLC–PDA–ESI/MS	Hrichi et al. (2020)
			Methanol/water (7:3)	HPLC-ESI-MS	Senica et al. (2017)
71	Dimethoxyflavone	C_17_H_14_O_4_	Ethylacetate; ethanol	HPLC–PDA–ESI/MS	Hrichi et al. (2020)
72	Acetylgenistin	C_23_H_22_O_11_	Ethylacetate; ethanol	HPLC–PDA–ESI/MS	Hrichi et al. (2020)
73	Daidzein	C_15_H_10_O_4_	Methanol	UPLC-MS/MS	Chen et al. (2022b)
74	Genistein	C_15_H_10_O_5_	Methanol	UPLC-MS/MS	Chen et al. (2022b)
75	Neobavaisoflavone	C_20_H_18_O_4_	Methanol	UPLC-MS/MS	Chen et al. (2022b)
76	Bavachinin	C_21_H_22_O_4_	Methanol	UPLC-MS/MS	Chen et al. (2022b)
77	Naringenin hexoside	C_27_H_32_O_14_	Methanol/water (7:3)	HPLC-ESI-MS	Senica et al. (2017)
78	Procyanidin dimer	C_30_H_26_O_12_	Methanol/water (7:3)	HPLC-ESI-MS	Senica et al. (2017)
79	Phloridzin	C_21_H_24_O_10_	Methanol/water (7:3)	HPLC-ESI-MS	Senica et al. (2017)
80	Quercetin-3-xyloside	C_20_H_18_O_11_	Methanol/water (7:3)	HPLC-ESI-MS	Senica et al. (2017)
81	Quercetin-3-rhamnoside	C_21_H_20_O_11_	Methanol/water (7:3)	HPLC-ESI-MS	Senica et al. (2017)
82	Quercetin-3-galactoside	C_21_H_20_O_12_	Methanol/water (7:3)	HPLC-ESI-MS	Senica et al. (2017)
83	Quercetin-3-glucoside	C_21_H_20_O_12_	Methanol/water (7:3)	HPLC-ESI-MS	Senica et al. (2017)
84	Quercetin-3-rutinoside	C_27_H_30_O_16_	Methanol/water (7:3)	HPLC-ESI-MS	Senica et al. (2017)
85	Rutin trihydrate	C_27_H_36_O_19_	Methanol and then n-hexane	HPLC	Qin et al. (2019)
86	Apigenin-7-glucoside	C_21_H_20_O_10_	Methanol and then n-hexane	HPLC	Qin et al. (2019)
87	Naringenin	C_15_H_12_O_5_	Methanol and then n-hexane	HPLC	Qin et al. (2019)
88	Quercetin	C_15_H_10_O_7_	Methanol and then n-hexane	HPLC	Qin et al. (2019)
89	Isorhamnetin	C_16_H_12_O_7_	Methanol and then n-hexane	HPLC	Qin et al. (2019)
90	Kaempferol	C_15_H_10_O_6_	Methanol and then n-hexane	HPLC	Qin et al. (2019)
91	Luteolin 7-xyloside	C_20_H_18_O_10_	Ethanol	LC-ESI/MS	Cecarini et al. (2022)
92	Apigenin	C_15_H_10_O_5_	Ethanol	LC-ESI/MS	Cecarini et al. (2022)
93	Tricetin 3′-xyloside	C_20_H_18_O_11_	Ethanol	LC-ESI/MS	Cecarini et al. (2022)
94	Quercitrin	C_21_H_20_O_11_	Ethanol	LC-ESI/MS	Cecarini et al. (2022)
95	Rutin	C_27_H_30_O_16_	Ethanol	LC-ESI/MS	Cecarini et al. (2022)
96	(±)Taxifolin	C_15_H_12_O_7_	Ethanol	LC-ESI/MS	Cecarini et al. (2022)
97	Quercetin 3-(3″-sulfatoglucoside)	C_21_H_20_O_15_S	Ethanol	LC-ESI/MS	Cecarini et al. (2022)
98	Isoliquiritigenin	C_15_H_12_O_4_	Ethanol	LC-ESI/MS	Cecarini et al. (2022)
99	Petunidin	C_16_H_13_O_7_	Ethanol	LC-ESI/MS	Cecarini et al. (2022)
100	Petunidin 3-rutinoside	C_28_H_33_O_16_	Ethanol	LC-ESI/MS	Cecarini et al. (2022)
101	Petunidin 3-galactoside	C_22_H_23_O_12_	Ethanol	LC-ESI/MS	Cecarini et al. (2022)
102	Cyanidin 3-O-galactoside	C_21_H_21_O_11_	Ethanol	LC-ESI/MS	Cecarini et al. (2022)
103	Cyanidin 3-rutinoside	C_27_H_31_O_15_	Ethanol	LC-ESI/MS	Cecarini et al. (2022)
104	Cyanidin 3-glucogalactoside	C_27_H_31_O_16_	Ethanol	LC-ESI/MS	Cecarini et al. (2022)
105	Cyanidin 3-(6-acetylgalactoside)	C_23_H_23_O_12_	Ethanol	LC-ESI/MS	Cecarini et al. (2022)
106	Cyanidin 3-(4″- acetylrutinoside)	C_29_H_33_O_16_	Ethanol	LC-ESI/MS	Cecarini et al. (2022)
107	Pelargonidin 3-arabinoside	C_20_H_19_O_9_	Ethanol	LC-ESI/MS	Cecarini et al. (2022)
108	Pelargonidin 3-lathyroside	C_26_H_29_O_14_	Ethanol	LC-ESI/MS	Cecarini et al. (2022)
109	Pelargonidin 3-p-coumarylglucoside	C_30_H_27_O_12_	Ethanol	LC-ESI/MS	Cecarini et al. (2022)
110	Malvidin 3-glucoside-pyruvate	C_26_H_25_O_14_	Ethanol	LC-ESI/MS	Cecarini et al. (2022)
111	Delphinidin-3,5-diglucoside	C_27_H_30_O_17_	H_2_O	LC-ESI/MS	Al-Juhaimi et al. (2021)
112	Kaempferol-3- glucoside	C_21_H_20_O_11_	H_2_O	LC-ESI/MS	Al-Juhaimi et al. (2021)
**Terpenoids**					
*Monoterpenoids*					
113	3-Carene	C_10_H_16_	H_2_O	GC-MS	Shao et al. (2022)
114	Cyclohexene, 1-methyl-4-(1-methylethylidene)	C_10_H_16_	H_2_O	GC-MS	Shao et al. (2022)
115	1-Cyclohexene-1-methanol, 4-(1-methylethenyl)	C_10_H_16_O	H_2_O	GC-MS	Shao et al. (2022)
116	3-Cyclohexen-1-ol, 4-methyl-1-(1-methylethyl)	C_10_H_18_O	H_2_O	GC-MS	Shao et al. (2022)
117	Alpha-Pinene	C_10_H_16_	1,2,3-trichloropropane	GC-MS	Jin et al. (2018)
118	2-Isopropyl-5-methylhexan-1-ol	C_10_H_22_O	1,2,3-trichloropropane	GC-MS	Jin et al. (2018)
119	Camphene	C_10_H_16_	1,2,3-trichloropropane	GC-MS	Jin et al. (2018)
120	Borneol	C_10_H_18_O	Deionized water	GC-MS	Hui et al. (2003)
121	Menthol	C_10_H_20_O	Deionized water	GC-MS	Hui et al. (2003)
122	Camphor	C_10_H_16_O	Deionized water	GC-MS	Hui et al. (2003)
			Not mentioned	HS-GC-MS	Chen et al. (2023)
123	Cinene	C_10_H_16_	Not mentioned	HS-GC-MS	Chen et al. (2023)
124	Linalool	C_10_H_18_O	Not mentioned	HS-GC-MS	Chen et al. (2023)
125	Terpineol	C_10_H_18_O	Not mentioned	HS-GC-MS	Chen et al. (2023)
*Sesquiterpenes*					
126	Copaene	C_15_H_24_	H_2_O	GC-MS	Shao et al. (2022)
127	Caryophyllene	C_15_H_24_	Deionized water	GC-MS	Hui et al. (2003)
128	α-Caryophyllene	C_15_H_24_	Deionized water	GC-MS	Hui et al. (2003)
*Diterpenoids*					
129	trans-Geranylgeraniol	C_20_H_34_O	H_2_O	GC-MS	Li et al. (2016)
130	Phytol	C_20_H_40_O	H_2_O	GC-MS	Shao et al. (2022)
*Triterpenoids*					
131	Squalene	C_30_H_50_	n-hexane	TLC and capillary GLC	Rudzińska et al. (2017)
132	Amarogentin	C_29_H_30_O_13_	H_2_O	LC-ESI/MS	Al-Juhaimi et al. (2021)
**Phytosterols**					
133	Cholest-4-ene	C_27_H_46_	H_2_O	GC-MS	Li et al. (2016)
134	cholesterol	C_27_H_46_O	n-hexane	TLC and capillary GLC	Rudzińska et al. (2017)
135	campesterol	C_28_H_48_O	n-hexane	TLC and capillary GLC	Rudzińska et al. (2017)
136	gramisterol	C_29_H_48_O	n-hexane	TLC and capillary GLC	Rudzińska et al. (2017)
137	Δ5-avenasterol	C_29_H_48_O	n-hexane	TLC and capillary GLC	Rudzińska et al. (2017)
138	Δ7-stigmasterol	C_29_H_48_O	n-hexane	TLC and capillary GLC	Rudzińska et al. (2017)
139	Δ7-Avenasterol	C_29_H_48_O	n-hexane	TLC and capillary GLC	Rudzińska et al. (2017)
140	β-sitosterol	C_29_H_50_O	n-hexane	TLC and capillary GLC	Rudzińska et al. (2017)
141	citrostadienol	C_30_H_50_O	n-hexane	TLC and capillary GLC	Rudzińska et al. (2017)
142	24-methylene-cycloartanol	C_31_H_52_O	n-hexane	TLC and capillary GLC	Rudzińska et al. (2017)
**Phenylpropanoids**					
143	Ferulic acid	C_10_H_10_O_4_	Ethylacetate; ethanol	HPLC–PDA–ESI/MS	Hrichi et al. (2020)
144	Chlorogenic acid	C_16_H_18_O_9_	Ethylacetate; ethanol	HPLC–PDA–ESI/MS	Hrichi et al. (2020)
			Methanol/water (7:3)	HPLC-ESI-MS	Senica et al. (2017)
145	Neochlorogenic acid	C_16_H_18_O_9_	Ethylacetate; ethanol	HPLC–PDA–ESI/MS	Hrichi et al. (2020)
			Methanol/water (7:3)	HPLC-ESI-MS	Senica et al. (2017)
146	p-Coumaric acid	C_9_H_8_O_3_	Methanol/water (7:3)	HPLC-ESI-MS	Senica et al. (2017)
147	3-Feruloylquinic acid	C_17_H_20_O_9_	Methanol/water (7:3)	HPLC-ESI-MS	Senica et al. (2017)
148	5-Feruloylquinic acid	C_17_H_20_O_9_	Methanol/water (7:3)	HPLC-ESI-MS	Senica et al. (2017)
149	p-Coumaric acid hexoside	C_15_H_18_O_8_	Methanol/water (7:3)	HPLC-ESI-MS	Senica et al. (2017)
150	Caffeic acid hexoside	C_15_H_18_O_9_	Methanol/water (7:3)	HPLC-ESI-MS	Senica et al. (2017)
151	Dicaffeoylquinic acid	C_25_H_24_O_12_	Methanol/water (7:3)	HPLC-ESI-MS	Senica et al. (2017)
152	Coumarin	C_9_H_6_O_2_	Ethylacetate; ethanol	HPLC–PDA–ESI/MS	Hrichi et al. (2020)
153	Psoralen	C_11_H_6_O_3_	Methanol	UPLC-MS/MS	Chen et al. (2022b)
154	Schisandrin	C_24_H_32_O_7_	Methanol	UPLC-MS/MS	Chen et al. (2022b)
155	Caffeic acid	C_9_H_8_O_4_	Methanol and then n-hexane	HPLC	Qin et al. (2019)
156	trans-cinnamic acid	C_9_H_8_O_2_	Methanol and then n-hexane	HPLC	Qin et al. (2019)
157	m-Coumaric acid	C_9_H_8_O_3_	Ethanol	LC-ESI/MS	Cecarini et al. (2022)
158	5-caffeylquinic acid	C_16_H_18_O_9_	H_2_O	LC-ESI/MS	Al-Juhaimi et al. (2021)
**Others**					
159	Trehalose	C_12_H_22_O_11_	Methanol	UPLC-MS/MS	Chen et al. (2022b)
160	Sucrose	C_12_H_22_O_11_	Methanol	HPLC-Q-TOF MS	Zhou et al. (2021)
161	Berberine	C_20_H_18_NO_4_	Methanol	UPLC-MS/MS	Chen et al. (2022b)
162	Tetrahydropalmatine	C_21_H_25_NO_4_	Methanol	UPLC-MS/MS	Chen et al. (2022b)
163	Amygdalin amide	C_20_H_29_NO_12_	Methanol	UPLC-MS/MS	Chen et al. (2022b)
164	Mandelamide	C_8_H_9_NO_2_	H_2_O	GC-MS	Shao et al. (2022)
165	N-Methoxy-N-methylbenzamide	C_9_H_11_NO_2_	H_2_O	GC-MS	Shao et al. (2022)
166	Nicotinamide	C_6_H_6_N_2_O	Methanol	UPLC-MS/MS	Chen et al. (2022b)
167	Benzaldehyde	C_7_H_6_O	H2O	GC-MS	Geng et al. (2016), Li et al. (2016), Shao et al. (2022)
168	Nonanal	C_9_H_18_O	H2O	GC-MS	Li et al. (2016), Shao et al. (2022)
169	Benzyl alcohol	C_7_H_8_O	H2O	GC-MS	Geng et al. (2016), Li et al. (2016), Shao et al. (2022)
170	Benzyl cyanide	C_8_H_7_N	H2O	GC-MS	Geng et al. (2016)

### 3.1 Glycosides

The glycosides found in ASA primarily consist of cyanogenic glycosides, which serve as both its main toxic components and its primary pharmacologically active ingredients. The principal glycoside in ASA is amygdalin **(1)**. It is important to note that consuming a large amount of amygdalin within a short period of time may lead to cyanide poisoning. This occurs due to the hydrolysis of amygdalin by β-D-glucosidase, leading to the production of benzaldehyde and hydrocyanic acid, which can cause respiratory depression (Song and Xu, 2014). Pharmacological studies have demonstrated that amygdalin exhibits significant anti-tumor activity, as well as antinociceptive and antiphlogistic effects, making it a promising candidate for various applications (Park et al., 2005; Hwang et al., 2008; Figurová et al., 2021; Guo et al., 2023; Zhang et al., 2023). In addition, another cyanogenic glycoside called neoamygdalin **(2)** has been isolated and identified from ASA. Neoamygdalin is an epimorphous isoform of amygdalin and shows great potential in the treatment of cough and asthma (Xu et al., 2017). Besides, mass spectrometry analysis has revealed the presence of amygdalin metabolites and its glycosides in ASA extracts, including prunasin **(3)**, mandelic acid-β-glucopyranoside **(5)**, mandelic acid-β-gentiobioside **(6)**, mandelic acid amide-β-glucopyranoside **(7)**, mandelic acid amide-β-gentiobioside **(8)**, and benzyl-β-gentiobioside **(9)**. Furthermore, ASA methanol extracts also contain propyl-β-gentiobioside **(4)**, adenosine **(10)** and cytarabine **(11)** (Chen Y. et al., 2022). The information of these glycosides is listed in Table 1, and the chemical structures were drawn by ChemDraw 20.0 and presented in Figure 2.

### 3.2 Organic acids

Currently, a total of 39 organic acids have been isolated and identified in ASA. Among them, **(12–27)** are fatty acids, accounting for approximately 50% of ASA (Jin et al., 2018), which can be divided into saturated fatty acids **(12–17)**, monounsaturated fatty acids **(18–23)**, and polyunsaturated fatty acids **(24–27)**. Notably, unsaturated fatty acids such as oleic acid **(20)**, linoleic acid **(24)**, and linolenic acid **(25)** are essential for the human body as they cannot be synthesized internally and must be obtained from food (Spector and Kim, 2015). Pharmacological studies have demonstrated that unsaturated fatty acids possess various beneficial effects such as regulation of thrombosis, immune modulation, and anti-fibrosis (Khosla and Fungwe, 2001; Vangaveti et al., 2016; Turolo et al., 2021), making them of significant medicinal value. In addition, ASA contains a range of phenolic acids **(28–36)**, which have antibacterial, anti-inflammatory, anti-oxidation and other pharmacological effects (Bak et al., 2013; Thakare et al., 2017). Furthermore, mandelic acid **(42)**, a metabolite of amygdalin, has been investigated for its antimicrobial activity and low vaginal irritation, particularly in the context of urinary tract infections and vaginal trichomoniasis (Xia et al., 2020). Other organic acids, including fumaric acid **(47)**, malic acid **(48)**, citric acid **(49)**, and gluconic acid **(50),** have also been isolated and identified from ASA. Information of these organic acids is listed in Table 1. The chemical structures were drawn by ChemDraw 20.0 and shown in Figure 2.

### 3.3 Amino acids

Protein is a crucial component of human cells and tissues. The human body contains numerous proteins with diverse functions, all of which are formed through the dehydration and condensation of amino acids. The protein content in ASA is more than 20%, and the content of important amino acids is reasonable and sufficient (Li et al., 2004). Currently, 18 amino acids **(51–68)** have been isolated and identified from ASA, among which leucine **(54)**, isoleucine **(55)**, phenylalanine **(56)**, tryptophan **(57)**, threonine **(58)**, methionine **(64)**, valine **(65)** and lysine **(66)** are essential amino acids, while histidine **(67)** is also an essential amino acid for infant growth. These amino acids are summarized in Table 1, and their chemical structures were drawn by ChemDraw 20.0 and presented in Figure 2.

### 3.4 Flavonoids

Flavonoids have various physiological effects such as antioxidant, anti-inflammatory, and improvement of cardiovascular function (Feng et al., 2016; Shen et al., 2022). However, the content of flavonoids in ASA is 14.81 mg/100 g, less than 2‰ (Tanwar et al., 2018). Until now, 43 flavonoids **(69–112)** have been isolated and characterized from ASA, among which catechin **(69)**, epicatechin **(70)**, rutin trihydrate **(85)**, apigenin-7-glucoside **(86)**, luteolin 7-xyloside **(91)**, apigenin **(92)**, tricetin 3′-xyloside **(93)** are flavanols. Dimethoxyflavone **(71)**, acetylgenistin **(72)**, daidzein **(73)**, genistein **(74)** and neobavaisoflavone **(75)** are isoflavones. Bavachinin **(76)**, naringenin hexoside **(77)**, procyanidin dimer **(78)** and isoliquiritigenin **(98)** are dihydroflavonoids. Phloridzin **(79)** and naringenin **(87)** are dihydrochalcones. Compounds **(80–84, 88–90, 94–97, 112)** are flavanols. Additionally, 12 anthocyanins **(99–111)** have been extracted from ASA skins, which belong to flavonoids as well (Qin et al., 2019; Cecarini et al., 2022). These flavonoids are summarized in Table 1, and their chemical structures were drawn using ChemDraw 20.0 and presented in Figure 2.

### 3.5 Terpenoids

Terpenoids, which consist of isoprene as the fundamental structural unit, are commonly found in Chinese herbal medicine and exhibit various pharmacological effects such as antioxidant, antimalarial, antibacterial, anti-inflammatory, and anti-cancer properties (Atriya et al., 2023). Currently, 20 terpenoids have been isolated and identified from ASA. These include 13 monoterpenoids **(113–125)**, 3 sesquiterpenoids **(126–128)**, two diterpenoids (trans-geranylgeraniol **(129)** and phytol **(130)**), and squalene **(131)**, which belongs to the triterpenoid group. Moreover, amarogentin **(132)**, a schizocyclic iridoterpenoid, has also been isolated from the aqueous extract of ASA. These terpenoids are summarized in Table 1, and their chemical structures were drawn by ChemDraw 20.0 and presented in Figure 2.

### 3.6 Physterols

The basic structure of sterols consists of cyclopentane polyhydrophenanthrene and a hydroxyl group. Phytosterols, a type of sterols, are commonly found in various parts of plants such as roots, stems, leaves, fruits, and seeds. Pharmacological studies have demonstrated the beneficial physiological effects of phytosterols, including their ability to prevent cardiovascular diseases, inhibit tumor growth, promote metabolism, and regulate hormone levels (Bakrim et al., 2022; Nattagh-Eshtivani et al., 2022). The total phytosterol content in different varieties of ASA ranges from 215.7 to 973.6 mg/100 g of bitter apricot kernel oil (Rudzińska et al., 2017). So far, researchers have isolated and identified 10 phytosterols **(133–142)** from ASA. In addition, Rudzińska Magdalena et al. analyzed the composition of ASA fat oil using TLC and capillary GLC methods, which revealed the presence of major phytosterols such as cholesterol **(134)**, campesterol **(135)**, gramisterol **(136)**, Δ5-avenasterol **(137)**, Δ7-stigmasterol **(138)**, Δ7-avenasterol **(139)**, β-sitosterol **(140)**, citrostadienol **(141)**, and 24-methylene-cycloartanol **(142)**. These physterols are summarized in Table 1. The corresponding chemical structures were drawn using ChemDraw 20.0 and presented in Figure 2.

### 3.7 Phenylpropanoids

The basic structural unit of phenylpropanoids consists of a benzene ring and three branched carbons (C6-C3). Until now, 16 phenylpropanoids have been successfully isolated and identified from ASA, among which **(143–151, 155–158)** are phenylpropanoic acids, coumarin **(152)** and psoralen **(153)** are coumarins, and schisandrin **(154)** is lignan. Besides, chlorogenic acid **(144)**, 5-feruloylquinic acid **(148)** and dicaffeoylquinic acid **(151)** are polyphenols with significant anti-oxidant activity and free radical scavenging activity (Iwai et al., 2004; Cao et al., 2010; Park et al., 2015). These phenylpropanoids are summarized in Table 1, and their chemical structures were drawn by ChemDraw 20.0 and presented in Figure 2 as well.

### 3.8 Others

Besides the chemical constituents mentioned above, other components have also been investigated and summarized in Table 1, and the corresponding chemical structures are drawn by ChemDraw 20.0 in Figure 2. In brief, trehalose **(159)** and sucrose **(160)** are saccharides, berberine **(161)** and tetrahydropalmatine **(162)** are alkaloids, amygdalin amide **(163)**, mandelamide **(164)**, N-methoxy-N-methylbenzamide **(165)** and nicotinamide **(166)** are amide compounds. Furthermore, the compounds **(167–170)** are the main ingredients in ASA volatile oil.

## 4 Pharmacological activities

**FIGURE 3:**
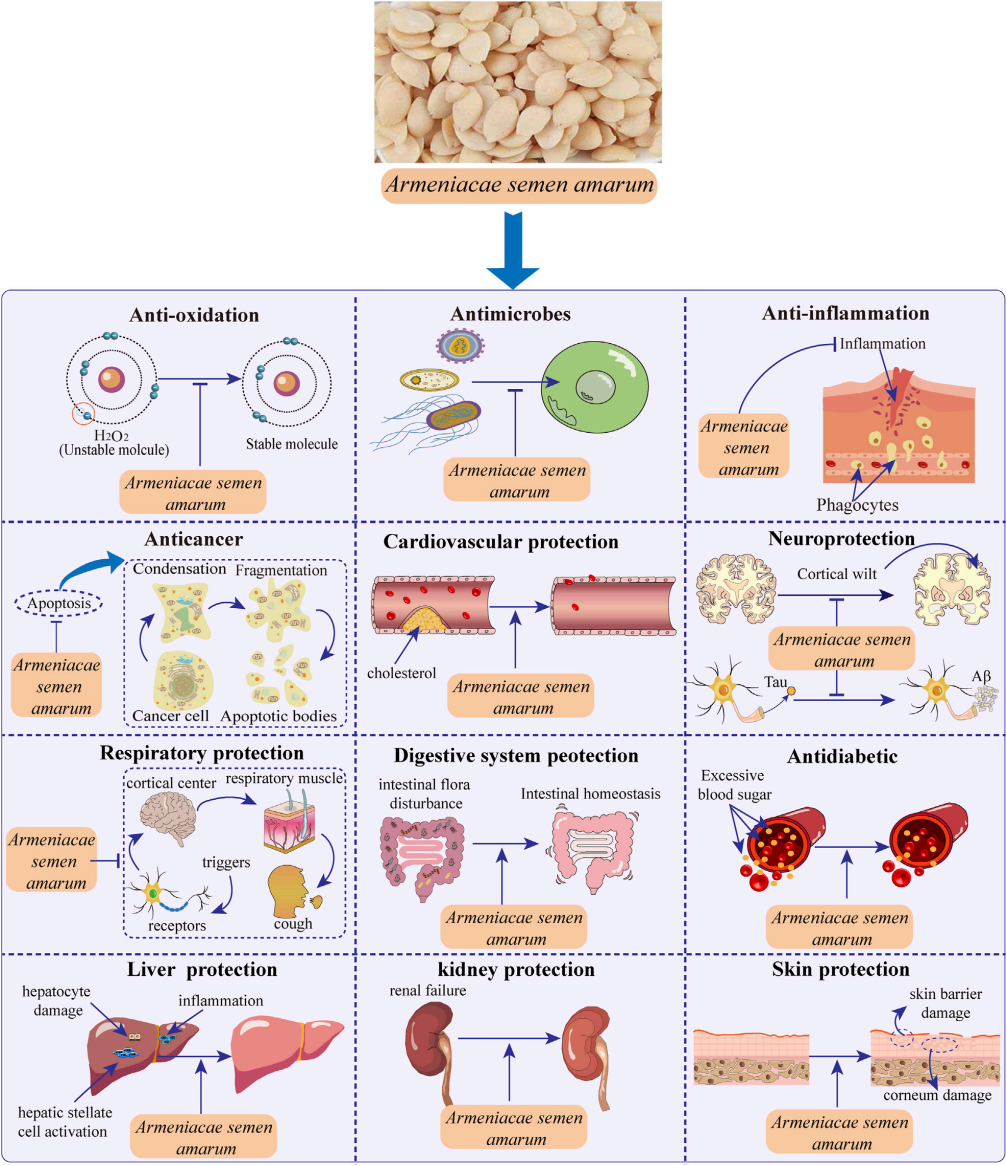
Pharmacological activities of *Armeniacae semen amarum*.

ASA exhibits a wide range of pharmacological activities and effects due to its abundance of chemical components and active substances. These include anticancer activity (breast carcinoma, prostatic cancer, hepatocellular carcinoma, lung cancer, renal cell carcinoma, bladder cancer and other cancers), anti-oxidant activity, antimicrobial activity, anti-inflammation activity, cardiovascular protection, neuroprotection, respiratory protection, digestive system protection, antidiabetic, liver and kidney protection, skin protection and other pharmacological activities (Figure 3). The following is a detailed introduction to the pharmacological effects of ASA.

### 4.1 Anticancer activity

**FIGURE 4:**
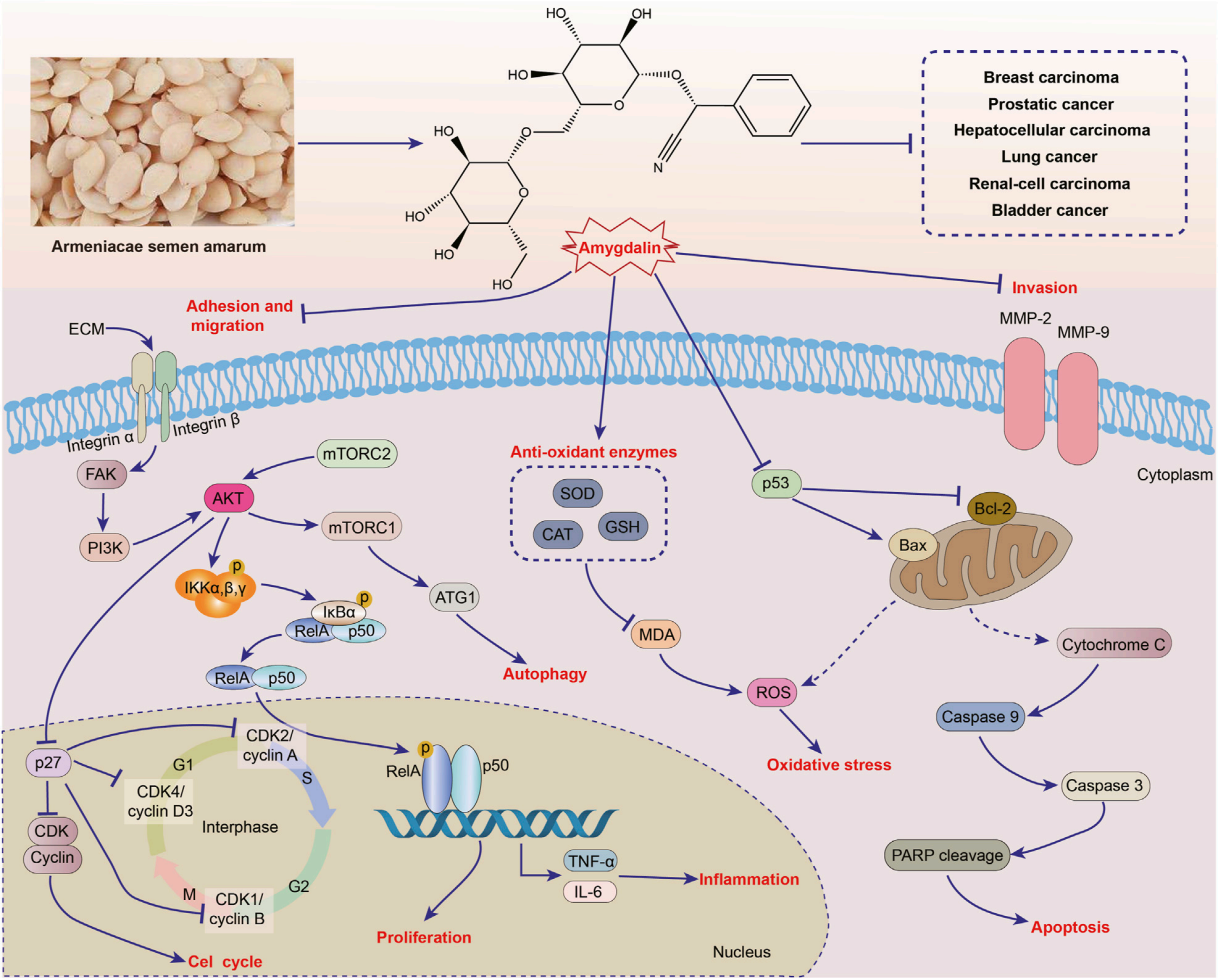
Anticancer activities of *Armeniacae semen amarum*.

In recent years, the overall incidence and mortality of cancer are still on the rise. Despite advances in various comprehensive therapies, the mortality rate of advanced malignant tumors remains high (Chen L. et al., 2021; Zhao et al., 2022a; Ming et al., 2022). ASA is rich in a variety of phytochemical ingredients, and amygdalin is one of its main active ingredients. Amygdalin is a phytochemical ingredient that has been extensively studied for its therapeutic effects on various types of cancers, including breast cancer, prostate cancer, hepatocellular carcinoma, renal cell carcinoma, lung cancer, bladder cancer, and others. Numerous studies have demonstrated the therapeutic potential of different ASA extracts and amygdalin. The therapeutic mechanism of ASA primarily involves inhibiting cancer cell adhesion, migration, and proliferation, as well as blocking the cell cycle, inducing cell oxidative damage and apoptosis, and regulating autophagy. However, it is important to note that the current research on the anticancer activity of ASA is mostly limited to *in vitro* cell studies, with fewer *in vivo* studies and a lack of clinical trials. Therefore, further investigation is needed to fully explore ASA as a potential alternative therapy for cancer. The effects of ASA and amygdalin on different types of cancer and their action mechanisms are summarized in Table 2 and Figure 4.

**TABLE 2:** Anticancer activity of ASA.

Types of cancer	Cell line/model	Compound	Minimal active concentration/dose	Mechanisms	Reference
Breast carcinoma	Hs578T cells	Amygdalin	10, 20, 40 mg/mL	Induction of apoptosis by intensifying the protein expressions of Bax and pp38 MAPK, while decreasing Bcl-2 and pro-caspase-3 protein expression; Decreasing adhesion via down-regulating integrin α5 protein expression	Lee and Moon (2016)
Breast carcinoma	MCF-7, MDA-MB-231 cells	Amygdalin	IC50 (MCF-7) = 34, 30 and 25 mg/mL for 24, 48 h and 72 h, respectively; IC50 (MDA-MB-231) = 28, 23.5 and 21 mg/mL for 24, 48 h and 72 h, respectively	Suppressing adhesion and migration, decreasing adhesion to fibronectin and collagen I, regulating mRNA levels of integrin α and β	Mosayyebi et al. (2021)
Breast carcinoma	MCF-7 cells	Amygdalin	IC50 = 200.6 and 197.9 μg/mL for 24 and 48 h, respectively	Inhibiting proliferation and blocking DNA replication	Albogami and Alnefaie (2021)
Breast carcinoma	MCF-7 and SK-BR-3 cells	Amygdalin	5, 10, 20 mg/mL	Inducing apoptosis, elevating Bax protein expression and descending Bcl-2 protein expression	Moradipoodeh et al. (2020)
Breast carcinoma	SK-BR-3 cells	Amygdalin	5, 10, 20 mg/mL	Inducing apoptosis, up-regulating Bax protein expression and down-regulating Bcl-2 protein expression	Moradipoodeh et al. (2019)
Breast carcinoma	MCF-7 cells	Amygdalin/ASA extracts	50 µM/1 mg/mL	Inhibition of autophagy cascade and migration due to downregulation of cathepsin B and L activities; Hampering the activities of proteasome 20S and 26S to induce apoptosis	Cecarini et al. (2022)
Breast carcinoma	MCF-7 and T47D cells	Amygdalin	65 mM	Induction of oxidative stress, Lowering MDA and GSSG levels, rising TGSH and GSH activities	Abboud et al. (2019)
Prostatic cancer	DU-145 cells	Amygdalin	10 mg/mL	Suppressing adhesion to HUVECs and immobilized collagen, repressing chemotaxis and migration; down-regulating integrin α6 protein expression while upregulating integrin α2 protein expression	Mani et al. (2020)
Prostatic cancer	LNCaP, DU-145 and PC3 cells	Amygdalin	10 mg/mL	Suppressing cell growth and promoting apoptosis, delaying cell cycle progression by repressing protein expression related to CDK1-cyclin B axis and AKT-mTOR pathway	Makarević et al. (2016)
Prostatic cancer	LNCaP and DU-145 cells	Amygdalin	0.1, 1 and 10 mg/mL	Promoting apoptosis by raising caspase-3 enzyme activity and Bax protein expression and lowering Bcl-2 protein expression	Chang et al. (2006)
Hepatocellular Carcinoma	HepG2 cells	Amygdalin	300 mg/mL	Arresting cell cycle at G2/M; Promoting cell apoptosis, heightening p53, Bax, cytochrome c and caspase-3 levels as well as diminishing Bcl-2 levels	El-Desouky et al. (2020)
Hepatocellular Carcinoma	HepG2 cells	Amygdalin	2.6 mg/mL	Delaying cell cycle at S and G2/M stages, inducing autophagy and apoptosis through inhibition of AMPK/mTOR and Bcl-2 pathway; Raising GSH levels and lessening MDA levels to alleviate cell necrosis caused by sorafenib	El-Sewedy et al. (2023)
Hepatocellular Carcinoma	DMBA-induced mice liver cancer	80% aqueous methanol of ASA; Amygdalin	ASA extract (400 mg/kg, oral administration) and amygdalin (1.85 mg/kg) once a day for 4 weeks	Up-regulating caspase-3 and downregulated Bcl-2 mRNA levels to inhibit apoptosis; Enhancing SOD, CAT, GSH, TAC levels and impeding MDA levels to exert antioxidant effects; Lowering beclin-1 mRNA level to regulate autophagy; Down-regulating the expressions of TNF-α, VEGF and PCNA to exert anti-inflammation, anti-angiogenesis and anti-proliferation effects, respectively	Hosny et al. (2021)
Non-small cell lung cancer	H1299/M and PA/M cells	Amygdalin	2.5 and 5 mg/mL	Impeding proliferation, invasion and migration by hampering integrin β1, integrin β4, ILK, FAK, p-FAK, β-catenin, Akt and RICTOR protein expressions while up-regulating the expression of E-cadherin	Qian et al. (2015)
Lung Cancer	A549 and PC9 cells; Xenografted mice model	Amygdalin	*In vitro*: 10, 20 and 30 mg/mL; *In vivo*: 40 and 80 mg/kg; three times a week for 2 weeks	Activation of NFκB-1/NFκB signaling pathway and to prompt mitochondria-mediated apoptosis	Lin et al. (2022)
Renal cell carcinoma	Caki-1, KTC-26 and A498 cells	Amygdalin	10 mg/mL	Diminishing adhesion to HUVECs, immobilized collagen and fibronectin; Impeding chemotaxis and invasion ability through regulating the protein expressions of integrin α and β, and affecting the total content of integrin	Juengel et al. (2016a)
Renal cell carcinoma	Caki-1, KTC-26 and A498 cells	Amygdalin	10 mg/mL	Prompting cell cycle arrest and inhibition of growth, lessening CDK, CDK2, CDK4, cyclin A, cyclin B and cyclin D protein expressions; Altering cell differentiation, enhancing E-cadherin but hampering N-cadherin level	Juengel et al. (2016b)
Bladder cancer	UMUC-3, TCCSUP and RT112 cells	Amygdalin	10 mg/mL	Hampering the adhesion of UMUC-3, RT112 and TCCSUP cells to vascular endothelium and immobilized collagen; Repressing UMUC - 3, RT112 but boosting the cell migration capacity of TCCSUP cell	Makarević et al. (2014b)
Bladder cancer	UMUC-3, TCCSUP and RT112 cells	Amygdalin	10 mg/mL	Induction of apoptosis; Delaying cell cycle and ‘arresting G0/G1 stage; Diminishing proliferation and growth by down-regulating the expression of CDK2 and cyclin A	Makarević et al. (2014a)
Cervical cancer	Hela cells; Xenografted nude mice	Amygdalin	*In vitro*:1.25, 2.5, 5, 10 and 20 mg/mL; *In vivo*: 300 mg/kg for 14 days	Hampering tumor growth; Induction of apoptosis by up-regulating Bax but down-regulating Bcl-2 protein expression, and intensifying caspase-3 enzyme activity	Chen et al. (2013)
Pancreatic cancer	PANC-1 cells	20% aqueous methanol of ASA; Amygdalin	ASA extracts:100–1,000 μg/mL; Amygdalin:5–40 mg/mL	Inhibition of cell growth, IC50 = 704 μg/mL at 72 h for ASA extracts and 35 mg/mL at 72 h for amygdalin; Activation of apoptotic through mitochondria-dependent pathway and enhancing mRNA level of caspase-3 and Bax/Bcl-2 mRNA expression ratio	Aamazadeh et al. (2020)
Acute leukemia	NALM-6 and KG-1 cells	Ethyl acetate extracts of ASA	IC50 = 0.388 mg/mL and 0.159 mg/mL for 48 h of NALM-6 and KG-1, respectively	Inducing apoptosis and up-regulating caspase-3 mRNA level	Mosadegh Manshadi et al. (2019)

#### 4.1.1 Breast carcinoma

Breast cancer is the most prevalent gynecological malignant tumor worldwide. The cure rate for patients diagnosed with early-stage breast cancer can reach 80%. However, treating patients in the advanced stages poses significant challenges (Zannetti, 2023). Conventional chemotherapy, radiotherapy, and targeted drug treatment are commonly used to treat breast cancer. Unfortunately, many patients develop drug resistance, experience cancer recurrence, and develop secondary diseases. *In vitro* studies, amygdalin, found in ASA, shows suppressive effects on various breast cancer cell lines including Hs578T, MCF-7, MDA-MB-231, SK-BR-3, and T47D cells, by inhibiting cancerous proliferation and migration, and inducing apoptosis, autophagy and oxidative stress.

Amygdalin impedes cell adhesion and migration by regulating integrin protein expression, which are cell adhesion molecules consisting of α and β subunits. Integrins facilitate the interaction between cancer cells and components of the extracellular matrix, thus influencing cell adhesion and eventually leading to cancer cell metastasis (Hoshino et al., 2015). In Hs578T breast cancer cells, amygdalin demonstrated a dose-dependent inhibition of cell adhesion, and it was observed that this inhibitory effect could potentially be attributed to the downregulation of integrin α5 protein expression (Lee and Moon, 2016). A decrease in mRNA levels of integrin αV/β3 and integrin α5 was observed in both MDA-MB-231 and MCF-7 cell lines, leading to the adhesion of cancer cells to fibronectin and collagen in the extracellular matrix. This decrease has an impact on the migration and metastasis of cancer cells. Notably, amygdalin shows a stronger inhibitory effect on integrin αV/β3 in MDA-MB-231 cells. Additionally, there were distinct variations in mRNA levels of integrin β1, β2, and β4 between the two cell lines. In MCF7 cells, integrin β1 and β4 levels increased, while integrin β2 levels decreased. Conversely, in MDA-MB-231 cells, the opposite trend was observed (Mosayyebi et al., 2021). The impact of amygdalin on cell adhesion and its effect on integrin protein expression have been extensively studied. However, the specific impact on different heterodimers is still not fully understood. A study conducted on MCF-7 cells showed that after 24 h and 48 h of amygdalin treatment, the IC50 values were determined to be 200.6 and 197 μg/mL, respectively. Additionally, Microarray Hybridization revealed that amygdalin can downregulate 19 out of 32 DNA replication-related genes, including MCM3, MCM6, MCM4, PCNA, and FEN1. This suggests that amygdalin may inhibit the proliferation of breast cancer cells by affecting DNA replication (Albogami and Alnefaie, 2021).

Apoptosis has long been recognized as a significant mechanism for preventing tumor development. The inhibitory effect of apoptosis is determined by the expression of Bcl-2 and Bax proteins (Czabotar et al., 2014). Studies have shown that amygdalin, at concentrations of 10 and 20 mg/mL, effectively suppresses the expression of Bcl-2 protein and enhances the expression of Bax in SK-BR-3 and MCF-7 cell lines (Moradipoodeh et al., 2020). This indicates that amygdalin can inhibit apoptosis in breast cancer cells. The human epidermal receptor 2 (HER2) is closely associated with breast cancer development and apoptosis (Shi et al., 2022). Molecular docking studies have revealed that amygdalin forms hydrogen bonds and hydrophobic interactions with Bcl-2 and the active site amino acids of HER2 in HER2-overexpressing SK-BR-3 cells. However, the binding ability of amygdalin to the active site amino acids of HER2 is weaker compared to lapatinib, a HER2 tyrosine kinase inhibitor. The metabolites of amygdalin, such as benzaldehyde, mandelonitrile, and cyanide, also bind to Bcl-2, although their binding affinity is weaker compared to amygdalin (Moradipoodeh et al., 2019). Another study found that amygdalin can diminish the apoptosis of Hs578T breast cancer cells by activating the p38 MAPK signaling pathway and regulating the expression of Bcl-2 family and Caspase family proteins (Lee and Moon, 2016). Furthermore, when MCF-7 breast cancer cells and MCF-10A normal cells were treated with 50 μM amygdalin and 1 mg/mL ASA extract, it was observed that the activities of proteasomes 20S and 26S, Cathepsin B, and cathepsin L in MCF-7 cells were inhibited. Additionally, the expressions of p53, p27, and Bax were increased, indicating that amygdalin and ASA extract may promote apoptosis and regulate the autophagy cascade (Cecarini et al., 2022). Moreover, amygdalin can induce oxidative stress in breast cancer cells by increasing GSH activity and reducing MDA and oxidized glutathione levels, thereby exerting anti-cancer effects (Abboud et al., 2019).

#### 4.1.2 Prostatic cancer

Prostatic cancer is the most common type of cancer in men, with approximately 40% of patients eventually developing other metastatic diseases. Therefore, it is crucial to investigate the potential of natural chemical components found in plants as alternative therapies for prostate cancer treatment (Martínez-Piñeiro et al., 2003). Amygdalin has demonstrated anti-prostate cancer activity in LNCaP, DU-145, and PC3 cells. Its primary mechanisms involve inhibiting cell adhesion, migration and metastasis, and inducing apoptosis and cell cycle arrest, attributed to its downregulation of integrin α6 and Bcl-2, while upregulation of integrin α2, Bax and caspase-3, as well as inhibition of CDK1-cyclin B axis and the AKT-mTOR pathway.

A study demonstrated that treating DU-145 prostate cancer cells with 10 mg/mL amygdalin for 24 h inhibited their adhesion, chemotaxis, and migration. This inhibition was attributed to the downregulation of integrin α2 and the upregulation of α6. Integrin α2 plays a critical role in cell adhesion, which in turn regulates cell invasion and metastasis. However, a decrease in adhesion of PC3 cells was observed only after 2 weeks of amygdalin treatment, with no impact on their chemotaxis and migration abilities. Further experiments involving the knockout of integrins α2, α6, and β1 revealed distinct changes in the adhesion, chemotaxis, and migration abilities of DU-145 and PC3 cells (Mani et al., 2020). In conclusion, the effects of amygdalin on cell adhesion, migration, and metastasis are influenced by the epigenetics of tumor cells, and each cell line may have a specific set of receptors. Amygdalin has shown potential anticancer activities by influencing the cell cycle. In a 2-week study, amygdalin administration resulted in the prolongation of the G0/G1 phase and the shortening of the S phase and G2/M phase in LNCaP, DU-145, and PC3 cells. Additionally, it inhibited the expression of cell cycle regulatory proteins, including CDK1, CDK2, CDK4, cyclin A, cyclin B and cyclin D3, as well as the AKT-mTOR signaling cascade (Makarević et al., 2016). Furthermore, amygdalin has been found to enhance cell apoptosis by increasing caspase-3 enzyme activity and Bax protein expression, while decreasing Bcl-2 protein expression (Chang et al., 2006).

#### 4.1.3 Hepatocellular carcinoma

Hepatocellular carcinoma is a prevalent type of cancer. A study involving 148 hepatocellular carcinoma patients found that 75 of them died within 22 months. Cirrhosis developed in 77% of the patients, and the 1-year and 3-year survival rates were 70.8% and 47.6% respectively (Wongjarupong et al., 2021). After administering ASA treatment, there was a significant increase in the proportion of early apoptosis, late apoptosis, and necrosis cells in HepG2 hepatocellular carcinoma. This effect was positively correlated with the upregulation of p53, Caspase-3, and Bcl-2 activities, as well as the downregulation of Bax. It is worth noting that the pro-apoptotic effect of amygdalin is enhanced with the addition of zinc (El-Desouky et al., 2020). Sorafenib, a commonly used targeted drug for liver cancer treatment, often leads to severe side effects and drug resistance in patients (Zheng et al., 2014). Experiments have demonstrated that 2.6 mg/mL amygdalin alone or in combination with sorafenib can induce cell cycle arrest in HepG2 cells and trigger autophagy and apoptosis. These results align with the upregulation of AMPK, HMGB1, beclin-1, and ATG5 mRNA levels, as well as the downregulation of mTOR and Bcl-2 levels. Unlike sorafenib, amygdalin can increase GSH level, reduce MDA level, and exhibit strong DPPH free radical scavenging ability (El-Sewedy et al., 2023). These findings suggest that amygdalin holds significant potential for the treatment of hepatocellular carcinoma.

The therapeutic effects of ASA extract on liver cancer have been demonstrated *in vivo*. When liver cancer is induced by 2,2′-Bis (hydroxymethyl)butyric (DMBA), ASA methanol-water extract and amygdalin have been shown to significantly increase the levels of SOD, CAT, GSH, and TAC, while inhibiting MDA levels. These effects contribute to the anti-oxidant properties of ASA, which are crucial in protecting the liver from oxidative damage. Additionally, ASA has been found to downregulate the mRNA levels of Bcl-2 and beclin-1, reduce TNF-α and VEGF contents, and downregulate PCNA protein expression in mouse liver tissues (Hosny et al., 2021). These findings indicate that ASA can inhibit inflammation through apoptosis, autophagy, angiogenesis, and proliferation pathways, thereby exerting anti-cancer effects.

#### 4.1.4 Lung cancer

Lung cancer is a prevalent and deadly malignant tumor that often metastasizes to various organs including the brain, bone, liver, and kidney. Current treatments primarily focus on primary lung cancer, leading to a poor prognosis for metastatic patients (Yin et al., 2021). However, in highly metastatic non-small cell lung cancer cell lines H1299/M and PA/M, amygdalin at concentrations of 2.5 and 5 mg/mL significantly inhibits cell proliferation, migration, and invasion. The inhibition rates of cell proliferation decreased by 15.6% and 25.1% respectively under these concentrations. Amygdalin achieves its function by reducing the levels of integrin β1 and β4, while upregulating the level of E-cadherin (Qian et al., 2015). This not only affects tumor cell adhesion but also activates FAK, β-catenin, and the downstream AKT-mTOR signaling pathway to mediate cell proliferation, adhesion, and metastasis. Additionally, amygdalin effectively promotes cancer cell apoptosis in A549 and PC9 cancer cells *in vitro*, as well as in A549 cell xenograft mice. This is achieved by inhibiting the NF-κB signaling pathway through increased protein expression of NF-κB-1 and further altering the expression of apoptosis-related proteins Bax, Bcl-2, cytochrome C, caspase 9, caspase 3, and PARP (Lin et al., 2022). In conclusion, amygdalin shows promising potential for treating lung cancer and may serve as a potential NF-κB-1 agonist.

#### 4.1.5 Renal cell carcinoma

Renal cell carcinoma, which accounts for 80% of all kidney cancers, is a common type of urinary tract tumor. In the United States, there are approximately 64,000 new cases and 14,000 deaths associated with renal cell carcinoma each year (Singh, 2021). Amygdalin has demonstrated anti-renal cell carcinoma activity *in vitro*, specifically in Caki-1, KTC-26, and A498 cells. This activity is attributed to the regulation of integrin α and β protein expressions, leading to the inhibition of adhesion and migration. Additionally, amygdalin inhibits CDK/cyclin complexes, thereby arresting the cell cycle.

Amygdalin at a concentration of 10 mg/mL has been found to inhibit the adhesion, chemotaxis and migration of Caki-1, KTC-26 and A498 cells, due to the downregulation of integrins α5 and α6 levels. Furthermore, the expression changes of other integrin subtypes in these cells vary, suggesting that the integrin profile may be specific to each cell line (Juengel et al., 2016a). Additionally, amygdalin induces cell cycle arrest by increasing the number of cells in the G0/G1 phase of Caki-1 and A498 cells, and in the S phase of KTC-26 cells, which may be attributed to the diminishment of CDK, CDK2, CDK4, cyclin A, cyclin B and cyclin D protein expressions (Juengel et al., 2016b). Notably, amygdalin may impact cancer cell differentiation by regulating N-cadherin and E-cadherin, potentially influencing the prognosis of the cancer. However, further research is necessary to investigate the specific impact of cadherin on cell differentiation in renal cell carcinoma.

#### 4.1.6 Bladder cancer

Bladder cancer is a prevalent form of cancer that affects the urinary system, leading to significant morbidity and mortality. A key symptom of bladder cancer is painless hematuria. As the disease progresses, patients may experience urinary retention, poor urination, and urinary tract obstruction (Xiang et al., 2021). In recent studies, amygdalin has shown promise in inhibiting the adhesion of bladder cancer cells (UMUC-3, TCCSUP, and RT112) by potentially affecting integrin expression. However, the specific integrin profile in different cell lines appears to play a more significant role. Furthermore, amygdalin has been observed to impede the migration of UMUC-3 and RT112 cells, while paradoxically increasing the migration of TCCSUP cells (Makarević et al., 2014b). It is important to note that although amygdalin can inhibit cancer cell adhesion, prolonged exposure to certain cancer cells may promote the migration of non-adherent cells. Additionally, amygdalin has demonstrated inhibitory effects on the growth and proliferation of UMUC-3, TCCSUP, and RT112 cancer cells. This is primarily achieved by causing cell cycle delay and arresting cells in the G0/G1 phase, possibly through the downregulation of CDK2 and cyclin A protein expression (Makarević et al., 2014a).

#### 4.1.7 Other cancers

In addition to its therapeutic potential for the above cancer types, ASA has also shown suppression of cervical cancer, pancreatic cancer and blood cancer, mainly based on the effects of amygdalin. Both *in vivo* and *in vitro* studies have demonstrated that amygdalin has positive therapeutic effects on cervical cancer. The main mechanism of amygdalin’s therapeutic effect is inhibition of cell growth and promotion of apoptosis (Chen et al., 2013). Furthermore, research has shown that the methanol aqueous extract of ASA and amygdalin can promote apoptosis in PANC-1 pancreatic cancer cells (Aamazadeh et al., 2020). Additionally, a separate study found that the ethyl acetate extract of ASA has an inhibitory effect on NALM-6 acute B lymphoid leukemia cells and KG-1 myeloid leukemia cells (Mosadegh Manshadi et al., 2019).

### 4.2 Anti-oxidation

The anti-oxidant activity of ASA primarily involves the elimination of lipid peroxidation, reduction of reactive oxygen species (ROS) accumulation, and enhancement of anti-oxidant enzyme activity, and the main functional substances are polyphenols (Table 3). Malondialdehyde (MDA) is a crucial marker for LPO resulting from the oxidation of polyunsaturated fatty acids (Liu et al., 2018). A study with the ethanol-induced rat liver injury and oxidative stress model has demonstrated that consumption of ASA significantly decreases LDH content in serum, MDA level in red blood cells, brain, kidney and heart of rats while increasing the content of anti-oxidant enzymes such as superoxide dismutase (SOD) and glutathione S-transferase (GST) in the liver (Yurt and Celik, 2011). This indicates that ASA can prevent liver injury by increasing the activity of anti-oxidant enzymes and inhibiting lipid peroxides to resist oxidative stress. *Mahboub, H.H. et al.* have also reported that ASA consumption significantly enhances the overall anti-oxidant capacity within *cyprinus carpio*, which may be attributed to the upregulation of anti-oxidant enzymes. When 10 g/kg ASA was added to the basic diet for continuous feeding over a period of 60 days, the total anti-oxidant capacity (TAC), glutathione (GSH), and SOD contents in liver tissue were increased from 16.66 ng/mg to 58.33 ng/mg, 30.33 mmol/g to 66.33 mmol/g, and 14 to 48 U/mg respectively, meanwhile, SOD, GPX, and GSS mRNA levels in spleen were also intensified (Mahboub et al., 2022).

**TABLE 3:** Anti-oxidant activity of ASA.

Extract/compound	Mechanism	Minimal active concentration/dose	*In vitro*/*In vivo*	Reference
ASA ethanol extract	Decreasing LDH content in serum and MDA accumulation in erythrocyte, brain, kidney, and heart, while heightening SOD and GST content in liver in ethanol-induced rats liver injury and oxidative stress model	15% ASA +20% alcohol-water	*In vivo*	Yurt and Celik (2011)
ASA	Raising TAC, SOD, and GSH content in liver tissue of *Cyprinus carpio*, while up-regulating SOD, GPX, and GSS mRNA levels in spleen	2.5, 5, and 10 g/kg	*In vivo*	Mahboub et al. (2022)
ASA polyphenols	Reducing ferric, Scavenging ABTS radicals, hydrogen peroxide radicals, DPPH radicals, hydroxy radicals, and peroxy radicals	IC50 = 3.05, 0.24, 18.71, 13.77, 37.64, and 32.46 mg/mL, respectively	*In vitro*	Qin et al. (2019)
ASA polyphenols	Scavenging DPPH radicals	100 and 300 μg/mL, respectively	*In vitro*	Yiğit et al. (2009)
ASA oil	Reducing ferric	IC50 = 1.07–1.38 mM Fe2^+^/L in 5 different ASA varieties	*In vitro*	Stryjecka et al. (2019)
ASA n-hexane extract	Reducing ferric, intensifying TAC, scavenging DPPH radicals, and hydrogen peroxide radicals	IC50 = 163.35, 110.80, 98.61, and 516.63 μg/mL, respectively	*In vitro*	Tareen et al. (2021)
A neutral polysaccharide (AP-1)	Scavenging DPPH radicals, ABTS radicals, and hydroxyl radicals	IC50 = 2.95, 0.522, and 0.053 mg/mL, respectively	*In vitro*	Peng et al. (2023)
Amygdalin	Inhibiting the production of ROS in RAW264.7 cells, while elevating the content of CAT and SOD	10 and 40 μM	*In vitro*	Trang et al. (2022)

In addition, the anti-oxidant capacity of ASA is positively correlated with the total phenolic content in the extract. Phenolic compounds have the ability to scavenge free radicals and participate in redox reactions to protect cells from oxidative damage (Desmarchelier et al., 2005). Qin, F. et al. extracted ASA with 50% ethanol and found that the extract had a total phenolic content of 874.49 ± 6.75 mg GAE (gallic acid equivalent)/100 g fresh weight. This extract demonstrated excellent free radical scavenging ability in free radical scavenging assays. The extract showed significantly stronger total reducing activity, 2′-Azinobis-(3-ethylbenzthiazoline-6-sulphonate) (ABTS) free radical scavenging activity, and H_2_O_2_ scavenging activity compared to ascorbic acid. However, its 2,2-diphenyl-1-picrylhydrazyl (DPPH) free radical scavenging ability, hydroxide ion, and peroxy ion were comparable to that of ascorbic acid (Qin et al., 2019). However, Yiğit, D. et al. extracted ASA with methanol and water, the total phenolic content was 0.4 and 0.5 μg GAE/mL, respectively, while the DPPH free radical scavenging activity was poor at the concentration of 100–300 μg/mL, indicating that the total phenolic content of ASA is a key factor affecting its anti-oxidant capacity (Yiğit et al., 2009). Furthermore, the variety and origin of ASA also play important roles in determining the total phenolic content. Among the five varieties of ASA in Poland, the “Somo” variety had the highest total phenolic content of 1.22 mM GAE/L, and this variety showed the best anti-oxidant activity according to the ferric reducing anti-oxidant power (FRAP) test. Similarly, ASA from five different regions of Pakistan exhibited significant differences in anti-oxidant activity after extraction with n-hexane. The ASA from Badoghur had a total phenol content of 5,005 mg GAE/100 g dry weight, which was significantly higher than that of other origins. Additionally, ASA from Badoghur showed the smallest half maximal inhibitory concentration (IC50) value in total anti-oxidant capability, hydrogen peroxide scavenging, DPPH, and FRAP experiments, indicating the strongest anti-oxidant activity (Tareen et al., 2021).

Moreover, recent studies have revealed that ASA contains other components, besides phenols, that have anti-oxidant capacity. One such component is a neutral polysaccharide called AP-1, which was extracted and isolated from ASA. AP-1 exhibited a maximum inhibition rate of 87.74% for DPPH radical scavenging activity at a concentration of 10 mg/mL, which is slightly lower than that of vitamin C. However, ABTS assay revealed that AP-1 has comparable free radical scavenging ability and hydroxyl radicals to vitamin C (Peng et al., 2023). Furthermore, amygdalin also demonstrated anti-oxidant capacity by inhibiting ROS accumulation and activating anti-oxidant enzyme activities such as catalase (CAT) and SOD in RAW264.7 cells (Trang et al., 2022).

### 4.3 Antimicrobial activity

A growing number of experimental studies have demonstrated the broad spectrum of antibacterial activity exhibited by ASA. Different extracts of ASA have varying degrees of antibacterial activity, as outlined in Table 4. Among these extracts, ASA volatile oil stands out for its extensive antibacterial activity, which is likely attributed to its main component, benzaldehyde. This component has been widely utilized in cosmetics due to its antibacterial, antiseptic, and stabilizing effects (Rodrigues and de Carvalho, 2022). ASA volatile oil exhibits excellent antibacterial activity against Gram-positive bacteria such as *Staphylococcus aureus*, *Staphylococcus epidermidis* and *methicillin-resistant S. aureus* as well as Gram-negative bacteria including *Escherichia coli*, *Pseudomonas aeruginosa*, *P. aeruginosa D24*, *Salmonella typhimurium* and *Shigella sonnei*. Complete growth inhibition was observed with a minimum inhibitory concentration (MIC) ranging from 250 to 500 μg/mL. Furthermore, the ASA essential oil also displayed certain antibacterial activity against several other clinical pathogenic bacteria (Lee et al., 2014). Additionally, ASA volatile oil exhibited a significant inhibitory effect on *Listeria monocytogenes* in solid medium, micro-atmospheric medium, liquid medium and beef slices (Wang et al., 2020). *Listeria monocytogenes* is an intracellular parasite, primarily transmitted through food, and severe poisoning can result in blood and brain infections (Stevens et al., 2006). Studies have indicated that polyphenols can bind to bacterial cell membrane, disrupt bacterial cell membrane proteins, induce bacterial metabolic disorders, and ultimately inhibit bacterial growth or kill bacteria (Messaoudene et al., 2022). ASA is rich in polyphenols, which exhibit significant antibacterial activity against both Gram-negative bacteria (*E. coli* and *Acetobacter aceti*) and Gram-positive bacteria (*S. aureus*, *Bacillus subtilis* and *Bacillus cereus*). The inhibitory zone ranges from 13.0 to 18.6 mm and MIC between 31.25 and 250 μg/mL (Qin et al., 2019). However, ASA demonstrates a stronger antibacterial effect against Gram-positive bacteria. This could be attributed to the outer membrane permeability barrier of Gram-negative bacteria cell wall, which limits the interaction between antibacterial agents and their targets within bacterial cells. Moreover, both aqueous and alcoholic extracts of ASA display significant antibacterial activity against *E. coli* and *S. aureus* with inhibitory diameters ranging from 13 to 15 mm and MIC values of 0.312–0.625 mg/mL (Yiğit et al., 2009). However, the ASA base oil exhibits poor antibacterial activity, consistent with previous findings that the fatty acids in ASA lack antibacterial properties (Moola et al., 2022).

**TABLE 4:** Antimicrobial activity of ASA.

Pathogenic microorganism	Extract/compound	*In vitro*/*In vivo*	Minimal active concentration/dose	Mechanisms	Reference
*Bacillus cereus*	Volatile oil	*In vitro*	MIC = 2,000 μg/mL	Moderate inhibition of growth	Lee et al. (2014)
*Enterococcus faecalis*	Volatile oil	*In vitro*	MIC = 4,000 μg/mL	Weak inhibition of growth	Lee et al. (2014)
*Methicillin-resistant*	Volatile oil	*In vitro*	MIC = 500 μg/mL	Complete inhibition of growth	Lee et al. (2014)
*S.aureus (MRSA) P15*					
*Staphylococcus aureus*	Volatile oil	*In vitro*	MIC = 500 μg/mL	Complete inhibition of growth	Lee et al. (2014)
*Staphylococcus epidermidis*	Volatile oil	*In vitro*	MIC = 250 μg/mL	Complete inhibition of growth	Lee et al. (2014)
*Citrobacter freundii*	Volatile oil	*In vitro*	MIC = 2,000 μg/mL	Moderate inhibition of growth	Lee et al. (2014)
*Enterobacter aerogenes*	Volatile oil	*In vitro*	MIC = 2000 μg/mL	Moderate inhibition of growth	Lee et al. (2014)
*Enterobacter cloacae*	Volatile oil	*In vitro*	MIC = 1,000 μg/mL	Moderate inhibition of growth	Lee et al. (2014)
*Escherichia coli*	Volatile oil	*In vitro*	MIC = 500 μg/mL	Complete inhibition of growth	Lee et al. (2014)
*Klebsiella pneumoniae*	Volatile oil	*In vitro*	MIC = 2,000 μg/mL	Moderate inhibition of growth	Lee et al. (2014)
*Proteus mirabilis*	Volatile oil	*In vitro*	MIC = 2,000 μg/mL	Moderate inhibition of growth	Lee et al. (2014)
*Pseudomonas aeruginosa*	Volatile oil	*In vitro*	MIC = 500 μg/mL	Complete inhibition of growth	Lee et al. (2014)
*P.aeruginosa D24*	Volatile oil	*In vitro*	MIC = 500 μg/mL	Complete inhibition of growth	Lee et al. (2014)
*Salmonella typhimurium*	Volatile oil	*In vitro*	MIC = 500 μg/mL	Complete inhibition of growth	Lee et al. (2014)
*Serratia marcescens*	Volatile oil	*In vitro*	MIC = 1,000 μg/mL	Moderate inhibition of growth	Lee et al. (2014)
*Shigella sonnei*	Volatile oil	*In vitro*	MIC = 500 μg/mL	Complete inhibition of growth	Lee et al. (2014)
*Candida albicans*	Volatile oil	*In vitro*	MIC = 1,000 μg/mL	Complete inhibition of growth	Lee et al. (2014)
*Malassezia furfur*	Volatile oil	*In vitro*	MIC = 250 μg/mL	Complete inhibition of growth	Lee et al. (2014)
*Listeria monocytogenes*	Volatile oil	*In vitro*	0.5% and 1%	Displaying antimicrobial effects in solid medium, micro-atmosphere, liquid media and sliced beef	Wang et al. (2020)
*Escherichia coli*	Polyphenols	*In vitro*	MIC = 250 μg/mL	Great antimicrobial potency	Qin et al. (2019)
*Staphylococcus aureus*	Polyphenols	*In vitro*	MIC = 125 μg/mL	Great antimicrobial potency	Qin et al. (2019)
*Bacillus subtilis*	Polyphenols	*In vitro*	MIC = 31.25 μg/mL	Great antimicrobial potency	Qin et al. (2019)
*Bacillus cereus*	Polyphenols	*In vitro*	MIC = 250 μg/mL	Great antimicrobial potency	Qin et al. (2019)
*Aspergillus niger*	Polyphenols	*In vitro*	--	No antimicrobial potency	Qin et al. (2019)
*Acetobacter aceti*	Polyphenols	*In vitro*	MIC = 62.5 μg/mL	Great antimicrobial potency	Qin et al. (2019)
*Escherichia coli*	Methanol extract and water extract	*In vitro*	MIC = 0.312, 0.625 mg/mL, respectively	Significant antibacterial activity	Yiğit et al. (2009)
*Proteus mirabilis*	Methanol extract and water extract	*In vitro*	MIC = 0.625 mg/mL for water extract	Significant antibacterial activity	Yiğit et al. (2009)
*Staphylococcus aureus*	Methanol extract and water extract	*In vitro*	MIC = 0.312 mg/mL	Significant antibacterial activity	Yiğit et al. (2009)
*Candida albicans*	Methanol extract and water extract	*In vitro*	MIC = 0.625, 2.5 mg/mL, respectively	Moderate antibacterial activity	Yiğit et al. (2009)
*Candida glabrata*	Methanol extract and water extract	*In vitro*	MIC = 1.25 mg/mL for methanol extract	Moderate antibacterial activity	Yiğit et al. (2009)
*Candida parapisilosis*	Methanol extract and water extract	*In vitro*	MIC = 2.5 mg/mL	Moderate antibacterial activity	Yiğit et al. (2009)
*Enterococcus faecium*	Carrier oil	*In vitro*	MIC = 4 mg/mL	Poor antimicrobial activity	Moola et al. (2022)
*Staphylococcus aureus*	Carrier oil	*In vitro*	MIC = 3 mg/mL	Poor antimicrobial activity	Moola et al. (2022)
*Klebsiella pneumoniae*	Carrier oil	*In vitro*	MIC = 2 mg/mL	Poor antimicrobial activity	Moola et al. (2022)
*Acinetobacter baumannii*	Carrier oil	*In vitro*	MIC = 4 mg/mL	Poor antimicrobial activity	Moola et al. (2022)
*Pseudomonas aeruginosa*	Carrier oil	*In vitro*	MIC = 3.33 mg/mL	Poor antimicrobial activity	Moola et al. (2022)
*Escherichia coli*	Carrier oil	*In vitro*	MIC = 3.5 mg/mL	Poor antimicrobial activity	Moola et al. (2022)
*Candida albicans*	Carrier oil	*In vitro*	MIC = 1 mg/mL	Moderate antimicrobial activity	Moola et al. (2022)
*Aeromonas veronii*	ASA powder	*In vivo*	2.5, 5 and 10 g/kg	Dose-dependently lowering mortality rate	Mahboub et al. (2022)
*Corynebacterium xerosis*	Ethanol extract	*In vitro*	62.5, 125 ppm	Did not exhibit a bactericidal effect	Mikoshiba et al. (2006)
*Microsporum canis*	Volatile oil	*In vitro*	0.5–4 μL/mL	Completely mycelial growth inhibition	Ibrahim and Abd El-Salam (2015)
*Epidermophyton floccosum*	Volatile oil	*In vitro*	0.5–4 μL/mL	Completely mycelial growth inhibition	Ibrahim and Abd El-Salam (2015)
*Trichophyton rubrum*	Volatile oil	*In vitro*	0.5–4 μL/mL	Completely mycelial growth inhibition	Ibrahim and Abd El-Salam (2015)
*Trichophyton mentagrophytes*	Volatile oil	*In vitro*	0.5–4 μL/mL	Completely mycelial growth inhibition	Ibrahim and Abd El-Salam (2015)
*Fusarium oxysporum* sp. *cucumebrium* Owen	Volatile oil	*In vitro*	EC50 = 511.7 μg/mL	Antifungal Activity	Geng et al. (2016)
*Valsa mali* Miyabe et Yamade	Volatile oil	*In vitro*	EC50 = 610.8 μg/mL	Antifungal Activity	Geng et al. (2016)
*Pyricularia oryzae cavgra*	Volatile oil	*In vitro*	EC50 = 429.3 μg/mL	Antifungal Activity	Geng et al. (2016)
*Fusarium graminearum*	Volatile oil	*In vitro*	EC50 = 627.9 μg/mL	Antifungal Activity	Geng et al. (2016)
*Alternaria alternata* (Fr) Keissler	Volatile oil	*In vitro*	EC50 = 642.0 μg/mL	Antifungal Activity	Geng et al. (2016)
*Alternaria solani*	Volatile oil	*In vitro*	EC50 = 103.2 μg/mL	Antifungal Activity	Geng et al. (2016)
*Phytophthora capsici* Leonian	Volatile oil	*In vitro*	EC50 = 600.5 μg/mL	Antifungal Activity	Geng et al. (2016)
*Gloeosporium fructigenum*	Volatile oil	*In vitro*	EC50 = 225.9 μg/mL	Antifungal Activity	Geng et al. (2016)
*Fusarium oxysporum* f. sp. *lycopersici* Synder et Hansen	Volatile oil	*In vitro*	EC50 = 295.1 μg/mL	Antifungal Activity	Geng et al. (2016)
*Gloeosporium orbiculare*	Volatile oil	*In vitro*	EC50 = 273.7 μg/mL	Antifungal Activity	Geng et al. (2016)
*Verticillium dahliae* Kleb	Volatile oil	*In vitro*	EC50 = 325.2 μg/mL	Antifungal Activity	Geng et al. (2016)
*Gaeumannomyces graminis* var. *tritici*	Volatile oil	*In vitro*	EC50 = 192.0 μg/mL	Antifungal Activity	Geng et al. (2016)
*Botrytis cinerea*	Volatile oil	*In vitro*	EC50 = 217.0 μg/mL	Antifungal Activity	Geng et al. (2016)
*Fusarium oxysporum* f. sp. *vasinfectum*	Volatile oil	*In vitro*	EC50 = 526.7 μg/mL	Antifungal Activity	Geng et al. (2016)
*Curvularia lunata*	Volatile oil	*In vitro*	EC50 = 509.5 μg/mL	Antifungal Activity	Geng et al. (2016)
*Fusarium oxysporum* (Schlecht.)	Volatile oil	*In vitro*	EC50 = 423.8 μg/mL	Antifungal Activity	Geng et al. (2016)
*Colletotrichum gloeosporioides* (Penz.) et Sacc	Volatile oil	*In vitro*	EC50 = 381.8 μg/mL	Antifungal Activity	Geng et al. (2016)
*Fusarium oxysporum* f. sp. *niveum*	Volatile oil	*In vitro*	EC50 = 569.3 μg/mL	Antifungal Activity	Geng et al. (2016)
*Alternaria brassicae*	Volatile oil	*In vitro*	EC50 = 50.2 μg/mL	Antifungal Activity	Geng et al. (2016)
*Gloeosporium orbiculare*	Volatile oil	*In vivo*	4–12 mg/mL	High protective and therapeutic effects	Geng et al. (2016)
*Blumeria graminis*	Volatile oil	*In vivo*	4–12 mg/mL	Medium protective effect and weak therapeutic effect	Geng et al. (2016)

Millions of people worldwide are affected by superficial fungal infections, the most common skin disease caused by dermatophytes that parasitize on the surface layer of the stratum corneum. *Microsporum canis* and *Microsporum* are often implicated in these infections. The clinical symptoms of dermatophytosis are generally mild, and active lesions typically heal within 6–8 weeks. ASA volatile oil has demonstrated significant antibacterial activity against keratinophilic fungi, completely inhibiting their growth at a concentration of 100 μg/mL (Ibrahim and Abd El-Salam, 2015). In addition, among various ASA extracts, volatile oil exhibited notable inhibitory effects on *Malassezia furfur* and *Candida albicans*, with MIC of 250 and 1,000 μg/mL (Lee et al., 2014), respectively. However, ASA polyphenols only showed moderate inhibition against *candida*, while base oil displayed poor inhibitory activity (Yiğit et al., 2009; Moola et al., 2022). Furthermore, ASA volatile oil exhibited inhibitory effect on 19 plant pathogenic fungi, suggesting its potential as a plant and agricultural fungicide (Geng et al., 2016).

ASA is known to contain antibacterial substances such as volatile oil and polyphenols, which contribute to its excellent antibacterial potential. While there have been numerous studies on the antibacterial activity of ASA, few have explored its underlying mechanism. Mahboub, H.H. et al. suggested that the antibacterial effect of ASA might be attributed to immune enhancement (Mahboub et al., 2022), while Mikoshiba, S. et al. proposed that metabolism could play a vital role (Mikoshiba et al., 2006). However, these studies are still limited, and further research is necessary to fully understand the antibacterial mechanism of ASA.

### 4.4 Anti-inflammation

**FIGURE 5:**
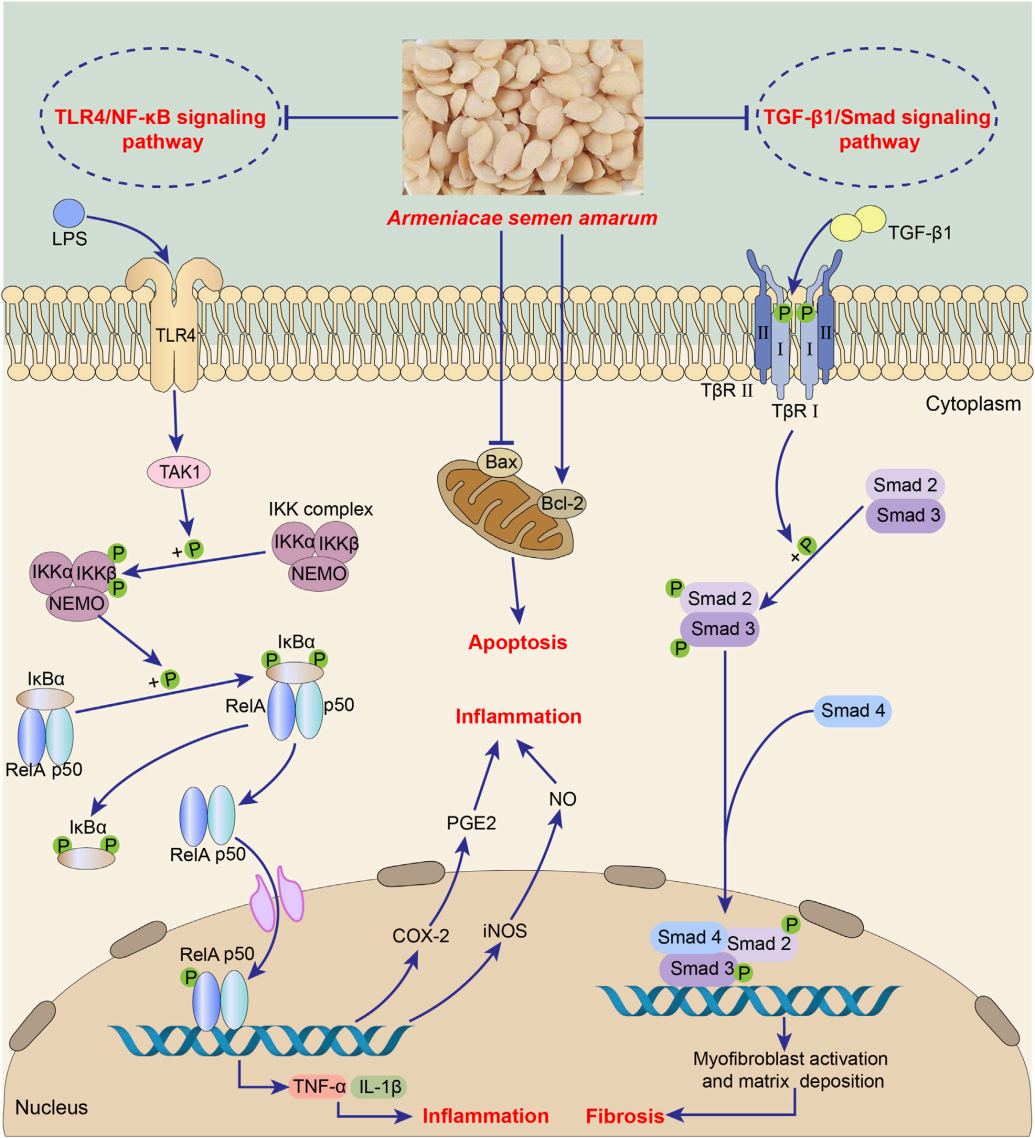
Anti-inflammation of *Armeniacae semen amarum*.

The main substance exerting anti-inflammation effect in ASA may be amygdalin, which can inhibit the abnormal activation of TGF-β1/Smad signaling pathway and TLR4/NF-κB signaling pathway (Figure 5, Table 5). It was found that intraperitoneal injection of 4 mg/kg amygdalin significantly alleviate bleomycin-induced neutrophil inflammatory infiltration in mouse lung tissues and reduced the number of macrophages and neutrophils in BALF, which are precursors of immune defense. The underlying mechanism may be the inhibition of TGF-β1/Smad signaling pathway (Jiao et al., 2023). In addition, amygdalin can directly hamper the expression of cytokines to exert anti-inflammatory effect. In the model of intraplantar injection of formalin, 1 mg/kg amygdalin significantly inhibited TNF-α and IL-1β mRNA levels in rat paw skins, which was comparable to that of indomethacin (Hwang et al., 2008). Besides, amygdalin can regulate the expression of inflammation-related enzymes and play an indirect anti-inflammatory role. Cyclooxygenase-2 (COX-2) and inducible nitric oxide synthase (iNOS) are involved in the inflammatory response and induce the production of inflammatory mediators prostaglandin E2 (PGE2) and NO, respectively (Chang et al., 2005). In LPS-stimulated BV2 cell model, treatment with 10 or 100 μg/mL amygdalin and 0.1 or 1 mg/mL ASA aqueous extract can significantly downregulate COX-2 and iNOS mRNA levels, and the contents of PGE2 and NO (Chang et al., 2005; Yang et al., 2007). Furthermore, in a model of HUVEC injury induced by PM2.5, amygdalin at concentrations of 2.5, 5, and 10 μg/mL has been shown to diminish the levels of COX-2, IL-6, TNF-α, and IL-1β, while promoting apoptosis of damaged cells via impeding aberrant activation of TLR4/NF-κB signaling pathway (Wang et al., 2022). Moreover, it has been discovered that oral administration of 15 mg/kg amygdalin can restore Th1/Th2 immune imbalance to alleviate airway inflammation in an ovalbumin-induced asthma mice model (Cui et al., 2023). However, further studies are needed to determine whether other components of ASA have anti-oxidant effects.

**TABLE 5:** Anti-inflammation, cardiovascular protection, neuroprotection, respiratory and digestive system protection, antidiabetic, liver and kidney protection and other pharmacological activities of ASA.

Extract/compound	Cell line/model	*In vitro*/*In vivo*	Dose	Mechanisms	Reference
Anti-inflammation					
Amygdalin	BLM-induced mice lung fibrosis model	*In vivo*	4 mg/kg, i.p.	Decreasing lung index, diminishing macrophages, neutrophils, and IL-2 levels in BALF, hampering TGF-β1, Smad2, Smad3, phospho-Smad2, and phospho-Smad3 protein expressions	Jiao et al. (2023)
Amygdalin	Formalin-induced mice model	*In vivo*	0.1, 0.5, 1, and 10 mg/kg, i.v.	Down-regulating TNF-α and IL-1β mRNA levels	Hwang et al. (2008)
ASA aqueous extract	LPS stimulated BV-2 cell inflammation	*In vitro*	0.01, 0.1, and 1 mg/mL	Inhibiting COX-1, COX-2, iNOS mRNA levels and protein expressions to impede the production of PGE2 and NO	Chang et al. (2005)
Amygdalin	LPS-induced BV-2 cell inflammation	*In vitro*	10 and 100 μg/mL	Reducing COX-1, COX-2 and iNOS mRNA levels and protein expressions to hamper the accumulation of PGE2 and NO	Yang et al. (2007)
Amygdalin	PM2.5-induced HUVEC injury	*In vitro*	2.5, 5, and 10 μg/mL	Decreasing the productions and mRNA levels of IL-6, TNF-α, and IL-1β, Lessening COX-2 mRNA level and protein expression, Diminishing TLR4, phospho-NF-κB p65, NF-κB p50, phospho-IκBα, and Bax protein expressions, while strengthening Bcl-2 protein expression	Wang et al. (2022)
Amygdalin	Ovalbumin-induced asthma mice model	*In vivo*	15 mg/kg, i.g	Alleviating airway inflammation, reducing macrophages, eosinophils, neutrophils, lymphocytes, and total cells in BALF, depressing IL-4, IL-5, and IL-13 contents in BALF to restore Th1/Th2 immune imbalance	Cui et al. (2023)
Cardiovascular protection					
ASA oil	Rats myocardial ischemia-reperfusion injury model	*In vivo*	2, 6, and 10 mL/kg	Shrinking myocardial infarction size, lowering serum and myocardial CK and AST activities, enhancing myocardial CAT, SOD, and GSH-Px levels, while diminishing MDA content, inhibiting iNOS but activating cNOS and heightening NO content	Zhang et al. (2011)
Amygdalin	Left anterior descending coronary artery induced mice myocardial infarction model	*In vivo*	3 mg/kg, i.p	Improving cardiac function and shrinking myocardial infarction size, alleviating myocardial injury and fibrosis by hampering IL-1β, IL-6, and TNF-α mRNA levels, CD68 and COX-2 protein expressions, and TGF-β/Smad signaling pathway activation	Guo et al. (2023)
Amygdalin	Angiotensin II-induced H9C2 cell hypertrophy	*In vitro*	80, 160, and 320 μM	Reducing protein expressions of ANP, BNP, β-MHC, calcineurin, and phospho-GATA-4, intensifying phospho-Nrf2, SOD-2 and CAT protein expressions, impeding phospho-NF-κB p65, COX-2, iNOS, and TNF-α protein expressions	Kung et al. (2021)
Amygdalin	High-fat diet-induced mice atherosclerosis model	*In vivo*	1 mg/kg	Lowering blood triglyceride, total cholesterol, and LDL content, enhancing IL-10 and TGF-β level, up-regulating CD4^+^CD25^+^Foxp3^+^ Treg cells level, Foxp3 mRNA level and protein expression, inducing cell apoptosis	Jiagang et al. (2011)
ASA	Healthy Slovak women in their reproductive age (41.60 ± 11.28 years)	*In vivo*	60 mg/kg for 42 days	Lessening total cholesterol and LDL-C content, while slightly elevating HDL-C content, intensifying follicle stimulating hormone, luteinizing hormone, and prolactin content in plasma, reducing progesterone and 17-β-estradiol content, while heightening the content of testosterone and androstenedione	Kopčeková et al. (2021)
ASA	Healthy adults in Slovak (5 females and 7 males)	*In vivo*	60 mg/kg for 84 days	Diminishing total cholesterol and LDL-C contents in plasma, slightly enhancing HDL-C level, reducing hs-CRP and AST levels and increasing CK and GGT levels	Kopčeková et al. (2018)
ASA	Adults with elevated total cholesterol levels	*In vivo*	60 mg/kg for 42 days	Decreasing total cholesterol and LDL-C content, reducing LDL_1_, LDL_2_, and atherogenic LDL_3-7_ subfractions, increasing mean LDL particle size	Kopčeková et al. (2022)
Neuroprotection					
Amygdalin	LPS-induced BV-2 cells	*In vitro*	0.01, 0.1, and 1 mg/mL	Suppressing COX-1, COX-2, iNOS mRNA levels and protein expressions to impede the production of PGE2 and NO	Chang et al. (2005)
Amygdalin	LPS-induced BV-2 cells	*In vitro*	10 and 100 μg/mL	Hampering the accumulation of PGE2 and NO by inhibiting COX-1, COX-2 and iNOS mRNA levels and protein expressions	Yang et al. (2007)
ASA aqueous extract	H_2_O_2_-induced PC12 cells	*In vitro*	1, 10, and 100 μg/mL	Anti-AchE activity with the IC50 value of 134.93 μg/mL	Vahedi-Mazdabadi et al. (2020)
ASA methanol extract	Haloperidol-induced rats Parkinsonism model	*In vivo*	100, 300, and 800 mg/kg	Improving motor function deficits and behavioral disturbances, alleviating brain tissue injury, strengthening dopamine, noradrenaline, and serotonin levels, while depressing AchE activity in brain homogenates, elevating SOD, CAT, and GSH levels, while reducing MDA and nitrite levels	Saleem et al. (2022)
Amygdalin	PC12 cells	*In vitro*	2.5, 5, 10, and 20 μM	Enhancing NGF-induced neurite outgrowth, and protecting PC12 cells from 6-OHDA-induced injury by up-regulating calreticulin protein expressing and intracellular calcium concentration	Cheng et al. (2015)
Respiratory protection					
Amygdalin	LPS-induced mice lung inflammation model	*In vivo*	0.5, 1, and 2 mg/kg, i.p.	Preventing LPS-induced lung inflammation, reducing W/D ratio of lung tissues and ROS content, suppressing EGFR, phospho-AKT, phospho-SRC, VEGFA, MAPK1, IL-6, TNF-α, IL-1β, and TGF-β1 protein expressions	Wang et al. (2021)
ASA aqueous extract	OVA-induced allergic airway inflammation, and peribronchial lymph node cells	*In vivo* and *in vitro*	1 and 10 mg/mL	Reducing airway hyperreactivity, and numbers of eosinophils neutrophils and lymphocytes in BALF, lowering IL-4 level in BALF, OVA-specific IgE level in serum and BALF, and IgG1 level in serum, while increasing IgG2a level in serum, inhibiting Th2 response by diminishing IL-4, IL-5, and IL-13 production in lymph node cells	Do et al. (2006)
ASA carbonisata-derived carbon dots	LPS-induced acute lung injury	*In vivo*	0.94, 1.88, and 3.75 mg/kg, i.p	Ameliorating LPS-induced acute lung injury by reducing IL-6, IL-1β, and TNF-α levels while intensifying IL-10 content, as well as elevating SOD and GSH content and diminishing MPO and MDA accumulation	Zhao et al. (2022c)
Amygdalin	Cigarette smoke combined with LPS-induced mice COPD model and BEAS-2B cells	*In vivo* and *in vitro*	*In vivo*: 5, 10, and 20 mg/kg; *in vitro*: 100, 200, and 400 μg/mL	Inhibiting EMT process by inhibition of TGF-β/Smad pathway, suppressing vimentin, TGF-β1, phospho-Smad3, and phospho-Smad2 mRNA levels and protein expressions while up-regulating E-cadherin mRNA level and protein expression	Wang et al. (2019)
Amygdalin	LPS-treated BEAS-2B cells	*In vitro*	200, 400 μg/mL	Counteracting LPS-induced apoptosis and inflammatory responses by decreasing apoptosis rate and content of TNF-α, IL-6, IL-8, and MUC5AC. Suppressing LPS-induced EMT and activation of TLR4/NF-κB signaling by inhibiting N-Cadherin, α-SMA, vimentin, TLR4, phospho-p65, phospho-IκBα while intensifying E-Cadherin and IκBα protein expressions	Si and Zhang (2021)
Digestive system protection					
ASA	Irradiation-induced rats parotid glands degenerative model	*In vivo*	400 mg/kg	Suppressing EGF and TGF-β2 levels to alleviate rat parotid gland injury	Abdaulmoneam et al. (2023)
ASA oil	Ethanol-induced rat gastric mucosal injury	*In vivo*	1 mL/rat, i.g.	Relieving gastric mucosa injury by hampering iNOS protein expression, IL-6 and MDA levels while heightening IL-10, CAT and SOD levels	Karaboğa et al. (2018)
Amygdalin	Dibutyltin dichloride-induced rats chronic pancreatitis model	*In vivo*	10 mg/kg	Lessening α-SMA, PDGF-BB, TGF-β1, and ET-1 levels while enhancing CGRP level to alleviate microcirculatory disturbance, attenuates PSCs activation and relieves inflammation	Zhang et al. (2018)
ASA ethanolic extract and amygdalin	PANC-1 cells	*In vitro*	704 μg/mL and 35 mg/mL, respectively	Inducing cell apoptosis by regulating Bax, Bcl-2, and caspase-3 mRNA expression	Aamazadeh et al. (2020)
Antidiabetic					
ASA	Alloxan-induced rats diabetes model	*In vivo*	2, 3, and 4 mg/kg, i.p	Lowering blood glucose, HbA1c, LPO, and α-glucosidase levels and increasing serum insulin and CAT levels	Raafat et al. (2018)
Amygdalin	Streptozotocin-induced rats diabetic retinopathy model, and high-glucose-stimulated HREC cells	*In vivo* and *in vitro*	*In vivo*: 10 mg/kg; *In vitro*: 10, 20, 40, and 80 μM	Relieving diabetic retinopathy progression, intensifying NRF2, HO-1, and NQO1 protein expressions, and CAT, SOD levels, while suppressing LDH, MDA, ROS levels and protein expressions of RAS, TFR1, and ACSL4, decreasing HbA1c, blood glucose levels and increasing body weight	Li et al. (2023)
ASA peptides	Spontaneously hypertensive rats	*In vitro*	50, 100, and 150 mg/kg	Reducing systolic blood pressure and diastolic blood pressure	Qin et al. (2023)
A neutral polysaccharide (AP-1)	Not mentioned	*In vitro*	0.5–10 mg/mL	Scavenging DPPH radicals, ABTS radicals, and hydroxyl radicals, and inhibiting α-glucosidase activity	Peng et al. (2023)
Amygdalin	High glucose-induced rats diabetic nephropathy model and HBZY-1 cells	*In vivo* and *in vitro*	1, 3, and 10 mg/kg	Suppressing ROS, fasting blood glucose, IL-12, IFN-γ, MDA, 24 h-urine proteins, Scr and BUN levels by inhibiting Smad/TGF-β pathway and ECM accumulation as well as transformation	Chen et al. (2021a)
Liver protection					
Amygdalin	Ehrlich ascites carcinoma-induced liver damage mice model	*In vivo*	300 mg/kg	Decreasing tumor volume and number of viable tumor cells, reducing hepatic MDA content, MMP9 and VEGF mRNA levels, while elevating GSH, SOD content and Nrf2 mRNA level	Attia et al. (2022)
ASA	Ethanol-induced rat liver injury	*In vivo*	15% or 30% ASA +20% alcohol-water	Reducing LDH content in serum and MDA production in erythrocyte, brain, kidney, and heart, while heightening SOD and GST content	Yurt and Celik (2011)
Amygdalin	D-galactosamine and LPS-induced mice acute liver injury	*In vivo*	4 and 8 mg/kg	Lowering serum ALT and AST, liver MDA, levels of MPO, TNF-α, IL-6, IL-1β, iNOS and COX-2 by inhibition of NLRP3 inflammasome and NF-κB signaling cascade, and activation of Nrf2/NQO1 signaling pathway	Tang et al. (2019)
Amygdalin and prunasin	CCl4-induced rats liver injury and fibrosis, TGF-β1 stimulated JS1 cells, and LPS-stimulated RAW264.7 cells	*In vivo* and *in vitro*	2.5, 5, and 10 μM	Inhibiting α-SMA, Col1A1, NO, serum AST, serum ALT levels to impede macrophage inflammation and hepatic stellate cell activation	Zhang et al. (2022b)
Amygdalin	Acetaminophen-induced mice acute liver failure model	*In vivo*	2.5 and 5 mg/kg	Reducing ALT, AST, necrosis area, TNF-α, IL-6, IL-1β, MDA, phospho-JUK, phospho-MLKL, and phospho-RIP3 levels, while elevating SOD, Nrf2, NQO1, HO1, and phospho-AKT levels by activation of AKT/JNK/Nrf2 signalling pathway	Zhang et al. (2022a)
kidney protection					
Amygdalin	Unilateral ureteral obstruction induced rats renal fibrosis, and primary kidney fibroblast cells	*In vivo* and *in vitro*	3 and 5 mg/kg	Hampering kidney fibroblast proliferation, TGF-β1 secretion, and renal interstitial fibrosis	Guo et al. (2013)
ASA aqueous extract	Not mentioned	*In vivo*	1,000, 1,500, and 2,000 mg/kg	Diminishing ALT, AST, ALP, BIL, and MDA levels, while increasing creatinine, urea, BUN, CAT, SOD, and GSH levels	Zehra and Naz (2021)
Other pharmacological activities					
ASA volatile oil	HaCaT cells	*In vitro*	1, 2.5, and 5 g/mL	Inducing G0/G1 cell cycle arrest, increasing early and late apoptotic cells, decreasing caspase3, caspase8, caspase9, PARP, Bax, TNF-α and NF-κB p65 protein expressions while intensifying Bcl-2 and IκBα protein expressions	Li et al. (2016)
ASA oil	Primiparous women	*In vivo*	15 min massage per day	Reducing the development of striae gravidarum during pregnancy	Timur Taşhan and Kafkasli (2012)
Dry eye syndrome					
ASA aqueous extract and Amygdalin	Urban particulate matter-induced rats keratoconjunctivitis sicca	*In vivo*	1, 10, and 100 μg/kg for ASA aqueous extract, and 0.1, 1, and 10 μg/mL	Inhibiting MMP activity and down-regulating MMP-9 mRNA level, reducing TNF-α and IL-6 content and mRNA level	Hyun et al. (2019)
ASA methanol aqueous extract	Exorbital lacrimal gland excision-induced mice model	*In vivo*	0.5 and 1 mg/mL	Increasing aqueous tear secretion, alleviating corneal epithelial damage and corneal irregularity, inhibiting Muc4 and TNF-α protein expressions	Kim et al. (2016)
Fracture Healing					
Amygdalin	RANKL-induced RAW264.7 cells	*In vitro*	5, 10, 20, and 40 μM	Impeding osteoclast differentiation and formation, endoplasmic reticulum stress and oxidative stress in by suppressing BIP, phospho-eIFα, ROS, NFATc1, c-fos, dcstamp, acp5, ATP6v0d2, ctsk, phospho-ERK, phospho-P38, and phospho-JUK levels while enhancing CAT and SOD levels	Trang et al. (2022)
Amygdalin	Mice tibial fracture model, and TGF-β1 stimulated C3H10 T1/2 cells	*In vivo* and *in vitro*	10 μM	Promoting the migration and differentiation of MSCs to accelerate the fracture healing process by regulating TGF-β/Smad signaling	Ying et al. (2020)
Immunoregulation					
ASA oil	Cyclophosphamide-induced rats immunosuppression model	*In vivo*	0.5 mL/100 g	Increasing organ indexes of spleen and thymus, white blood cell counts, platelet counts, bone marrow karyocyte counts, IgA, IgM, IgG, IL-2, IL-12, TNF-α. SOD, and GSH-Px levels while decreasing MDA production	Tian et al. (2016)

### 4.5 Cardiovascular protection

The latest evidence indicates that cardiovascular disease is responsible for 31% of global deaths. It has been established that adopting a healthy diet is crucial in reducing the risk of cardiovascular diseases (Dikariyanto et al., 2021). Cardiovascular diseases encompass various heart and vascular conditions such as coronary heart disease, hypertension, heart failure, peripheral vascular disease, cerebrovascular disease, vascular disease, and rheumatic heart disease. ASA, which is rich in unsaturated fatty acids, has been proven to effectively lower biochemical and arterial markers associated with cardiovascular risk (de Oliveira et al., 2017). Moreover, ASA is abundant in anthocyanins, flavonoids, and phenolic acids, with concentrations of up to 118.17 mg/100 g, 113.66 mg/L, and 91.42 mg/100 mL, respectively (Qin et al., 2019). These substances have also demonstrated positive effects on cardiovascular diseases (Perez-Vizcaino and Duarte, 2010; Blesso, 2019; Potì et al., 2019; Mattioli et al., 2020). Therefore, ASA exhibits significant potential and advantages in the treatment of cardiovascular system diseases, mainly due to the functions of unsaturated fatty acids, polyphenols, flavonoids, and amygdalin.

Currently, ASA and its active ingredients have been shown to contribute to cardiovascular health in both *in vivo* experiments and clinical studies (Table 5). In a rat myocardial ischemia-reperfusion injury model, it was observed that continuous treatment with 2, 6, and 10 mL/kg of ASA oil for 2 weeks resulted in a significant reduction in the myocardial infarction area of rats. Additionally, the activities of serum creatine kinase and aspartate aminotransferase increased, leading to an increased production of ATP. This increase in ATP production provides sufficient energy for the physiological needs of the heart. Moreover, supplementation with ASA oil also demonstrated a significant increase in the activity of antioxidant enzymes such as myocardial CAT, SOD, and glutathione peroxidase. This increase in anti-oxidant enzyme activity enhances the anti-oxidant defense system while reducing the content of MDA and inhibiting lipid peroxidation. Ultimately, these effects provide a protective effect against myocardial ischemia-reperfusion injury in cardiomyocytes (Zhang et al., 2011). In recent years, there has been increasing attention on amygdalin, the main component of ASA. It has been demonstrated *in vitro* that amygdalin can effectively inhibit Ang II-induced cardiomyocyte hypertrophy, reduce inflammatory response, and exhibit anti-oxidant activity when treating H9C2 cells induced by Ang II at concentrations of 80, 160, and 320 μM. These effects of amygdalin are primarily achieved through the reduction of atrial natriuretic peptide, B-type natriuretic peptide, and β-MHC, which are related to cardiac hypertrophy. Additionally, amygdalin inhibits the expression of inflammatory markers such as TNF-α, iNOS, COX-2, and phospho-NF-κB protein. Furthermore, amygdalin increases the expression of Nrf2, CAT, SOD-2, and GPX-4, which are proteins related to oxidative stress (Kung et al., 2021). Both *in vitro* and *in vivo* studies have also indicated that amygdalin can alleviate atherosclerosis. This effect may be attributed to its inhibition of the inflammatory response, enhancement of immune regulatory function in regulatory T cells, or inhibition of the TLR4/NF-κB and Bcl-2/Bax signaling pathways (Jiagang et al., 2011; Wang et al., 2022).

The benefits of ASA for cardiovascular disease have been extensively studied due to its various components and proven efficacy. ASA has been shown to exert cardiovascular protective effects by reducing cholesterol levels, particularly low-density lipoprotein cholesterol (LDL-C) (Kopčeková et al., 2021). Clinical research reports have demonstrated that after 6 consecutive weeks of taking 60 mg/kg ASA, volunteers experienced a significant decrease in serum LDL-C levels. It is important to note that elevated levels of LDL-C can contribute to the development of cardiovascular atherosclerosis and the blockage of blood vessels by causing excessive fat absorption in extrahepatic cell tissues (Siri-Tarino et al., 2010). In another clinical study, it was observed that after 12 weeks of taking 60 mg/kg ASA, total cholesterol levels decreased by 8.64% and LDL-C levels decreased by 21.2%. Additionally, there was a slight increase in high-density lipoprotein cholesterol (HDL-C) levels, along with an increase in C-reactive protein and serum creatine kinase levels (Kopčeková et al., 2018). Importantly, studies have shown that for every 1% reduction in LDL-C, the risk of coronary heart disease is reduced by up to 3% (Brown and Goldstein, 2006). This indicates that consuming ASA can significantly reduce the risk of cardiovascular disease. Further investigation revealed that after a 6-week administration of ASA to 21 individuals with normal cholesterol levels and 13 patients with high cholesterol levels, there was no significant change observed in the total cholesterol content and average LDL-C levels of the normal individuals. Similarly, the average total cholesterol content and average LDL-C levels of the patients with HDL-C levels also did not exhibit a significant change. However, a reduction in density cholesterol levels was observed, and the LDL_3–7_ subfractions were only detected in one individual (Kopčeková et al., 2022). It is important to note that the LDL_3–7_ subfractions, which are part of very low-density lipoproteins, have smaller particle sizes compared to LDL_1_ and LDL_2,_ and are associated with a higher risk of atherosclerosis (Qiao et al., 2022). In simpler terms, the intake of ASA can modify the lipoprotein profile of individuals with hypercholesterolemia by primarily reducing low-density lipoprotein levels, without negatively affecting lipid metabolism in healthy individuals.

In summary, ASA exerts cardiovascular protection mainly by reducing LDL levels, inhibiting oxidative stress and regulating immunity, which strongly supports the use of ASA in the management of cardiovascular diseases.

### 4.6 Neuroprotection

Alzheimer’s disease and Parkinson’s disease are two common neurodegenerative diseases characterized by neuronal damage and behavioral dysfunction. The pathological processes involved in these diseases include immune inflammation, oxidative stress, and mitochondrial dysfunction (Chen W. et al., 2022). Phytochemicals with anti-oxidant properties are known to have the potential to provide neuroprotection (Chakraborty et al., 2022). ASA, abundant in flavonoids, polyphenols, and other anti-oxidative compounds, shows promising potential for treating neurodegenerative diseases by suppressing inflammation, oxidative stress and acetylcholinesterase (AchE) activity (Table 5).

Microglia, immune effector cells in the central nervous system, play a role in releasing inflammatory mediators that contribute to neurotoxicity and the development of neurodegenerative diseases (Simpson and Oliver, 2020). Studies have demonstrated that ASA extract can inhibit COX-2 and iNOS mRNA levels in BV2 cells stimulated by LPS. This inhibition leads to a reduction in the synthesis of PGE2 and the production of NO, thereby suppressing immune and inflammatory responses and exerting a neuroprotective effect (Chang et al., 2005; Yang et al., 2007). AchE, present in neurons, serves as an indicator of neuronal damage (Olasehinde and Olaniran, 2022). *In vitro* studies, ASA water extract exhibits significant anticholinesterase activity with an IC50 of 134.93 μg/mL. Additionally, treatment with 100 μg/mL ASA water extract demonstrates a favorable neuroprotective effect against H_2_O_2_-induced damage to PC12 neuron cells, resulting in a cell survival rate of 70.71%. In comparison, PC12 cells treated with 400 μM hydrogen peroxide exhibit a survival rate of less than 40% (Vahedi-Mazdabadi et al., 2020).

It has been demonstrated *in vivo* studies that the methanol extract of ASA at concentrations of 100, 300, and 800 mg/kg has a protective effect on haloperidol-induced Parkinson’s disease model. Behavioral analysis has shown that ASA treatment improves motor activity, motor coordination, and exploratory activities in rats. It also reduces depression, anxiety, and convulsive seizures, accompanied by a decrease in dopamine, 5-hydroxytryptamine, and norepinephrine neurotransmitter levels. Additionally, there is a significant increase and decrease in AchE levels. Furthermore, behavioral improvement and brain function recovery are positively correlated with increased anti-oxidant enzyme activity in the body (Saleem et al., 2022). Moreover, amygdalin also shows potential neuroprotective effects, possibly due to its induction of calreticulin protein expression, which plays a vital role in the survival, differentiation, and regulation of neurons (Cheng et al., 2015).

### 4.7 Respiratory protection

**FIGURE 6:**
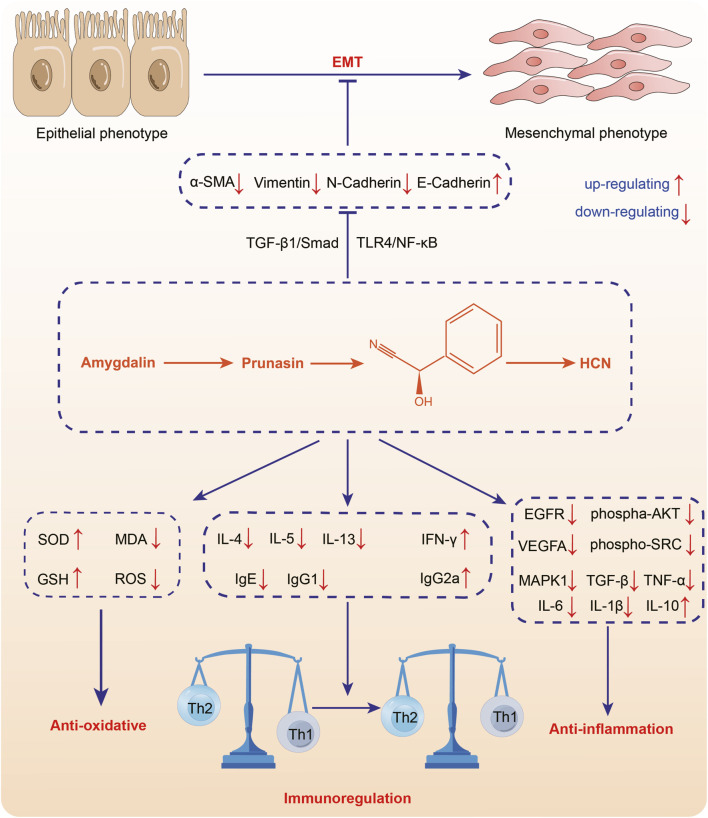
Respiratory protection of *Armeniacae semen amarum*.

Respiratory system diseases are diverse and common, affecting the trachea, bronchi, and lungs. Some prevalent conditions in this category include asthma, COVID-19, acute lung injury, and chronic obstructive pneumonia (Tavares et al., 2020). ASA, an important Chinese herbal medicine, is used to treat cough and has various functions such as enhancing lung function, relieving constipation, and promoting intestinal peristalsis. According to traditional Chinese medicine, bitter purgation helps disperse and move lung Qi, thereby eliminating phlegm (Gao et al., 2011). Pharmacological studies have shown that amygdalin, an effective component of ASA, is hydrolyzed to hydrocyanic acid and benzaldehyde in the body after oral administration, thereby relieving cough, asthma and other respiratory system diseases (Figure 6).

The COVID-19 pandemic, caused by the 2019 novel coronavirus, is spreading globally. It is characterized by symptoms such as fever, dry cough, and fatigue, which can lead to severe respiratory failure and even death. Additionally, patients may experience muscle aches and diarrhea, and in severe cases, they may develop acute respiratory distress syndrome, septic shock, or succumb to the disease (Du et al., 2021). Through network pharmacology and molecular docking, it was found that stigmasterol, sitosterol, sholesterol, (6Z,10E,14E,18E)-2,6,10,15,19,23-hexamethyltetracosa-2,6,10,14,18,22-hexaene, oestrone, diisooctyl succinate, 11,14-eicosadienoic acid, and amygdalin are suggested to be the nine key active ingredients for the treatment of COVID-19. Moreover, IL6, SRC, MAPK1, MAPK3, VEGFA, EGFR, HRAS, and CASP3 are identified as potential core targets for ASA treatment. It has been demonstrated that a therapeutic potential of amygdalin *in vivo* experiments. Moreover, The administration of 0.5–2 mg/kg of amygdalin has been shown to regulate the PI3K-AKT signaling pathway, VEGF signaling pathway, and MAPK signaling pathway, resulting in significant inhibition of EGFR, phospho-AKT, phospho-SRC, VEGFA, MAPK1, IL-6, IL-1β, and TNF-α protein expressions (Wang et al., 2021). However, more research is required to support the use of ASA in the treatment of COVID-19.

Allergic asthma, which is the most common type of asthma, is characterized by chronic airway inflammation involving T lymphocytes, mast cells, eosinophils, and other cells (Possa et al., 2013). Several studies have demonstrated that ASA aqueous extract shows promising therapeutic effects in both an ovalbumin-induced allergic airway inflammation model *in vivo* and lymph node primary cells *in vitro*. This therapeutic effect of ASA is attributed to a reduction in IL-4 and IL-5 levels (Do et al., 2006). IL-4 is responsible for the transformation of regulatory T cells into helper T cells, while IL-5 regulates the growth, differentiation, and activation of eosinophils (Jin et al., 2019). However, further research is necessary to determine whether ASA exhibits similar therapeutic effects on other types of asthma and to investigate the underlying molecular mechanisms involved.

Acute lung injury (ALI) is a severe medical condition associated with significant morbidity and mortality. It is characterized by damage to the alveolar epithelial cells and pulmonary capillary endothelial cells, resulting from non-cardiogenic factors (Tang et al., 2023). The clinical manifestations of ALI include dyspnea and intractable hypoxemia, which can progress to severe respiratory disorders. ALI is characterized by the infiltration of a large number of neutrophils into lung tissue, leading to the release of inflammatory cytokines and damage to pulmonary endothelial and epithelial cells. LPS, also known as endotoxin, is a major component of the outer membrane of Gram-negative microorganisms and is highly pathogenic (Liu et al., 2020). The ASA carbon nano-material has demonstrated its ability to inhibit the release of IL-6, IL-1β, and TNF-α inflammatory mediators in rat serum. Moreover, it has been shown to reduce the increase of neutrophils in the blood. Additionally, it exhibits a decrease in the chemotaxis of neutrophils to inflammatory sites and inhibits the injury and aggravation of LPS to lung tissue. These findings suggest that ASA carbon nano-material shows promising potential as a candidate treatment for ALI (Zhao Y. et al., 2022).

In addition, amygdalin may also have therapeutic effects on chronic obstructive pulmonary disease (COPD) (Sun et al., 2020). COPD is characterized by airway remodeling, which involves epithelial-mesenchymal transition (EMT). Recent studies have shown that amygdalin, administered at doses of 5, 10, and 20 mg/kg, has a protective effect on the EMT process in COPD mice induced by cigarette smoke. These findings are consistent with the observed inhibition of TGF-β1 protein expression and Smad2/3 phosphorylation by amygdalin, indicating its potential role in suppressing the TGF-β/smad pathway. Moreover, amygdalin also demonstrates inhibitory effects on the EMT process in BEAS-2B cells stimulated by cigarette smoke *in vitro*, suggesting its potential use in COPD treatment (Wang et al., 2019). Furthermore, the mechanism by which amygdalin exerts its therapeutic effect may also be related to the inhibition of LPS-induced EMT and TLR4/NF-κB signaling cascade (Si and Zhang, 2021).

Numerous formulas containing ASA have been extensively studied and utilized in the research and treatment of various respiratory diseases such as colds, asthma, COVID-19, and pulmonary fibrosis (Li et al., 2010; Lin et al., 2016; Sun et al., 2018; Bai et al., 2022; Li et al., 2022). This further demonstrates the potential respiratory protection activity of ASA (Table 5).

### 4.8 Digestive system protection

Limited reports exist on the protective effects of ASA on the digestive system. This section provides a summary of the protective effects of ASA on the digestive tract and digestive glands (Table 5). Studies have shown that 400 mg/kg ASA can enhance the damage caused by gamma-radiation of 5 Gy to the salivary glands of *Rattus Norvegicus*, specifically affecting the acinar cells. This effect is primarily attributed to the downregulation of EGF protein expression and the upregulation of TGF-β protein expression, indicating that ASA mitigates oxidative damage and inflammatory responses, thereby protecting against salivary gland damage (Abdaulmoneam et al., 2023). In addition, ASA oil has been found to possess gastroprotective effects. In an ethanol-induced rat gastric ulcer model, ASA oil reduces the release of cytokines such as IL-6, increases levels of oxidative stress markers like SOD and CAT, decreases lipid oxidation, and inhibits mucosal cell apoptosis, demonstrating its gastroprotective properties. Recent research also suggests that amygdalin may have potential pancreatic protective effects (Karaboğa et al., 2018). Intravenous injection of 10 mg/kg amygdalin improves pancreatic fibrosis in rats with chronic pancreatitis induced by dibutyldichlorotin, as evidenced by reduced production of profibrotic growth factors and inhibition of pancreatic stellate cell activation. The mechanism may involve improved microcirculation through reduced endothelin-1 expression and upregulated expression of calcitonin gene-related peptide (Zhang et al., 2018). Similarly, ASA ethanol extract can induce apoptosis of pancreatic cancer cells *in vitro* (Aamazadeh et al., 2020).

In summary, ASA has been found to have a protective effect on parotid glands, pancreas and stomach. Its mechanism of action is believed to involve the inhibition of inflammatory response and oxidative stress, along with the induction of cell apoptosis. However, the specific substances responsible for the therapeutic effects of ASA are still unidentified and the protective effects on other digestive organs and digestive glands have not been defined, thus the protective effects of ASA on the digestive system need to be further investigated.

### 4.9 Antidiabetic effect

Diabetes mellitus (DM) is a group of metabolic disorders that poses a significant global health burden, affecting approximately 6% of the population. The majority of diabetic patients (90%–95%) have type II diabetes, while the remaining have type I diabetes. Currently, the options for DM treatment are limited, and long-term use of available drugs may result in severe side effects (Das and Chakrabarti, 2005). ASA has shown specific effects on DM and offers a promising alternative treatment option due to its cost-effectiveness and easy accessibility. Both *in vivo* and *in vitro* studies have demonstrated that the antidiabetic activity of ASA is primarily associated with its ability to enhance insulin secretion, leading to reduced blood pressure and mitigation of oxidative stress (Table 5).

In an alloxan-induced rat DM, ASA demonstrated a dose-dependent reduction in blood glucose levels, an increase in body weight, a decrease in lipid peroxidation levels, and an increase in serum CAT levels. ASA significantly increased insulin levels after 8 weeks, and exhibited an inhibitory effect on α-glucosidase, suggesting that its anti-diabetic properties may be attributed to the reduction of oxidative stress caused by glucose, inhibition of α-glucosidase, and significant mediation by elevated insulin (Raafat et al., 2018). Interestingly, ASA also showed a significant reduction in glycosylated hemoglobin levels, indicating its potential to prevent complications associated with DM. Higher levels of Hemoglobin A1C (HbA1c) in diabetic patients are indicative of poorer regulation of blood glucose and an increased risk of diabetes-related complications (Klonoff, 2020). Furthermore, amygdalin was found to alleviate diabetic retinopathy, a complication of DM. In high glucose-stimulated HRECs cells, 40 μM amygdalin demonstrated a significant inhibition on oxidative stress and ferroptosis, evidenced by increased GSH/GSSG ratio, SOD, CAT, GPX4 activity and reduced MDA and ROS levels, as well as significant downregulation of ferroptosis marker proteins including RAS, TFR1, and ACSL4. Notably, the antidiabetic retinopathy effects of amygdalin were found to be associated with the activation of the NRF2/ARE pathway, leading to the activation of NRF2 and HO-1 and an increase in NQO1 protein expression (Li et al., 2023).

Recently, the antihypertensive effects of natural chemical constituents of ASA have attracted great attention from researchers. A polypeptide, Arg-Pro-Pro-Ser-Glu-Asp-Glu-Asp-Gln-Glu, has been identified in ASA albumin lately. This polypeptide acts as a non-competitive inhibitor of angiotensin-converting enzyme (ACE) with an IC50 value of 205.50 μM. Additionally, it has exhibited positive antihypertensive effects on spontaneously hypertensive rats at concentrations of 100 and 150 mg/mL. Although not as effective as 10 mg/kg captopril, this polypeptide has led to a significant decrease in systolic and diastolic blood pressure (Qin et al., 2023). These findings suggest that the polypeptide holds the potential for anti-DM effects and could be utilized in the development of anti-DM drugs. Furthermore, a neutral polysaccharide (AP-1), which has a triple helix structure, has recently been extracted from ASA. AP-1 primarily consists of glucose, arabinose, galactose, and mannose. It has strong inhibition of α-glucosidase enzyme and the ability to scavenge DPPH, ABTS, and Hydroxyl free radicals *in vitro* (Peng et al., 2023). These findings indicate that AP-1 may serve as a natural anti-oxidant and hypoglycemic agent in the treatment of DM.

### 4.10 Liver protection

Oxidative stress is widely recognized as the underlying cause of both acute and chronic liver diseases (Cui et al., 2021). ASA, a natural source of plant antioxidants, shows promising potential for the treatment of liver diseases. Recent studies have revealed that amygdalin not only alleviates symptoms of Ehrlich ascites cancer but also, helps prevent liver cancer and mitigate associated liver damage when combined with sorafenib. These hepatoprotective effects are attributed to the direct reduction of liver function indicators such as alanine aminotransferase (ALT), aspartate aminotransferase (AST), and gamma-glutamyl transferase (GGT), as well as the significant antioxidant activity of amygdalin (Attia et al., 2022). In addition, another study also suggests that the key role of ASA in liver protection may be related to oxidative stress (Yurt and Celik, 2011).

ASA has demonstrated hepatoprotective effects at various stages of liver disease development. In the early stages, ASA exhibits anti-inflammatory properties, effectively inhibiting disease progression. The main component of ASA, amygdalin, not only inhibits excessive oxidative stress and reduces the levels of liver injury-related enzymes, but also suppresses the production of TNF-α, IL-6 and IL-1β as well as the expressions of inflammation-related proteins such as iNOS and COX-2, thereby mitigating inflammatory response and providing resistance against acute liver injury (Tang et al., 2019). Hepatic fibrosis, a compensatory pathophysiological process, occurs when the liver is damaged by chronic inflammation, leading to tissue degeneration, inflammatory infiltration, necrosis, and constant repair of liver collagen and extracellular matrix (Tsuchida et al., 2018). Amygdalin, the active ingredient of ASA, has been found to inhibit the activation of hepatic stellate cells induced by transforming growth factors. It also reduces the secretion of cytokines and the levels of ALT and AST, exerting anti-inflammatory effects and protecting the liver from fibrosis (Zhang et al., 2022b). Moreover, amygdalin has a protective effect on advanced liver failure. In the case of acetaminophen-induced acute liver failure, intraperitoneal injection of 2.5 or 5 mg/kg amygdalin has been found to reduce the area of necrosis in liver tissue, lower the levels of liver function-related indicators ALT and AST, and decrease neutrophil and macrophage counts. These effects are associated with the inhibition of oxidative damage, increased protein expression of Nrf2/NQO1/HO1, phospho-AKT, and inhibition of the JNK/RIP3/MLKL signaling pathway (Zhang et al., 2022a).

**FIGURE 7:**
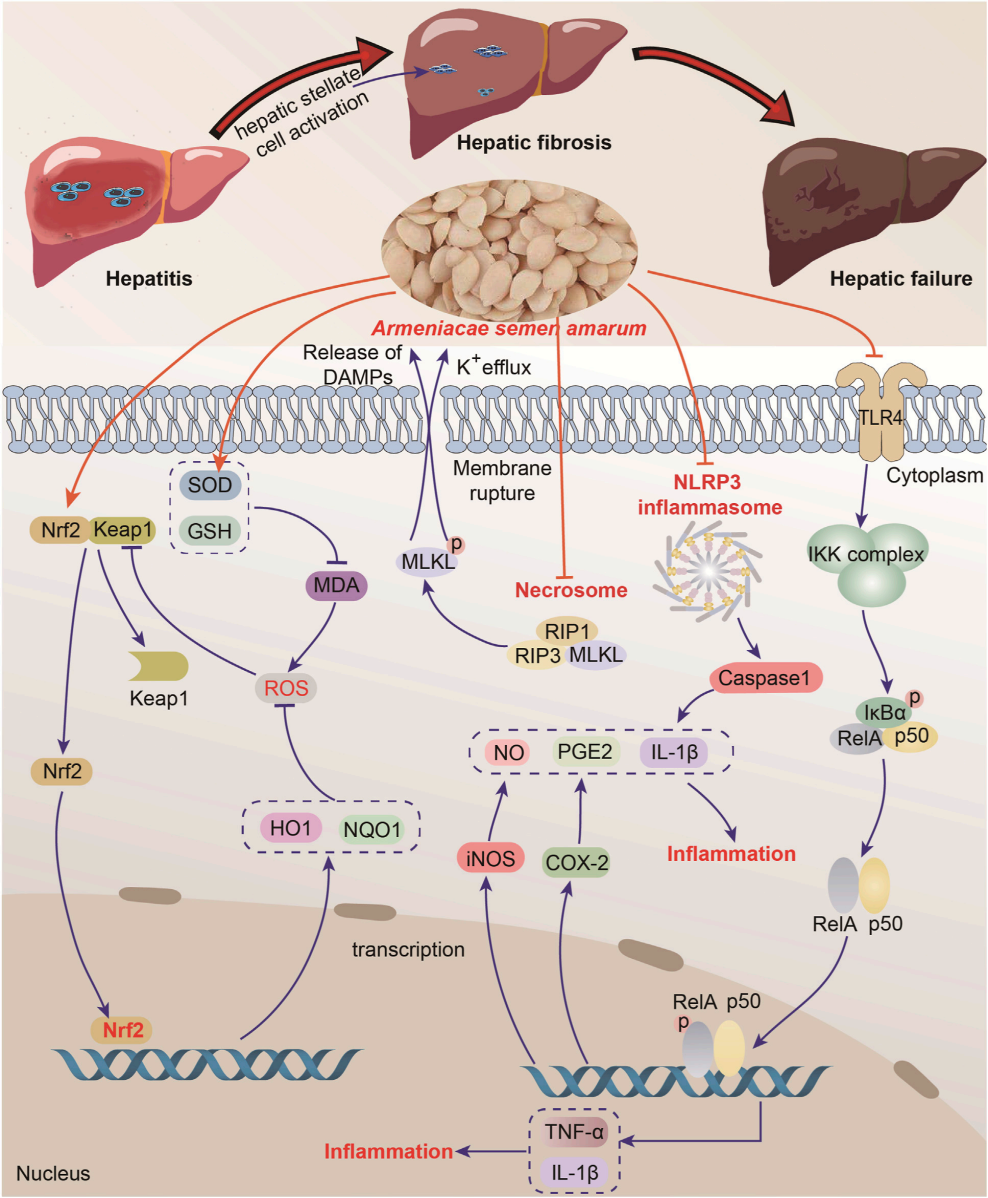
Liver protection of *Armeniacae semen amarum*.

Overall, ASA and amygdalin have promising liver protection effects both *in vivo* and *in vitro* experiments due to their potent anti-oxidant activities (Table 5). However, further research is needed to explore the potential of ASA as a therapeutic drug for different stages of liver disease development (Figure 7).

### 4.11 Kidney protection

ASA has therapeutic effects on both renal cell carcinoma and chronic kidney disease, such as renal fibrosis (Table 5). The main component of ASA, amygdalin, inhibits the proliferation and production of transforming growth factors in renal interstitial fibroblasts, which plays a crucial role in the development of renal interstitial fibrosis (Bai et al., 2020). In a rat model of unilateral ureteral obstruction, treatment with amygdalin at concentrations of 3 and 5 mg/kg resulted in reduced renal damage and delayed progression of renal interstitial fibrosis (Guo et al., 2013). However, the accumulation of hydrocyanic acid, a metabolite of amygdalin in ASA, can lead to nervous system depression, limiting its application. Nevertheless, a study found that oral administration of 2 g/kg ASA water extract to rats did not exhibit nephrotoxicity but increased antioxidant activity, manifesting as increased levels of renal function indicators such as urea, creatinine and urea nitrogen as well as increased activities of anti-oxidant enzymes such as SOD and GSH (Zehra and Naz, 2021). In short, although the metabolism of ASA can lead to the accumulation of toxic substances, its rich natural chemical components have shown promising effects in the research of various diseases. Further research is needed to fully understand the impact of ASA on kidney diseases.

### 4.12 Other pharmacological activities

In addition to its pharmacological effects described above, ASA oil also exhibits skin protective effects. It can inhibit the growth of human keratinocytes and enhance their programmed cell death, making it a potential treatment option for psoriasis (Li et al., 2016). Furthermore, preliminary clinical studies have shown that massage with ASA oil during early pregnancy can effectively reduce the formation of stretch marks (Timur Taşhan and Kafkasli, 2012). Additionally, ASA extract has demonstrated positive effects in relieving symptoms of dry eye syndrome and dry keratitis (Kim et al., 2016; Hyun et al., 2019). Moreover, ASA has also been found to promote fracture healing (Ying et al., 2020; Trang et al., 2022) and regulate the immune system (Tian et al., 2016) (Table 5).

## 5 Clinical applications

There is mounting evidence supporting the use of ASA in the treatment of cough, lung, and other respiratory-related diseases. Studies have shown that ASA liquids can reduce the sensitivity of the trachea to ammonia stimulation, thereby relieving cough and promoting intestinal peristalsis (Gao et al., 2012). In a research study investigating the effectiveness of traditional Chinese medicine compounds for treating COVID-19, a total of 166 compounds containing 179 traditional Chinese medicines were collected. Among the candidate prescriptions for COVID-19 treatment selected through complex system entropy and unsupervised hierarchical clustering, ASA ranked third in terms of frequency of use and was included in the first formula (Luo et al., 2020). Furthermore, a data mining analysis examining traditional Chinese medicine prescriptions for respiratory diseases analyzed 562 prescriptions specifically targeting the respiratory system. The results revealed that ASA was utilized in 36.7% of the prescriptions, ranking second after *Glycyrrgizae radix et rhizoma*—roots and rhizomes of *Glycyrrhiza glabra* L. (Fabaceae), which was used in 47.2% of the prescriptions (Fu et al., 2013). These findings suggest that ASA holds promise as an effective treatment for respiratory diseases.

The plant kingdom contains many substances that may have the potential to prevent or treat human disease (Zhao et al., 2022b; Sun et al., 2023; Zhao et al., 2023), but these bioactive components (such as vitamins and alkaloids) usually show low bioavailability or biological instability. Recently, various techniques for improving drug delivery have been developed to solve the problems of bioavailability and stability. Nanoparticle is the most promising drug carrier, which can effectively deliver bioactive compounds and improve bioavailability. Currently, a protein belonging to the 11S globulin family was isolated from ASA water extract. This protein is composed of three polypeptides connected by disulfide bonds. Upon heat treatment, these bonds rearrange, resulting in the formation of a spherical-shaped dimer. The unique structure of this protein makes it a potential candidate for use as a nanocarrier. It efficiently encapsulates paclitaxel with a maximum encapsulation efficiency of 92.6% and a maximum release of paclitaxel of 57.4% (Lin et al., 2020). Additionally, a recent study developed liposomes loaded with amygdalin using a molar ratio of Tween 60: cholesterol: dihexadecyl phosphate as 1: 2: 0.1. These liposome-loaded amygdalin formulations demonstrated significant effects in reducing tumor volume, decreasing epidermal hyperplasia, and eliminating edema in a rat tumor model induced by 7,12-dimethylphenanthrene. Surprisingly, the anti-tumor activity of these liposomes surpassed that of tamoxifen, a well-known anti-tumor drug (El-Ela et al., 2022). Moreover, a polypeptide extracted from ASA water extract has displayed the ability to form a complex with zinc ions, exhibiting remarkablely lowering blood pressure effect. This polypeptide shows promise for further development as an antihypertensive drug (Qin et al., 2023).

Numerous studies have demonstrated that formula preparations containing ASA exhibit powerful therapeutic effects in the treatment of lung disease, liver disease, eye disease, and other diseases, especially respiratory diseases. The ASA-containing formulas may significantly relieve symptoms such as fever, cough and runny nose. Table 6 provides a summary of ASA-containing formulations and their clinical applications as outlined in the Chinese Pharmacopoeia 2020 edition.

**TABLE 6:** The clinical uses of ASA.

No.	Formula name	Main compositions	Traditional and clinical uses	Reference
1	Juhong Capsules	**ASA**; Citri Grandis Exocarpium; Citri Reticulatae Pericarpium; Pinelliae Rhizoma Praeparatum; Poria; Glycyrrhizae Radix et Rhizoma; Platycodonis Radix; Perillae Fructus (stir-fried); Asteris Radix et Rhizoma; Farfarae Flos; Trlchosanthis Pericarpium; Fritillariae Thunbergii Bulbus; Rehmanniae Radix; Ophiopogonis Radix; Gypsum Fibrosum	Phlegm-heat cough with profuse yellow thick greasy sputum, oppression in the chest, and dry mouth	Chinese Pharmacopoeia Commission (2020)
2	Juhong Huatan Pills	ASA; Citri Grandis Exocarpium; Physalis Calyx seu Fructus; Fritillariae Cirrhosae Bulbus; Papaveris Pericarpium; Schisandrae Chinensis Fructus; Alumen; Glycyrrhizae Radix et Rhizoma	Pattern of internal phlegm-turbidity obstruction due to lung Qi deficiency, manifested by cough with sputum, wheezing, panting, fullness and oppression in the chest and the diaphragm	
3	Juhong Tanke Mixture	**ASA**; Citri Grandis Exocarpium; Stemonae Radix (stir-baked with honey); Poria; Pinelliae Rhizoma (processed); Cynanchi Stauntonii Rhizoma et Radix; Glycyrrhizae Rhizoma et Radix; Schisandrae Chinensis Fructus	Pattern of phlegm turbidity obstructing the lung, manifested as cough, wheezing, and profuse sputum. Common cold, bronchitis, and laryngopharyngitis with the symptoms described above	
4	Zhike Juhong Mixture	ASA (peeled and stir-baked); Citri Grandis Exocarpium; Citri Reticulatae Pericarpium; Pinelliae Rhizoma Praeparatum; Peria; Farfarae Flos; Glycirrhizae Radix et Rhizoma; Trichosanthis Pericarpium; Asteris Radix et Rhizoma; Ophiopogonis Radix; Anemarrhenae Rhizoma; Platycodonis Radix; Rehmanniae Radix; Gypsum Fibrosum; Perillae Fructus (stir-baked)	Cough with profuse sputum, oppression in the chest, shortness of breath, dry and itching throat due to phlegm-heat obstructing the lung	
5	Shema Mixture	**ASA**; Ephedrae Herba; Arisaema Cum Bile; Gypsum Fibrosum; Mori Cortex (processed with honey); Belamcandae Rhizoma; Raphani Semen (stir-baked); Cynanchi Stauntonii Rhizoma et Radix; Scutellariae Radix; Schisandrae Chinensis Fructus (processed with vinegar)	Cough with profuse and sticky sputum, oppression in the chest, panting, rattling sound in the throat, fever in some patients, yellow or yellow and white tongue coating, or red tongue, and wiry and slippery or slippery and rapid pulse	
6	Kechuanning Mixture	**ASA**; Ephedrae Herba; Gypsum Fibrosum; Platycodonis Radix; Stemonae Radix; Papaveris Pericarpium; Glycyrrhizae Radix et Rhizoma	Frequent cough, expectoration of yellow sputum, wheezing and panting, and oppression in the chest due to phlegm-heat obstructing the lung	
7	Yifei Qinghua Concentrated Decoction	**ASA**; Astragali Radix; Codonpsis Radix; Glehniae Radix; Ophiopogonis Radix; Agrimoniae Herba; Bistortae Rhizoma; Patriniae Herba; Oldenlandiae Diffusae Herba; Paeoniae Radix Alba; Asteris Radix et Rhizoma; Platycodonis Radix; Glycyrrhizae Radix er Rhizoma	Shortness of breath, lack of strength, cough, hemoptysis chest pain due to dual deficiency of Qi and Yin; Adjuvant therapy against advanced lung cancer with the symptoms described above	
8	Kugan Granules	**ASA**; Ephedrae Herba; Menthae Haplocalycis Herba; Cicadae Periostracum; Lonicerae Japonicae Flos; Scutellariae Radix; Platycodonis Radix; Fritillariae Thunbergii Bulbus; Glycyrrhizae Radix et Rhiwma	Aversion to cold, fever, headache, sore throat, cough, expectoration and panting due to wind-heat cold and lung heat caused by wind-heat. Upper respiratory tract infection, influenza and acute tracheitis and bronchitis with the symptoms described above	
9	Ermu Ansou Pills	**ASA**; Anemarrhenae Rhizoma; Scrophulariae Radix; Papaveris Pericarpium; Ophiopogonis Radix; Farfarae Flos; Asteris Radix et Rhizoma; Lilii Bulbus; Fritillariae Thunbergii Bulbus	Persistent cough in consumptive diseases, manifested as cough with phlegm or wheezing, bone-steaming tidal fever, hoarse voice, dry mouth and tongue, profuse sputum and drooling	
10	Jiusheng Powder	**ASA**; Atractylodis Rhizoma; Phellodendri Chinensis Cortex; Perillae Folium; Menthae Haplocalycis Herba; Olibanum; Myrrha; Calomelas; Hydrargyri Oxydum Rubrum	Damp toxin obstructing the skin, leading to eczema, chronic ulcer in the leg, and impetiginous sores, manifested by wet, oozing and ulcerative skin infections with pus discharge	
11	Ertong Qingfei Pills	**ASA (stir-baked)**; Ephedrae Herba; Gypsum Fibrosum; Glycyrrh_izae Radix et Rhizoma; Mori Cortex (stir-baked with honey); Trichosanthis Pericarpium; Scutellariae Radix; Isatidis Radix; Citri Exocarpium Rubrum; Pinelliae Rhizoma Praeparatum; Perillae Fructus (stir-baked); Descurainiae Semen Lepidii Semen; Fritillariae Thunbergii Bulbus; Perillae Folium; Asari Radix et Rhizoma; Menthae Haplocalycis Herba; Eriobotryae Folium (stir-baked with honey); Cynanchi Stauntonii Rhizoma et Radix; Peucedani Radix; Acori Tatarinowii Rhizoma; Trichosanthis Radix; Chloriti Lapis (calcined)	Wind cold fettering the exterior with phlegm-heat in the lung meridian in pediatric patients, manifested as fever with reddened complexion, cough and wheezing, profuse, thick arid greasy sputum, sore throat arid hoarse voice	
12	Ergan Tuirening Mixture	**ASA**; Artemisiae Annuae Herba; Isatidis Radix; Chrysanthemi Flos; Platycodonis Radix; Forsythiae Fructus; Menthae Haplocalycis Herba; Glycyrrhizae Radix et Rhizoma	Externally contracted wind heat and internal constraint heat transforming into fire in pediatric patients, manifested as headache with fever, cough, and swollen sore throat	
13	Zhisou Huatan Pills	**ASA**; Papaveris Pericarpium; Platycodonis Radix; Anemarrhenae Rhizoma; Peucedani Radix; Citri Reticulatae Pericarpium; Rhei Radix et Rhizorna (processed); Glycirrhizae Radix et Rhizoma praeparata cum Melle; Fritillariae Cirrhosae Bulbus; Gypsum Fibrosum; Perillae Folium; Descurainiae Semen Lepidii semen; Farfarae Flos (processed); Stemonae Radix (processed); Scrophulariae Radix; Ophiopogonis Radix; Buddlejae Flos; Asparagi Radix; Schisandrae Chinensis Fructus (processed); Aurantii Fructus (stir-baked); Trichosanthis Semen; Pinelliae Rhizoma (processed with ginger juice); Aucklandiae Radix; Aristolochiae Fructus (processed); Mori Folium	Pattern of phlegm-heat obstructing the lung, manifested as persistent cough, hemoptysis, sputum, wheezing and Qi counterflow, inability to sleep because of coughing and dyspnea	
14	Zhisou Dingchuan Mixture	**ASA**; Ephedrae Herba; Glycirrhizae Radix et Rhizoma; Gypsum Fibrosum	Pattern of exterior cold with internal heat, manifested as body fever with thirst, cough with profuse expectoration, wheezing and panting, fullness and oppression in the chest and the diaphragm; Acute bronchitis with the symptoms described above	
15	Fenghan Kesou Granules	**ASA;** Citri Reticulatae Periearpiilln; Zlrigiberis Rhizoma Rcess; Pillelliae Rhizoma Praeparatun; Citri Reticulatae Pericapium Viride; Ephedrae Herba; Perinae Folium; Schisandrae Chinensis Fructus; Mori Cortex; Glycyrrhizae Radix et Rhizoma Praeparata cum Melle	Cough and panting due to externally contracted wind-cold and lung Qi failing to diffuse, manifested as headache, stuffy nose, profuse sputum, cough, oppression in the chest and wheezing	
16	Ruyi Dingchuan Tablets	**ASA**; Gecko; Bufonis Venenum (processed); Astragali Radix; Pheretima; Ephedrae Herba; Codonopsis Radix; Ginkgo Semen; Aurantii Fructus Immaturus; Asparagui Radix; Schisandrae Sphenantherae Fructus (steamed with wine); Ophiopogonis Radix; Asteris Radix et Rhizoma; Stemonae Radix; Lycii Fructus; Rehmanniae Radix Praeparata; Polygalae Radix; Lepidii Semen; Daturae Flos; Gypsum Fibrosum; Glycyrrhizae Radix et Rhizoma Praeparata cum Melle	Chronic cough and panting, weak constitution and profuse sputum due to dual deficiency of Qi and Yin; bronchial asthma, pulmonary emphysema, and pulmonary heart disease with the symptoms described above	
17	Kechuanshun Pills	**ASA**; Perillae Fructus; Trichosanthis Semen; Poria; Houttuyniae Herba; Pinelliae Rhizoma (processed); Farfarae Fies; Mori Cortex; Peucedani Radix; Asteris Radix et Rhizoma; Citri Reticulatae Pericarpium; Glycyrrhizae Radix et Rhizoma	Pattern of phlegm turbidity obstructing the lung and lung Qi failing to diffuse, manifested as cough, wheezing, profuse sputum, oppression in the chest; chronic bronchitis, bronchial asthma, and pulmonary emphysema with the symptoms described above	
18	Yangshen Baofei Pill	**ASA**; Papaveris Pericarpium; Schisandrae Chinesis Fructus (stir-baked with vinegar); Fritillariae Cirrhosae Bulbus; Citri Reticulatae Pericarpium; Amomi Fructus; Aurantii Immaturus Fructus; Ephedrae Herba; Gypsum Fibrosum; Glycyrrhizae Radix et Rhizoma; Scrophulariae Radix; Panacis Quinquefolii Radix	Pattern of Yin deficiency and lung heat, manifested as cough with phlegm, panting, oppression in the chest, shortness of breath, dry mouth and throat, and restlessness at night	
19	Runfei Zhisou Pills	**ASA (stir-baked)**; Asparagi Radix; Rehmannlae Radix; Trichosanthis Radix; Trichosanthis Semen (stir-baked with honey); Mori Cortex (stir-baked with honey); Perillae Fructus (stir-baked); Asteris Radix et Rhizom; Fritillariae Thunbergii Bulbus; Farfarae Flos; Platycodonis Radix; Schisandrae Chinesis Fnictus (processed with vinegar); Peucedani Radix; Citri Reticulatae Pericarpium Viride (processed with vinegar); Citri Reticulatae Pericaipium; Astragali Radix Praeparata cum Melle; Ziziphi Spinosae Semen (stir-baked); Scutellariae Radix; Anemarrhenae Rhizoma; Loophatheri Herba; Glycyrrhizae Radix et Rhizoma Praeparata cum Melle	Cough, wheezing, panting, excessive sputum and drooling, and hoarseness due to lung Qi deficiency	
20	Sangjiang Ganrnao Tablets	**ASA**; Mori Folium; Chrysanthemi Flos; Perillae Folium; Forsythiae Fructus; Zingiberis Rhizoma	Common cold due to externally contracted wind-heat, and phlegm turbidity obstructing the lung, manifested as fever, headache, swollen sore throat, and cough with white sputum	
21	Sangju Ganrnao Mixture	**ASA**; Mori Folium; Chrysanthemi Flos; Forsythiae Fructus; Menthae Haplocalycis Herba; Platycodonis Radix; Glycyqhizae Radix et Rhizoma; Phragmitis Rhizoma	Early onset of common cold due to wind-heat, manifested as headache, cough, dry mouth, and sore throat	
22	Maren Pills	**ASA**; Cannabis Semen; Rhei Radix et Rhizoma; Aurantii Fructus Immaturus (stir-baked); Magnoliae Officinalis Cortex (processed with ginger); Paeoniae Radix Alba (stir-baked)	Constipation due to intestinal dryness and body fluid deficiency, manifested as dry feces, and abdominal distension and discomfort; habitual constipation with the symptoms described above	
23	Maren Runchang Pills	**ASA (peeled and stir-baked)**; Cannabis Semen; Rhei Radix et Rhizoma; Aucklandiae Radix; Citri Reticulatae Pericarpium; Paeoniae Radix Alba	Heat in the stomach and intestines with chest and abdominal distension, and constipation	
24	Maren Zipi Pills	**ASA (peeled and stir-baked)**; Rhei Radix et Rhizoma (processed); Cannabis Semen; Angenlia Senesis Radix; Magnolia Officinalis Cortex (processed with ginger); Aurantii Fructus Imrnaturus (stir-baked with bran); Pruni Semen; Paeoniae Radix Alba	Constipation, chest and abdominal distension, loss of appetite, irritability, red tongue with fluid deficiency due to enterogastric heat, intestinal dryness and body fluid deficiency	
25	Qingfei Huatan Pills	**ASA**; Scutellariae Radix (processed with wine); Trichosanthis Semen; Fritillariae Cirrhosae Bulbus; Arisaema cum Bile (stir-baked with sand); Pinelliae Rhizoma Praeparatum (stir-baked with sand); Citri Reticulatae Pericarpium; Poria. Aurantii Fructus (stir-baked with bran); Ephedrae Herba (processed with honey); Platycodonis Radix; Perillae Typicae Fructus; Raphani Semen (stir-baked); Farfarae Flos (processed with honey); Glycyrrhizae Radix et Rhizoma	Lung-heat cough and inhibited lung Qi, manifested as profuse sputum and even wheezing, and difficulty in breathing	
26	Qingqi Huatan Pills	**ASA**; Scutellariae Radix (stir-baked with wine); Thchosanthis Semen Pulveratum; Pinelliae Rhizoma (processed); Arisaema Cum Bile; Critri Reticulatae Pericarpium; Aurantii Fructus lrnmaturus; Poria	Profuse sputum, yellow thick greasy sputum, cough, fullness and oppression in the chest and the abdomen due to phlegm-heat obstructing the lung	
27	Qingfei Xiaoyan Pills	**ASA (stir-baked)**; Ephedrae Herba; Gypsum Fibrosum; Pheretima; Arctii Fructus; Lepidii Semen or Descurainiae Semen; Bovis Calculus Artifactus; Saigae Tataricae Comu	Pattern of phlegm-heat obstructing the lung, manifested as coughing and wheezing, distending pain in the hypochondrium, and yellow thick greasy sputum; Upper respiratory tract infection, acute bronchitis, acute episode of chronic bronchitis, lung infections with the symptoms described above	
28	Lusika Pills	**ASA**; Ephedrae Herba; Gypsum Fibrosum; Glycyrrhizae Radix et Rhizoma; Asari Radix et Rhizoma; Perillae Fructus (stir-baked); Sinapis Semen (stir-baked); Arctii Fructus (stir-baked); Trichosanthis Pericarpium; Belamcandae Rhizoma; Indigo Naturalis; Meretricis Concha or Cyclinae Concha; Trichosanthis Radix; Gardeniae Fructus (stir-baked with ginger); Bovis Calculus Artifactus	Whooping cough, cough due to phlegm turbidity obstructing the lung, manifested as paroxysmal cough, rattling sound in the throat, wheezing, dry throat, and hoarse voice; pertussis with the symptoms described above	
29	Lianhuaqingwen Capsules	**ASA (stir-baked)**; Forsythiae Fructus; Lonicerae Japonicae Flos; Ephedrae Herba (processed with honey); Gypsum Fibrosum; Isatidis Radix; Dryopteridis Crassirhizoma Rhizoma; Houttuyniae Herba; Pogostemonis Herba; Rhei Radix et Rhizorna; Rbodiolae Crenulatae Radixet Rhizorna; Menthol; Glycyrrhizae Radix et Rhizorna	Patterns of heat toxin assailing the lung in influenza, manifested as fever, aversion to cold, muscle soreness, stuffy and runny nose, cough, headache, dry and sore throat, reddish tongue, and yellow or yellow and greasy tongue coating	
30	Qingxuan Zhike Granules	**ASA (stir-baked)**; Mori Folium; Menthae Haplocalycis Herba; Platycodonis Radix; Paeoniae Radix Alba; Aurantii Fructus; Citri Reticulatae Pericarpium; Asteris Radix et Rhizoma; Glycyrrhizae Radix et Rhizoma	Cough due to externally contracted wind-heat in children, manifested as cough, expectoration of sputum, fever or nasal congestion, runny nose, slight aversion to wind-cold, red or sore throat, and thin and yellow tongue coating	
31	Yinhuang Qingfei Capsules	**ASA**; Descurainiae Semen Lepidii semen; Ephedrae Herba (processed with honey); Fritillariae Thunbergii Bulbus; Eriobotryae Folium; Isatidis Folium; Acori Tatarinowii Rhizoma; Dioscoreae Nipponicae Rhizoma; Arternisiae Rupestris Herba; Ginkgo Folium; Schisandrae Chinensis Fructus; Aurantii Fructus Imrnaturus; Gypsum; Glycyrrhizae Radix et Rhizoma	Acute attack of chronic bronchitis with the pattern of phlegm-heat obstructing the lung, manifested as cough with yellow and sticky phlegm, oppression in the chest, wheezing, fever, thirst, dry stools, yellow urine, red tongue and yellow, greasy coating	
32	Fengliaoxing Fengshi Dieda Wine	**ASA**; Erycibes Caulis; Cinnamomi Ramulus; Ephedrae Herba; Notopterygii Rhizoma et Radix; Anglicae Sinensis Radix; Chuanxiong Rhiroma; Angelicae Dahuricae Radix; Psoraleae Fructus; Olibanum; Gleditsiae Fructus Abnormalis; Citri Reticulatae Pericarpium; Atractylodis Rhizoma; Magnoliae Officinalis Cortex; Cyperi Rhizoma; Aucklandiae Radix; Aurantii Fructus; Atractylodis Macrocephalae Rhizoma; Dioscoreae Rhizoma; Polygonati Rhizoma; Cuscutae Semen; Foeniculi Fructus; Alismatis Rhizoma; Trogopterori Faeces; Bombycis Feculae; Moutan Cortex; Myrrha	*Bi* disorders due to wind, cold and dampness, numbness of the extremities, soreness and weakness in the lower back and knees; Traumatic injuries and swelling pain due to stasis	
33	San' ao Tablets	**ASA**; Ephedrae Herba; Glycyrrhizae Radix et Rhiroma; Zingiberis Rhizoma Recens	Pattern of wind-cold assailing the lung, manifested as cough, deep hoarse voice, profuse white clear sputum; Acute bronchitis with the symptoms described above	
34	Keke Tablets	**ASA**; Ephedrae Herba; Papaveris Pericarpium; Glycyrrhizae Radix et Rhizoma; Raphani Semen; Platycodonis Radix; Gypsum Fibrosum	Cough, wheezing and shortness of breath	
35	Lingyang Qingfei Granules	ASA (stir-baked); Fritillariae Thunbergii Bulbus; cortex Mori (processed with honey); Peucedani Radix; Ophiopogonis Radix; Asparagi Radix; Trichosanthis Radix; Rehmanniae Radix; Scrophulariae Radix; Dendrobii Herba; Platycodonis Radix; Eriobotryae Folium (processed with honey); Tinosporae Radix; Lonicerae Japonicae Flos; Isatidis Folium; Garedeniae Frucrus; Scutellariae Radix; Isatidis Radix; Moutan Cortex; Menthae Haplocalycis Herba; Glycyrrhizae Radix et Rhiwrna; Rhei Radix et Rhizoma Praeparata; Citri eticulatae Pericarpium; Saigae Tataricae Comu Pulvis	Considerable heat in lung and stomach, with infection of seasonal pathogenic factors; manifested as fever, dizziness, heavy aching limbs, cough, abundant expectoration, swollen sore throat, nosebleed, hemoptysis, dry mouth and tongue	
36	Zhichuanling Injection	**ASA**; Ephedrae Herba; Daturae Flos; Forsythiae Fructus	Wheezing, cough, oppression in the chest, and profuse sputum due to phlegm turbidity obstructing the lung and lung failing to diffuse and downbear; Bronchial asthma and asthmatic bronchitis with the symptoms described above	
37	Niuhuang Qingxin Pills	**ASA (stir-baked)**; Bovis Calculus; Angelicae Sinensis Radix; Chuanxiong Rhizoma; Glycyrrhizae Radix et Rhizoma; Dioscoreae Rhizoma; Scutellariae Radix; Sojae Semen Germinatum; Jujubae Fructus; Atractylodis Macrocephalae Rhizoma (stir-baked); Poria; Platycodi Radix; Saposhnikoviae Radix; Bupleuri Radix; Asini Corii Colla; Zingiberis Rhizoma; Paeoniae Radix Alba; Ginseng Radix et Rhizorna; Massa Medicata Fermentata (stir-baked); Cinnamomi Cortex; Ophiopogonis Radix; Ampelopsis Radix; Typhae Pollen (stir-baked); Moschus or Moschus Artifactus; Bomeolum Syntheticum; Powerdered Buffalo Horn Extract; Saigae Tataricae Cornu; Cinnabaris; Realgar	Pattern of heat entering the pericardium and exuberant heat stirring up wind, manifested as vexation and restlessness in high fever; loss of consciousness and delirious speech; seizures in children due to high fever	
38	Qihuang Tongmi Soft Capsules	**ASA (stir-baked)**; Astragali Radix; Polygoni Multiflori Radix; Angelicae Sinensis Radix; Cistanches Herba; Sesami Semen Nigrum; Juglandis Semen; Rhei Radix et Rhizoma (prepared); Cassiae Semen; Aurantii Fructus Immaturus; Persicae Semen	Functional constipation due to deficiency	
39	Shenyan Jiere Tablets	**ASA (stir-baked)**; Imperatae Rhizorna; Forsythiae Fructus; Schizonepetae Herba; Citri Retiuculatae Pericarpium; Arecae Pericarpium; Alismatis Rhizoma (stir-baked with salt water); Poria; Cinnamomi amulus; Plantaginis Semen (stir-baked); Vignae Semen; Gypsum Fibrosum; Taraxaci Herba; Cicadae Periostracum	Edema caused by wind-heat invasion of the lung, manifested as fever, cold, swelling of the head and face, sore throat, aching limbs, short red urine, thin yellow tongue coating, pulse floating number, and acute nephritis with the symptoms described above	
40	Jinlian Qingre Granules	**ASA (stir-baked)**; Trollii Chinensis Flos; Isatidis Folium; Gypsum Fibrosum; Anemarrhenae Rhizoma; Rehmanniae Radix; Scrophulariae Radix	Pattern of exuberant heat toxin in common cold, manifested as high fever, thirst, dry throat, cough, thick phlegm; influenza and upper respiratory tract infection with the symptoms described above	
41	Jinsang Kaiyin Granules	**ASA (rinsed with boiling water)**; Lonicerae Japonicae Flos; Forsythiae Fructus; Scrophulariae Radix; Isatidis Radix; Paeoniae Radix Rubra; Scutellariae Radix; Mori Folium; Chrysanthemi Flos; Peucedani Radix; Arctii Fructus; Alismatis Rhizoma; Sterculiae Lychnophorae Semen; Bombyx Batryticatus (stir-baked); Cicadae Periostracum; Oroxyli Semen	Swelling and sore of the throat, hoarseness; acute pharyngitis, sub-acute pharyngitis, and laryngitis with the symptoms described above	
42	Fufang Haqing Tablets	ASA; Bufonis Venenum; Astragali Radix; Ginkgo Semen; Asteris Radix et Rhizoma; Peucedani Radix; Aconiti Lateralis Radix Praeparata; Schisandrae Sphenantherae Fructus; Piperis Nigrum Fructus	Pattern of lung deficiency, manifested as coughing and wheezing with profuse sputum; chronic tracheitis, pulmonary emphysema, and asthmatic bronchitis with the symptoms described above	
43	Biaoshi Ganmao Granules	**ASA (stir-baked)**; Perillae Folium; Puerariae Lobatae Radix; Angelicae Dahuricae Radix; Ephedrae Herba; Saposhnikoviae Radix; Platycodonis Radix; Cinnamomi Ramulus; Glycyrrhizae Radix et Rhizoma; Citri Pericarpium Reticulatae; Zingiberis Rhizoma Recens	Common cold of exterior excess wind-cold pattern, manifested as severe chills with mild fever. Absence of sweating, headache, painful stiff nape, clear, runny nose, and cough with white and watery phlegm	
44	Biaoxu Ganmao Granules	**ASA (stir-baked)**; Cinnamomi Ramulus; Puerariae Lobatae Radix; Paeoniae Radix Alba; Zingiberis Rhizoma Recens; Jujubae Fructus	Common cold due to exterior deficiency wind-cold pattern, manifested as fever, chills, sweating, headache, painful stiff nape, cough with white phlegm, stuffy nose and dry retching, thin white coating, and floating and moderate pulse	
45	Ganmao Qingre Chewable Tablets	**ASA**; Schizonepetae Spica; Menthae Haplocalycis Herba; Saposhnikoviae Radix; Radix Bupleuri; Perillae Folium; Puerariae lobatae Radix; Platycodonis Radix; Angelicae Dahuricae Radix; Corydalis bungeanae Herba; Phragmitis Rhizoma	Wind-cold common cold, manifested as headache, fever, chills, general body aches, clear runny nose, cough and dry throat	
46	Ganmao Zhike Syrup	**ASA**; Bupleuri Radix; Lonicerae Flos; Puerariae Lobatae Radix; Artemisiae Annuae Herba; Forsythiae Fructus; Scutellariae Radix; Platycodonis Radix; Menthol	Common cold due to externally contracted wind-heat, manifested as fever, aversion to wind, headache, stuffy nose, swollen sore throat, cough and general malaise	
47	Baikejing Syrup	**ASA (stir-baked)**; Citri Reticulatae Pericarpium; Ophiopogonis Radix; Peucedani Radix; Pinelliae Rhizoma Praeparatum cum Alumine; Scutellariae Radix; Stemonae Radix (processed with honey); Phellodendri Chinensis Cortex; Mori Cortex; Glycyrrhizae Radix et Rhizoma; Ephedrae Herba (processed with honey); Descurainiae Semen Lepidii Semen (stir-baked); Perillae Fructus (stir-baked); Arisaematis Rhizoma (stir-baked); Platycodonis Radix; Trichosanthis Semen (stir-baked)	Cough, expectoration of sputum due to externally contracted wind-heat; Common cold, acute and chronic bronchitis, pertussis with the symptoms. Described above	
48	Baokening Granules	**ASA (stir-baked)**; Perillae Folium; Mori Folium; Peucedani Radix; Fritillariae Thunbergii Bulbus; Ephedrae Herba; Platycodonis Radix; Rhizoma Arisaematis Rhizoma (processed); Citri Reticulntae Pcricarpium; Scutellariae Radix; Indigo Naturalis; Trichosanthis Radix; Aurantii Fructus (stir-baked with bran); Crataegi Fructus (stir-baked); Glycyrrhizae Radix et Rhizoma; Bovis Calculus Artifactus	Pattern of externally contracted wind-cold and internal heat due to food retention in children, manifested as headache with fever, coughing with copious sputum, panting and even wheezing, swollen sore throat, vexation and restlessness	
49	Gejie Dingchuan Capsules	**ASA (stir-baked)**; Gecko; Perillae Fructus (stir-baked); Trichosanthis Semen; Ephedrae Herba: Gypsum Fibrosum; Glycyrrhizae Radix et Rhizoma; Asteris Radix et Rhizoma; Trionycis Carapax (processed with vinegar); Seutellariae Radix; Ophiopogonis Radix; Coptidis Rhizoma; Lilii Bulbus; Gypsum Fibrosum	Persistent cough in consumptive diseases and wheezing in the elderly due to lung heat with Yin deficiency, manifested as shortness of breath, heat vexation, fullness and oppression in the chest, spontaneous sweating, and night sweating	
50	Jieji Ningsou Pills	**ASA;** Perillae Folium; Peucedani Radix; Puerariae Lobatae Radix; Platycodonis Radix; Pinelliae Rhizoma (processed); Citri Reticulatae Pericarpium; Fritillariae Thunbergii Bulbus; Trichosanthis Radix; Scrophulariae Radix; Glycyrrhizae Radix et Rhizoma	Common cold with fever, cough, and profuse sputum in children due to external contraction of wind-cold, and phlegm turbidity obstructing the lung	
51	Chaiyin Mixture	**ASA**; Bupleuri Radix; Lonicerae Japonicae Flos; Scutellariae Radix; Pueraiae Lobatae Radix; Schizonepetae Herba; Artemisiac Annuae Herba; Forsythiae Fructus; Platycodonis Radix; Menthae Haplocalyc is; Houttuyniae Herba	Upper respiratory tract infection due to externally contracted wind-heat, manifested as fever, aversion to wind, headache, sore throat, sweating, stuffy and runny nose, cough, reddened tongue tip and margins with thin yellow coating	
52	Dahuang Zhechong Pills	**ASA (stir-baked)**; Rhei Radix et Rhizoma (processed); Eupolyphaga Steleophaga (stir-baked); Hirudo (processed); Tabanus (removed from wings and feet,stir-baked); Holotrichia Diomphalia (stir-baked); Toxicodendri Resina (calcined); Persicae Semen; Scutellariae Radix; Rehmanniae Radix; Paconiae Alba Radix; Glycyrrhizae Radix et Rhizoma	Abdominal masses and amenorrhea due to internal static blood retention, manifested as abdominal masses, scaly dry skin, dark complexion, tidal fever, emaciation, and amenorrhea	
53	Jinbei Tankeqing Granules	**ASA (stir-baked)**; Fritillariae Thunbcrgii Bulbus; Lonicerae Japonicae Flos; Peucedani Radix; Moil Cortex; Platycodonis Radix; Belamcandae Rhizoma; Ephedrae Herba; Chuanxiong Rhizoma; Glycyrrhizae Radix et Rhizoma	Cough, yellow thick greasy phlegm, wheezing caused by phlegm-heat obstructing the lung; Acute episode of chronic bronchitis with the symptoms described above	
54	Fufang Yigan Pills	**ASA**; Artemisiae Scopariae Herba; lsatidis Radix; Gentianae Radix; Chrysanthemi Indici Flos; Taraxaci Herba; Sophorae Tonkinensis Radix et Rhizoma; Sedi Herba; Cicadae Periostracum; Bovis Calculus Artifactus; Spica Prunellae; Plantaginis Semen; Smilacis Glabrae Rhizoma; Picrorhizae Rhizoma; Moutan Cortex; Salviae Miltiorrhizae Radix et Rhizoma; Carthami Flos; Rhei Radix et Rhizoma; Cyperi Rhizoma; Citri Reticulatae Viride Pericarpium; Aurantii Fructus; Arecae Semen; Gigeriae Galli Endothelium Cornrum; Ginseng Radix et Rhizoma; Cinnamomi Ramulus; Schisaindrae Chinensis Fructus; Bupleuri Radix; Glycyrrhizae Radix et Rhizoma Praeparata Cum Melle	Panern of retained dampness-heat at toxin, manifested as distending pain in the hypochondria, jaundice, dry mouth, bitter taste in the mouth, yellow tongue coating and string-like pulse; Acute and chronic hepatitis with the symptoms described above	

## 6 Toxicological effects including adverse reactions

The main toxic substance in ASA is hydrocyanic acid, which is produced when amygdalin is metabolized. Amygdalin is broken down by β-D-glucosidase into mandelonitrile, which further breaks down into benzaldehyde and hydrocyanic acid. HCN is eventually absorbed into the bloodstream, leading to cyanide poisoning. It is important to note that the toxic doses of amygdalin vary greatly depending on the method of administration. The lethal dose of amygdalin through intravenous injection in humans is 5 g, while oral consumption is 0.5–3.5 mg/kg body weight (Song et al., 2016). When injected intravenously, amygdalin can bypass enzymatic hydrolysis in the gastrointestinal tract, resulting in high blood concentration and detectable amygdalin in the plasma. Additionally, 80% of the injected amygdalin is absorbed by the body within 24 h and eliminated through urine (He et al., 2020). Ingesting 50 ASA consecutively can cause poisoning symptoms in adults, whereas babies can be poisoned by consuming only 5–10 (Chaouali et al., 2013). Cyanide poisoning can lead to rapid hemodynamic and neurological impairment. Studies have shown that hydrocyanic acid can inhibit the activity of cytochrome oxidase in cell mitochondria, causing respiratory inhibition in tissue cells and cell death due to hypoxia. The clinical manifestations of cyanide poisoning depend on the route, duration, dose, and source of exposure. Common symptoms include nausea, vomiting, diarrhea, respiratory failure, hypotension, arrhythmia, cardiac arrest, the odor of bitter almonds, and cherry red skin (Jaszczak-Wilke et al., 2021).

Modern pharmacological research has revealed significant variations in the toxicity of different extracted components of ASA (Table 7). One study found that the median lethal dose (LD50) of lyophilized ASA aqueous extract on Kunming mice was 29.9 g/kg (Song et al., 2016), while another study reported an LD50 of approximately 22.5 g/kg for raw ASA aqueous extract on Kunming mice (Chen and Jia, 2012). However, a separate study administered ASA oil at a dosage of 10 mg/day to Wistar rats for 13 weeks, and no adverse reactions or fatalities were observed (Gandhi et al., 1997). In contrast, when amygdalin was directly administered to Wistar rats, the rats exhibited quadriplegia, muscle-twitching, difficulty in breathing, apnea, and subsequent death, with an LD50 of 880 mg/kg (Adewusi and Oke, 1985). These findings indicate that ASA oil does not exhibit obvious toxicity, whereas ASA water or alcohol extract demonstrates strong toxicity. Furthermore, the toxicity of amygdalin alone is more significant than that of ASA water or alcohol extract.

**TABLE 7:** Toxicological effects including adverse reactions of ASA.

Extract/Compound	Animal/cell line/subject	Minimal toxic concentration/Dose	Toxic and side effects	Reference
Lyophilized ASA aqueous extracts	Male and female Kunming mice	LD50 = 29.9 g/kg	Death	Song et al. (2016)
Raw ASA aqueous extracts	Kunming mice	LD50 = 22.4874 g/kg	Death	Chen and Jia (2012)
ASA Methanol water extracts	Marine bacterium *V. logei* (wild strain)	IC50 = 1.61–2.03 mg/mL ranges from different varieties	Inhibiting bacterial emission	Tareen et al. (2021)
Wild Apricot Oil	Haffkine Wistar strain rats	10 mg per day for 13 weeks	Survival with on clinical signs of any abnormality	Gandhi et al. (1997)
Bitter apricot essential oil	HaCaT (human skin keratinocyte cells)	IC50 = 142.45 μg/mL at 48 h	Suppressing the proliferation	Li et al. (2016)
Amygdalin	Wistar strain rats	LD50 = 880 mg/kg	Quadriplegia, muscle-twitching, difficulty in breathing, apnea and subsequently death	Adewusi and Oke (1985)
Amygdalin	MCF-7 (human breast cancer cells)	IC50 = 5,880.00 μg/mL at 24 h	Inhibiting the proliferation	Ramadan et al. (2019)
Amygdalin	Human breast cancer cells MCF-7 and T47D	IC50 = 39 and 45 mM at 72 h, respectively	Inhibiting the proliferation	Abboud et al. (2019)
Amygdalin	Human breast cancer cells MCF-7 and SR-BR-3	IC50 = 14.2 and 13.7 mg/mL at 24 h, respectively	Inhibiting the proliferation	Moradipoodeh et al. (2020)
Amygdalin-Z _HER2_ affibody conjugate	Human breast cancer cells MCF-7 and SR-BR-3	IC50 = 8.27 and 19.8 mg/mL at 24 h, respectively	Inhibiting the proliferation	Moradipoodeh et al. (2020)
Amygdalin-folic acid nanoparticles	MCF-10A (human normal mammary epithelial cells)	IC50 = 180.3 μg/mL at 24 h	Anti-proliferative activity	Askar et al. (2023)
Amygdalin-folic acid nanoparticles	Human breast cancer cells MCF-7 and MDA-MB-231	IC50 = 79.8 and 94.9 μg/mL at 24 h, respectively	Anti-proliferative activity	Askar et al. (2023)
Amygdalin	Huh-7 (human liver cancer cells)	IC50 = 11.587, 1.9, 0.625 mM at 24, 48 and 72 h, respectively	Inhibiting the proliferation in a dose and time-dependent manner	Mamdouh et al. (2021)
Amygdalin	HepG-2 (human liver cancer cells)	IC50 = 41.86, 1.224, 0.089 mM at 24, 48 and 72 h, respectively	Inhibiting the proliferation in a dose and time-dependent manner	Mamdouh et al. (2021)
Amygdalin	HepG-2 (human liver cancer cells)	IC50 = 2,691.54 μg/mL at 24 h	Inhibiting the proliferation	Ramadan et al. (2019)
Amygdalin	HepG-2 (human liver cancer cells)	IC50 = 458.10 mg/mL at 48 h	Inhibiting the proliferation	Zhou et al. (2012)
Amygdalin+ β-D-glucosidase	HepG-2 (human liver cancer cells)	IC50 = 3.2 mg/mL at 24 h	Inhibiting the proliferation	Zhou et al. (2012)
Amygdalin	HCT116 (human colon cancer cells)	IC50 = 6,309.57 μg/mL at 24 h	Inhibiting the proliferation	Ramadan et al. (2019)
CuO-TiO_2_-Chitosan-Amygdalin Nanocomposites	MOLT4 (human acute lymphoblastic leukemia cells)	IC50 = 38.41 μg/mL at 24 h	Inhibiting the proliferation	Elderdery et al. (2022)
Amygdalin	PC12 (rat pheochromocytoma cells) and MDCK (Madin Darby canine kidney cells)	IC50 = 38.53 and 63.97 μM at 48 h, respectively	Inhibiting the proliferation	Song et al. (2016)
Amygdalin+ β-D-glucosidase	PC12 (rat pheochromocytoma cells) and MDCK (Madin Darby canine kidney cells)	IC50 = 5.97 and 3.93 μM at 48 h, respectively	Inhibiting the proliferation	Song et al. (2016)

β-D-glucosidase plays a crucial role in the hydrolysis process of amygdalin. When amygdalin was administered alone, the IC50 of HepG-2 was 458.10 mg/mL. However, co-administration of amygdalin with β-D-glucosidase resulted in a more than 100-fold decrease in IC50 to 3.2 mg/mL, highlighting the critical role of β-D-glucosidase in the pathway of amygdalin poisoning (Zhou et al., 2012). Similarly, there was a notable difference in the IC50 values of PC12 and MDCK cells when amygdalin was administered alone or in combination with β-D-glucosidase. The IC50 of PC12 cells decreased from 35.83 to 5.97 μM, and the IC50 of MDCK cells decreased from 63.97 to 3.93 μM (Song et al., 2016). Although amygdalin itself is stable, it becomes highly toxic after hydrolysis by β-D-glucosidase. Unfortunately, β-D-glucosidase is widely present in humans, animals, plant seeds, and microorganisms. Therefore, it is crucial to explore methods for attenuating amygdalin poisoning and implementing preventive measures.

Traditional Chinese medicine suggests that ASA should undergo processing before use to inhibit the activity of amygdalin and preserve its properties. The 2020 edition of the Chinese Pharmacopoeia states that the main methods for processing and detoxifying ASA include the Clear fried method and the Chan method (Wei et al., 2023). It has been discovered that the combined use of *ephedare herba*—herbaceous stems of *Ephedra sinica* Stapf (Ephedraceae) with ASA effectively reduces the toxicity of ASA without impacting the amygdalin content. When mice were orally administered ASA alone, the LD50 was found to be 29.9 g/kg. However, when different ratios of *ephedare herba* and ASA (MX (4:1), MX (2:1), MX (1:1), MX (1:2), and MX (1:4)) were orally administered, the LD50 of mice was 87.9, 81.6, 81.4, 64.6, and 59.3 g/kg respectively, indicating the detoxification effect of *Ephedra sinica Stapf* on ASA. Furthermore, the HPLC method was used to measure the difference in amygdalin content among the mentioned groups above. The content of amygdalin in the ASA water extract was found to be 11.77 mg/g. However, co-extraction with ephedra did not result in significant differences in the amygdalin content (Song et al., 2016).

Another detoxification method for ASA has recently been reported. The method involves soaking ASA powder in a 25% sodium chloride solution for 12 h, followed by rinsing with tap water until the liquid becomes clear. This process is repeated once, and then the ASA powder is soaked again in the 25% sodium chloride solution for another 12 h. After rinsing until the liquid is clear, the ASA powder is dried at 45° for 36 h, resulting in the detoxified ASA. This method effectively eliminates the toxic component HCN and significantly reduces the levels of antinutrient factors such as phytates, phytate phosphorus, and oxalate by 71.83%, 23.92%, and 38% respectively compared to raw ASA. The fat content and crude fiber content do not show significant changes. However, there is a reduction in the contents of Vitamin C, β-carotene, minerals, and protein to varying degrees (Tanwar et al., 2018). Overall, this method can be employed in ASA oil and functional food production. Nevertheless, further research is needed to fully explore the medicinal potential of ASA and investigate the effects of different processing methods on ASA.

## 7 Pharmacokinetic profile

Studies on the pharmacokinetics of ASA primarily focus on amygdalin and its metabolite prunasin (Table 8). When ASA water extract is administered orally, amygdalin and prunasin can be detected in the plasma of rats, exhibiting significantly different pharmacokinetic parameters, particularly in terms of the maximum concentration (C_max_). After oral administration of ASA water extract, amygdalin is rapidly absorbed with a T_max_ at 0.5 h and a C_max_ at 223.6 ng/mL. Subsequently, a substantial amount of amygdalin is hydrolyzed to prunasin within a short time, with a T_max_ of 0.58 h and a C_max_ of 5,212.8 ng/mL (Song et al., 2015). The volume of distribution/bioavailability (Vz/F) of amygdalin is 196.8 L/kg, while the Vz/F of prunasin is 15.9 L/kg, indicating that amygdalin exhibits high tissue distribution specificity and may be concentrated in certain organs compared to prunasin (Helmy et al., 2013). Recent research revealed that the concentration of amygdalin in lung tissue (309.335 ± 13.662 ng/g) was significantly higher than in plasma (44.774 ± 7.397), heart (23.693 ± 6.097), liver (43.391 ± 5.963), spleen (53.745 ± 6.584), and kidney (55.373 ± 4.467) (Yang et al., 2021), suggesting that amygdalin may be concentrated in lung tissue. The elimination half-life (t_1/2_) of amygdalin and prunasin are 1.15 ± 0.26 h and 2.21 ± 0.52 h, respectively. Similarly, the mean residence time (MRT) for amygdalin and prunasin are 1.33 ± 0.23 h and 1.57 ± 0.22 h, respectively (Song et al., 2015). This observation can be attributed to the hydrolysis of β-D-glucosidase. Additionally, the clearance/bioavailability (CLz/F) of amygdalin is significantly higher at 121.1 ± 31.4 L/kg·h compared to prunasin, which has a CLz/F of only 5.1 ± 0.9 L/kg·h. This difference may be linked to the higher blood concentrations of prunasin. It is worth noting that amygdalin exists in two isomers, D and L, with the latter being stable only at temperatures higher than 40°C (Wahab et al., 2015). After administration of ASA water extract, the plasma concentrations of the two isomers are almost the same, with values of 147.8 ± 34.9 and 138.7 ± 32.4 ng/mL, respectively. However, their metabolites, D-Prunasin and L-Prunasin, exhibit significant differences in concentration, with values of 2,101.4 ± 453.0 and 3,561.2 ± 619.8 ng/mL, respectively. Importantly, the content of L-Prunasin is considerably higher than that of D-Prunasin, indicating stereoselective metabolism of amygdalin. Besides, the bioavailability of amygdalin was found to be only 0.19% ± 0.08% when orally administered to rats, suggesting that amygdalin may have undergone degradation before reaching the intestinal tract. In contrast, prunasin exhibited a higher bioavailability of 64.91% ± 6.30% when administered orally. These findings indicate that amygdalin undergoes deglycosylation metabolism (Zhang et al., 2022b).

**TABLE 8:** Pharmacokinetic profiles of ASA.

Animal	Drug administrated	Dose	Compound	Pharmacokinetic parameters	Reference
Male SD rats	ASA aqueous extracts (oral administration)	3 g/kg	Amygdalin	T_max_(h): 0.50 ± 0.00	Song et al. (2015)
				C_max_(ng/mL): 223.6 ± 32.1	
				AUC_0–t_(ng·h/mL): 286.5 ± 66.8 t_1/2_(h): 1.15 ± 0.26	
				MRT_0–t_(h): 1.33 ± 0.23	
				Vz/F(L/kg): 196.8 ± 47.8	
				CLz/F(L/kg·h): 121.1 ± 31.4	
			D-Amygdalin	T_max_(h): 0.50 ± 0.00	
				C_max_(ng/mL): 112.1 ± 14.9	
				AUC_0–t_(ng·h/mL): 147.8 ± 34.9 t_1/2_(h): 1.37 ± 0.48	
				MRT_0–t_(h): 1.34 ± 0.22	
				Vz/F(L/kg): 231.6 ± 74.6	
				CLz/F(L/kg·h): 120.4 ± 31.5	
			L-Amygdalin	T_max_(h): 0.50 ± 0.00	
				C_max_(ng/mL): 111.5 ± 18.4	
				AUC_0–t_(ng·h/mL): 138.7 ± 32.4 t_1/2_(h): 1.18 ± 0.28	
				MRT_0–t_(h): 1.31 ± 0.24	
				Vz/F(L/kg): 200.4 ± 62.6	
				CLz/F(L/kg·h): 119.9 ± 31.9	
			Prunasin	T_max_(h): 0.58 ± 0.20	
				C_max_(ng/mL): 5,212.8 ± 777.1	
				AUC_0–t_(ng·h/mL): 6919.9 ± 1,455.7 t_1/2_(h): 2.21 ± 0.52	
				MRT_0–t_(h): 1.57 ± 0.22	
				Vz/F(L/kg): 15.9 ± 3.0	
				CLz/F(L/kg·h): 5.1 ± 0.9	
			D-Prunasin	T_max_(h): 0.58 ± 0.20	
				C_max_(ng/mL): 1,674.8 ± 227.1	
				AUC_0–t_(ng·h/mL): 2101.4 ± 453.0 t_1/2_(h): 2.20 ± 0.64	
				MRT_0–t_(h): 1.51 ± 0.22	
				Vz/F(L/kg): 27.8 ± 9.5	
				CLz/F(L/kg·h): 8.8 ± 1.5	
			L-Prunasin	T_max_(h): 0.62 ± 0.21	
				C_max_(ng/mL): 3561.2 ± 619.8	
				AUC_0–t_(ng·h/mL): 4811.1 ± 1,056.9 t_1/2_(h): 2.22 ± 0.62	
				MRT_0–t_(h): 1.59 ± 0.23	
				Vz/F(L/kg): 11.1 ± 2.7	
				CLz/F(L/kg·h): 3.5 ± 0.7	
Male and female SD rats	Amygdalin (oral administration)	100 mg/kg	Amygdalin	T_max_(h): 0.25	Qin et al. (2021)
				C_max_(ng/mL): 93.871	
				AUC_0–t_(ng·h/mL): 73.595	
				AUC_0-∞_(ng·h/mL): 74.133 t_1/2_(h): 1.21	
				MRT(h): 1.91	
Male Wistar rats	Amygdalin (oral administration)	5 mg/kg	Amygdalin	T_max_(min): 14.00 ± 10.84	Zhang et al. (2022b)
				C_max_(ng/mL): 23.08 ± 5.08	
				AUC_0–t_(1,569.22): 1,391.77 ± 560.91	
				AUC_0-∞_(ng·min/mL): 1,569.22 ± 650.62 k_e_(/min): 0.030 ± 0.010	
				t_1/2_(min): 28.76 ± 7.25	
				MRT(min): 53.33 ± 10.05	
				Vd(mL/kg): 140,028.28 ± 27,425.92	
				CL(mL/min/kg): 3636.14 ± 1,375.61	
				F (%): 0.19 ± 0.08	
			Prunasin	T_max_(min): 22.00 ± 4.47	
				C_max_(ng/mL): 1835.12 ± 268.09	
				AUC_0–t_(ng·min/mL): 99,732.22 ± 17,256.2	
				AUC_0-∞_(ng·min/mL): 103,913.17 ± 14,202.48 k_e_(/min): 0.015 ± 0.002	
				t_1/2_(min): 47.79 ± 5.72	
				MRT(min): 51.27 ± 2.62	
				Vd(mL/kg): 3336.43 ± 741.42	
				CL(mL/min/kg): 48.19 ± 7.58	
	Amygdalin (intravenous administration)	5 mg/kg	Amygdalin	T_max_(min): 2.00 ± 0.00	
				C_max_(ng/mL): 34,763.84 ± 18,057.68	
				AUC_0–t_(ng·min/mL): 731,268.98 ± 109,541.87	
				AUC_0-∞_(ng·min/mL): 731,909.80 ± 109,917.01 k_e_(/min): 0.010 ± 0.004	
				t_1/2_(min): 67.93 ± 24.72	
				MRT(min): 39.42 ± 5.95	
				Vd(mL/kg): 680.71 ± 257.40	
				CL(mL/min/kg): 6.97 ± 1.12	
			Prunasin	T_max_(min): 69.00 ± 29.24	
				C_max_(ng/mL): 88.64 ± 22.18	
				AUC_0–t_(ng·min/mL): 6754.24 ± 1,304.65	
				AUC_0-∞_(ng·min/mL): 81,926.10 ± 8557.72 k_e_(/min): 0.004 ± 0.002	
				t_1/2_(min): 214.40 ± 96.83	
				MRT(min): 281.70 ± 103.83	
				Vd(mL/kg): 186,646.54 ± 74,474.47	
				CL(mL/min/kg): 638.28 ± 167.77	
	Prunasin (oral administration)	5 mg/kg	Prunasin	T_max_(min): 16.00 ± 5.48	
				C_max_(ng/mL): 2912.06 ± 433.45	
				AUC_0–t_(ng·min/mL): 134,797.34 ± 13,091.48	
				AUC_0-∞_(ng·min/mL): 135,731.78 ± 12,982.12 k_e_(/min): 0.013 ± 0.005	
				t_1/2_(min): 63.48 ± 31.17	
				MRT(min): 46.80 ± 4.65	
				Vd(mL/kg): 3369.66 ± 1,532.62	
				CL(mL/min/kg): 37.02 ± 3.80	
				F (%): 64.91 ± 6.30	
	Prunasin (intravenous administration)	5 mg/kg	Prunasin	T_max_(min): 2.00 ± 0.00	
				C_max_(ng/mL): 6926.50 ± 1952.91	
				AUC_0–t_(ng·min/mL): 207,670.12 ± 22,295.20	
				AUC_0-∞_(ng·min/mL): 208,663.12 ± 22,869.27 k_e_(/min): 0.010 ± 0.004	
				t_1/2_(min): 69.42 ± 22.68	
				MRT(min): 43.94 ± 3.33	
				Vd(mL/kg): 2469.24 ± 979.45	
				CL(mL/min/kg): 25.05 ± 2.29	
3 males and 4 females aged 63.3 ± 9.1 years	Almond skin polyphenols (oral administration)	450 mg	Catechin	T_max_(h): 1.4 ± 0.2	Chen et al. (2019)
				C_max_(ng/mL): 44.3 ± 15.6	
			Naringenin	T_max_(h): 3.3 ± 0.5	
				C_max_(ng/mL): 19.3 ± 8.2	
			total flavonoids	T_max_(h): 1.7 ± 0.3	
				C_max_(ng/mL): 82.3 ± 17.6	

Notes: T_max_, time to peak concentration; AUC_0–t_, area under the plasma concentration curve (0-t); AUC_0-∞_, area under the plasma concentration curve (0-∞); CL, body clearance; CLz/F, clearance/bioavailability; C_max_, maximum concentration; F (%), bioavailability; ke, elimination rate constant; MRT, mean residence time; t_1/2_, elimination half-time; Vd, volume of distribution; Vz/F, volume of distribution/bioavailability.

Changes in the oral dose of amygdalin lead to variations in its pharmacokinetic parameters. For instance, when rats were orally administered 5 mg/kg of amygdalin, the following parameters were observed: T_max_ was 14 min, C_max_ was 23.08 ng/mL, area under the plasma concentration curve (0-t) (AUC_0–t_) was 1,391.77 ng min/mL, area under the plasma concentration curve (0-∞) (AUC_0-∞_) was 1,569.22 ng min/mL, t_1/2_ was 28.76 min, and MRT was 53.33 min (Zhang et al., 2022b). However, when the dosage was increased to 100 mg/kg, the following parameters were observed: T_max_ was 0.25 h, C_max_ was 93.871 ng/mL, AUC_0-t_ was 73.595 ng h/mL (equivalent to 4415.7 ng min/mL), AUC_0-∞_ was 74.133 ng h/mL (equivalent to 4447.98 ng min/mL), t1/2 was 1.21 h, and MRT was 1.91 h (Yang et al., 2021). Notably, there is little difference in T_max_ between the doses of 5 mg/kg and 100 mg/kg, suggesting that the absorption speed of amygdalin may not be affected by dosage. However, as the dose increases, C_max_, AUC, t_1/2_, and MRT of amygdalin significantly increase. This indicates that higher doses lead to higher peak concentrations of amygdalin and slower elimination, resulting in a longer presence of amygdalin in the body.

Different drug-delivery routes have a significant impact on the absorption, distribution, and elimination of amygdalin. When amygdalin is injected intravenously at a dose of 5 mg/kg, it reaches its T_max_ within 2 min, while oral administration takes 14 min. The C_max_ after intravenous injection is 34,763.84 ± 18,057.68 ng/mL, compared to only 23.08 ng/mL with oral administration. These indicate that amygdalin is absorbed more rapidly and reaches higher peak plasma concentrations when administered intravenously. Furthermore, the volume of distribution (Vd) for intravenous injection and oral administration is 680.71 ± 257.40 mL/kg and 140,028.28 ± 27,425.92 mL/kg, respectively. This suggests that when amygdalin is administered intravenously, it is primarily distributed in the plasma, whereas after oral administration, it becomes more concentrated. Additionally, the t_1/2_ of intravenous administration (67.93 ± 24.72 h) is longer than that of oral administration (28.76 ± 7.2 h), and the MRT of intravenous administration (39.42 ± 5.95 min) is shorter than that of oral administration (53.33 ± 10.05 min). These findings indicate that amygdalin remains in the body for a longer duration when administered intravenously (Zhang et al., 2022b).

In addition, the pharmacokinetics of flavonoids in ASA were also investigated. After orally administering 450 mg of ASA skin polyphenols, the plasma was found to contain catechin and naringenin. The T_max_ and C_max_ values for catechin were 1.4 ± 0.2 h and 44.3 ± 15.6 ng/mL, respectively. For naringenin, the T_max_ and C_max_ values were 3.3 ± 0.5 h and 19.3 ± 8.2 ng/mL, respectively. Moreover, the C_max_ of total flavonoids was 82.3 ± 17.6 ng/mL, which exceeded the levels of catechin and naringenin. This suggests the presence of other unidentified flavonoids in ASA.

## 8 Conclusion and future perspectives

Natural medicinal plants have shown significant benefits in treating a range of diseases, including COVID-19 (Setayesh et al., 2022), respiratory diseases (Hajimonfarednejad et al., 2023), mental health disorders such as anxiety and insomnia (Motti and de Falco, 2021), hyperlipidemia (Hashempur et al., 2018), and common fungal infections (Amini et al., 2023). These plants are characterized by their multi-component and multi-target nature, making them vital in the treatment of various illnesses. ASA, a Chinese herbal medicine with a long history of medicinal use, is rich in phytochemical ingredients, active substances, and nutrients. It serves as both a medicinal drug and nutraceutical, with great potential for broad application.

Here, we comprehensively reviewed the phytochemical composition, pharmacological activities, clinical applications, toxicology, and pharmacokinetics studies of ASA. The present study offers a comprehensive summary of the phytochemical composition of ASA, categorizing it into distinct structural types for the first time. It also provides a systematic overview of the pharmacological activities and mechanism of action of ASA. Moreover, the study includes a novel compilation of various detoxification methods before ASA administration, along with an analysis of the alterations in pharmacokinetic parameters after ASA administration. The current research primarily focuses on assessing the anticancer potential of various extracts of ASA and its main component, amygdalin. To date, researchers have successfully isolated and identified 170 chemical components from different ASA extracts. Extensive *in vivo* and *in vitro* pharmacological studies have revealed that amygdalin and polyphenols in ASA possess a wide range of pharmacological activities. Furthermore, ASA fatty oil and volatile oil also exhibit specific pharmacological activities in the treatment of certain diseases.

However, there are some aspects worth noting and requiring further research. 1) Amygdalin in ASA exhibits excellent anti-cancer activity in various cell lines. However, most studies conducted so far have been *in vitro*, with only a few *in vivo* experiments. Therefore, more preclinical research and translation into clinical studies are needed. 2) It is important to note that ASA is toxic, as amygdalin can be metabolized by β-D-glucosidase, leading to cyanide poisoning. There is limited research on detoxification methods of ASA, and current methods may result in the loss of some active ingredients. Therefore, future research should focus on developing efficient detoxification methods that also preserve the therapeutic properties of ASA. 3) While more than 170 chemical components have been identified in ASA, the pharmacological evaluation has been limited to a few compounds such as amygdalin, its metabolites, total polyphenols, and total volatile oils. Thus, there is an urgent need for in-depth studies on the phytochemistry and pharmacological properties of ASA, particularly the mechanism of action of its bioactive components. 4) ASA and its compounds have shown promising therapeutic effects in the treatment of respiratory diseases in both *in vivo* and *in vitro* studies. Some ASA-containing formula preparations have been included in the 2020 edition of the Chinese Pharmacopoeia. Therefore, further investigation into the pharmacological activities and mechanisms of action of these compounds is warranted. 5) Currently, there is a lack of pharmacokinetic data on different ASA extracts and active compounds. Conducting more pharmacokinetic studies on crude ASA extracts and active compounds is crucial for the rational clinical use and development of new drugs.

ASA, a Chinese herbal medicine, is known for its medicinal and food uses. It is rich in phytochemicals and nutrients, making it clinically valuable and potentially useful for food development. Further research is needed to investigate the pharmacological activities of different components of ASA and understand their underlying mechanisms. This study offers a comprehensive analysis of ASA, providing valuable insights for researchers to improve their understanding of ASA and promote the development of ASA as a clinical drug and healthy food.

## Abbreviations

ABTS 2′-Azinobis-(3-ethylbenzthiazoline-6-sulphonate); AchE acetyl cholinesterase; ASA Armeniacae semen amarum; ACE angiotensin-converting enzyme; ALI acute lung injury; ALT alanine aminotransferase; AST aspartate aminotransferase; AUC_0–t_ area under the plasma concentration curve (0-t); AUC_0-∞_ area under the plasma concentration curve (0-∞); CAT catalase; CL body clearance; CLz/F clearance/bioavailability; C_max_ maximum concentration; CNKI China National Knowledge Infrastructure; COPD chronic obstructive pulmonary disease; COX-2 cyclooxygenase-2; DPPH 2,2-diphenyl-1-picrylhydrazyl; DM diabetes mellitus; DMBA 2,2′-Bis (hydroxymethyl)butyric; EMT epithelial-mesenchymal transition; F (%) bioavailability; FRAP ferric reducing anti-oxidant power; GAE gallic acid equivalent; GGT gamma-glutamyl transferase; GSH glutathione; GST glutathione S-transferase; HbA1c hemoglobin A1C; HDL-C high-density lipoprotein cholesterol; IC50 half maximal inhibitory concentration; iNOS inducible nitric oxide synthase; ke elimination rate constant; LD50 median lethal dose; LDL-C low-density lipoprotein cholesterol; LPO lipid peroxide; MDA malondialdehyde; MRT mean residence time; PGE2 prostaglandin E2; ROS reactive oxygen species; SOD superoxide dismutase; t_1/2_ elimination half-life; TAC total anti-oxidant capacity; T_max_ time to peak concentration; Vd volume of distribution; Vz/F distribution/bioavailability.
